# Inhibition of Calcium‐Dependent Lipid Droplets Relocation of ACSL4‐PKCβ‐ALOX15 Complex Alleviates Ferroptosis and Acute Pancreatitis

**DOI:** 10.1002/advs.202515768

**Published:** 2026-01-27

**Authors:** Guoyuan Hou, Jing Luan, Xiaoyong Xu, Jianhua Qin, Shuang Ma, Jiyuan He, Na Sun, Wei Zhang, Minghui Gao

**Affiliations:** ^1^ The HIT Center for Life Sciences School of Life Science and Technology Harbin Institute of Technology Harbin Heilongjiang China; ^2^ Hangzhou HuaAn Biotechnology Co., Ltd Hangzhou Zhejiang China; ^3^ Department of Microbiology and Immunology Weill Cornell Medicine New York New York USA

**Keywords:** ACSL4, acute pancreatitis, ALOX15, calcium, ferroptosis, lipid droplets, PKCβ

## Abstract

Ferroptosis, an iron‐dependent form of programmed cell death driven by toxic lipid peroxide accumulation, plays a critical role in various diseases, making its modulation a promising therapeutic strategy. In this study, we identified several L‐type calcium channel blockers as novel inhibitors of ferroptosis. We further elucidated that calcium‐dependent activation of PKCβ drives ferroptosis by phosphorylating two key enzymes, ACSL4 and ALOX15, at multiple sites. We generated phosphorylation‐specific antibodies targeting these sites and confirmed their specificity in the context of ferroptosis. Furthermore, upon induction of ferroptosis, the ACSL4‐PKCβ‐ALOX15 complex relocates to lipid droplets, highlighting a critical role of lipid droplets in ferroptosis. Notably, elevated PKCβ levels enhance the efficacy of ferroptosis‐inducing cancer therapies, while inhibition of the Ca^2^^+^‐PKCβ signaling pathway protects against acute pancreatitis by suppressing ferroptosis. These findings underscore the therapeutic potential of targeting Ca^2^^+^‐PKCβ‐mediated ferroptosis, offering new avenues for the treatment of cancer and acute pancreatitis.

## Graphical abstract

This study identifies L‐type calcium channel blockers as novel ferroptosis inhibitors. It reveals that PKCβ, activated in calcium dependent manner, phosphorylates and activates ACSL4 and ALOX15, relocating them to lipid droplets to promote lethal lipid peroxidation and ferroptosis. Targeting the Ca^2^^+^‐PKCβ pathway enhances cancer therapy efficacy and protects against acute pancreatitis, highlighting its broad therapeutic potential.

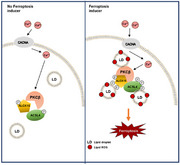

## 1 Introduction

Ferroptosis is a form of regulated necrosis characterized by iron‐dependent accumulation of toxic lipid peroxides. During ferroptosis, the GPX4 enzyme is unable to catalyze the reduction of excessive lipid peroxides, leading to a buildup of lethal reactive oxygen species (ROS) [1, 2, 3, 4]. This process has significant implications for various diseases and physiological processes. Ferroptosis serves as a tumor suppressor mechanism, and its dysfunction can contribute to cancer development [5, 6, 7]. Interestingly, certain cancer cells with specific mutations exhibit increased susceptibility to ferroptosis‐inducing treatments [8, 9, 10, 11, 12]. Beyond oncology, growing evidence suggests that ferroptosis plays a crucial role in organ injuries, particularly those related to ischemia [1, 4, 13]. The process's involvement in extensive lipid peroxidation links it to various other pathological conditions as well. Given its wide‐ranging impact, pharmacological modulation of ferroptosis holds significant promise for developing novel therapeutic strategies. These approaches could potentially address drug‐resistant cancers, organ injuries, and other diseases associated with excessive lipid peroxidation.

Unrestrained lipid peroxidation is the hallmark of ferroptosis [3]. ACSL4 (Acyl‐CoA Synthetase Long‐Chain Family Member 4) plays a critical role in the activation of long‐chain polyunsaturated fatty acids (PUFAs) by converting them into their acyl‐CoA derivatives [14, 15]. These activated fatty acids are then incorporated into phospholipids (PL), such as phosphatidylethanolamine (PE) and phosphatidylinositol (PI), which are essential components of cell membranes. Certain lipoxygenases (LOXs), which are non‐haeme iron‐dependent dioxygenases targeting PUFAs, can directly oxygenate PUFAs and PUFA‐containing lipids in biological membranes [16, 17]. Especially, ALOX15 (Arachidonate 15‐Lipoxygenase) catalyzes the oxidation of PUFAs and PUFA‐containing lipids at the 15th carbon, particularly arachidonic acid (AA) and adrenic acid, into lipid hydroperoxides (e.g., 15‐hydroperoxyeicosatetraenoic acid, 15‐HPETE), making it a critical player in the peroxidation of membrane lipids during ferroptosis [18, 19]. Membranes containing a high PUFA‐PL content should be particularly vulnerable to peroxidation. Actually, membranes in different subcellular organelles such as mitochondria [6], endoplasmic reticulum [15], peroxisomes [20], lysosomes [21, 22], and the plasma membrane [3] have been reported to be relevant to lipid peroxidation during ferroptosis, although the precise cellular membranes remain to be investigated.

Calcium plays viral roles in regulated cell death [23], including apoptosis [24, 25], necroptosis [26] and pyroptosis [27]. Elevation of cytosolic calcium preceding cell lysis in ferroptosis has been observed. Ca^2+^ fluxes during ferroptosis induce the activation of the ESCRT‐III‐dependent membrane repair machinery for membrane repair, which delays the kinetics of cell death likely by counter balancing membrane damage, suggesting anti‐ferroptosis function of calcium [28]. Furthermore, mitochondrial Ca^2^^+^ levels have been implicated in directly influencing the cellular response to ferroptosis [29]. Despite the remarkable progress made in the field [30, 31], our understanding of the contribution of Ca^2+^ signals to ferroptosis remains limited.

Here, we identified multiple L‐type calcium channel blockers as novel ferroptosis suppressors. We further demonstrated that calcium‐dependent PKCβ activation promotes ferroptosis by phosphorylating both ACSL4 and ALOX15 at multiple sites. We developed phosphorylation antibodies against these sites and confirmed their specificity in ferroptosis. Upon ferroptosis induction, the PKCβ‐ACSL4‐ALOX15 complex translocate to lipid droplets, suggesting a crucial role of lipid droplets in ferroptosis. Importantly, a high level of PKCβ enhances ferroptosis‐inducing cancer therapy, while inhibition of the Ca^2^^+^‐PKCβ signaling pathway protects the pancreas from acute pancreatitis by suppressing ferroptosis. These findings suggest that modulation of Ca^2^^+^‐PKCβ‐mediated ferroptosis holds significant promise for developing novel therapeutic strategies for these diseases.

## 2 Results

### 2.1 Multiple L‐Type Calcium Channel Blockers Suppress Ferroptosis

**FIGURE 1:**
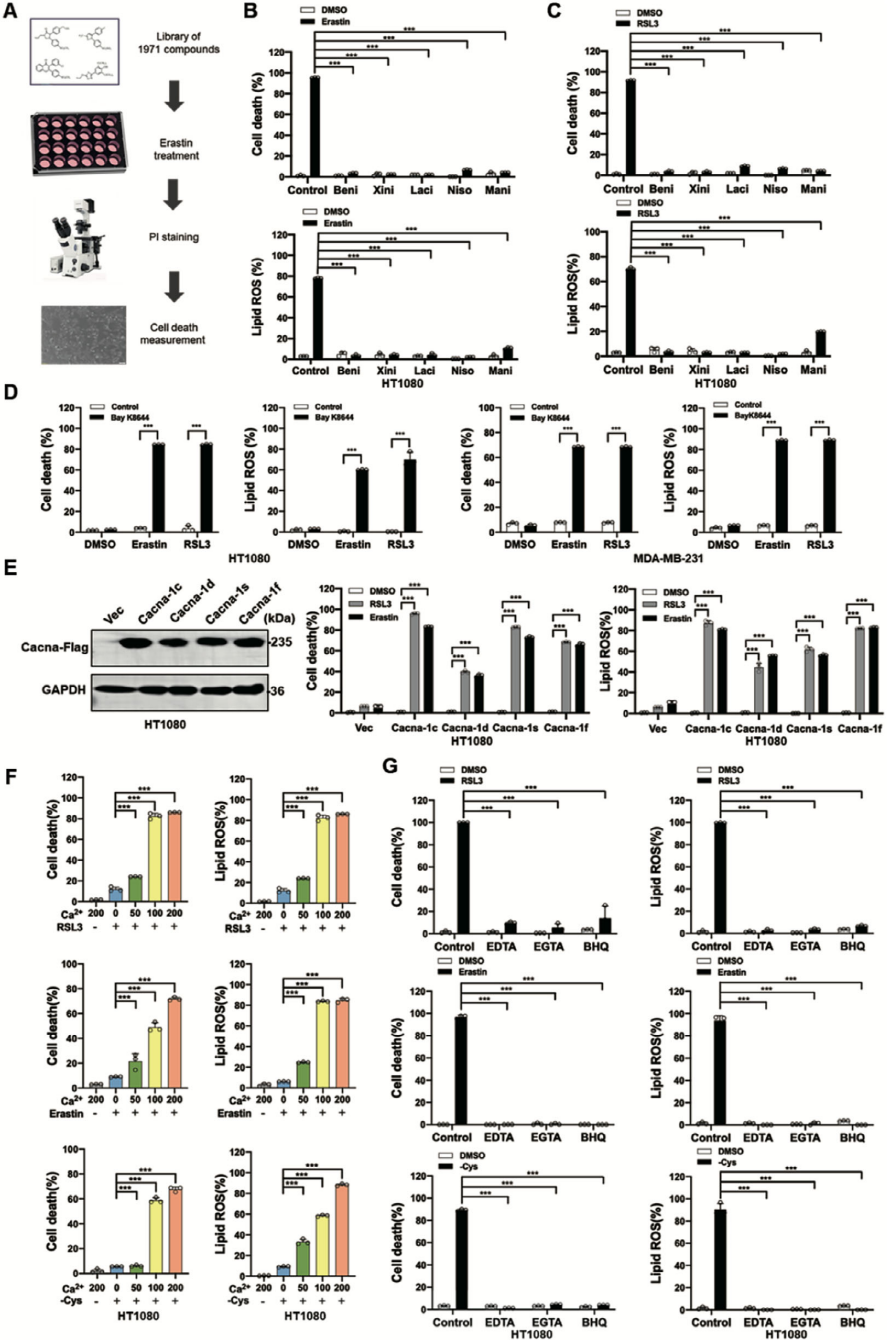
Multiple L‐type calcium channel blockers suppress ferroptosis A) Overview of ferroptosis modifiers screening. HT1080 Cells seeded in 24‐well plates overnight were treated with erastin (10 µM) plus an individual small compound from the library for 16 h. Cell death was analyzed by PI staining followed by microscopy or flow cytometry analysis. B) L‐type calcium channel blockers treatment suppress erastin induced ferroptosis and lipid peroxidation. HT1080 cells were treated with erastin (10 µM) with or without L‐type calcium channel blockers for 20 h, cells were then stained with Propidium Iodide (PI) and cell death was analyzed by flow cytometry. Cells treated as indicated were stained with BODIPY 581/591 C11, and lipid ROS levels were measured by flow cytometry. Beni: Benidipine (50 µM), Xini: Xinidipine (20 µM), Laci: Lacidipine (40 µM), Niso: Nisoldipine (50 µM) and Mani: Manidipine (50 µM). C) L‐type calcium channel blockers treatment suppress RSL3 induced ferroptosis and lipid peroxidation. HT1080 cells were treated with RSL3 (1 µM) with or without L‐type calcium channel blockers for 10 h, then cell death and lipid ROS were measured as described in Figure 1B. D) L‐type calcium channel activator Ray K8644 promotes ferroptosis. HT1080 cells or MDA‐MB‐231 cells were treated with erastin (10 µM) or RSL3 (1 µM) with or without Ray K8644 (50 µM) for 18 h, then cell death and lipid ROS were measured as described in Figure 1B. E) Overexpression of L‐type calcium channel promotes ferroptosis and lipid peroxidation. Western blot images confirmed the expression of the indicated protein. HT1080 cells with overexpressed individual L‐type calcium channel were treated with RSL3 (1 µM, for 10 h) or erastin (10 µM, for 20 h), then cell death and lipid ROS were measured as described in Figure 1B. F) Calcium is required for ferroptosis in a dose dependent manner. HT1080 cells were treated with RSL3 (1 µM, for 10 h), erastin (10 µM, for 20 h) or cystine starvation (for 30 h) with different dose calcium (mg/ml), then cell death and lipid ROS were measured as described in Figure 1B. G) Calcium chelators treatment blocked ferroptosis and lipid peroxidation. HT1080 cells were treated with RSL3 (1 µM, for 10 h), erastin (10 µM, for 20 h) or cystine starvation (for 30 h) with or without calcium chelators, then cell death and lipid ROS were measured as described in Figure 1B. EDTA‐AM (40 µM), EGTA‐AM (50 µM), BHQ (50 µM). Data are derived from three independent experiments, and each value represents the mean ± SD. ^*^p < 0.05, ^**^p < 0.01, ^***^p < 0.001, *t* test. (*n* = 3).

To identify potent ferroptosis modulators, we conducted a high‐throughput screen using the DiscoveryProbe FDA‐approved Drug Library, which comprises 1,971 small‐molecule compounds. The screen was performed in human fibrosarcoma HT1080 cells subjected to erastin‐induced ferroptosis, with cell death assessed via propidium iodide (PI) staining (Figure 1A). Our screen successfully identified several known ferroptosis inhibitors, as well as a series of L‐type calcium channel blockers, including benidipine, xinidipine, lacidipine, nisoldipine, and manidipine. These compounds significantly suppressed erastin‐induced lipid peroxidation and ferroptosis in both HT1080 fibrosarcoma cells (Figure 1B), MDA‐MB‐231 human breast cancer cells (Figure S1A) and human cardiomyocyte AC16 cells (Figure S1B). Further validation demonstrated that these L‐type calcium channel blockers also inhibited RSL3‐induced lipid peroxidation and ferroptosis in HT1080 (Figure 1C), MDA‐MB‐231 cells (Figure S1A), and AC16 cells (Figure S1B). Compared to antioxidant ferroptosis inhibitor ferrostatin ‐1 and Trolox, these L‐type calcium channel blockers have very little antioxidant activity measured by the DPPH assay (Figure S1C). Treatment with these L‐type calcium channel blockers individually significantly block calcium influx as shown by Fluo‐4 AM staining (Figure S1D). We also accessed the distribution of cellular calcium upon L‐type calcium channel blockers and erastin treatment. Mitochondria and endoplasmic reticulum are the major storage compartments of cellular calcium. As shown in Figure S1E,F, erastin treatment increases the calcium signal in both mitochondria and endoplasmic reticulum, and L‐type calcium channel blockers decrease the calcium signal in both mitochondria and endoplasmic reticulum. In contrast to L‐type calcium channel blockers, activation of L‐type calcium channels using the specific small‐molecule activator Bay K8644 sensitized cells to lipid ROS accumulation and ferroptosis in HT1080 and MDA‐MB‐231 cells (Figure 1D). There are four orthologs of L‐type calcium channels, and overexpression of each individual channel was found to promote calcium influx (Figure S1G) and ferroptosis (Figure 1E).

Given the primary role of L‐type calcium channels in facilitating calcium influx from the extracellular environment, we first tested the requirement of calcium in ferroptosis. As shown in Figure 1F, RSL3, erastin or cystine starvation cannot induce ferroptosis in calcium free medium, and calcium is required for ferroptosis in a dose dependent manner. We further investigated whether calcium chelation could inhibit ferroptosis. As shown in Figure 1G and Figure S1H–J, calcium chelators, including EDTA‐AM and EGTA‐AM, significantly suppressed lipid peroxidation and ferroptosis induced by RSL3, erastin or cystine starvation treatment in multiple cell lines. BHQ, another small molecule blocking plasma membrane Ca^2+^ influx by inhibiting L‐type Ca^2+^ channel [32], could also suppress lipid peroxidation and ferroptosis (Figure 1G; Figure S1H–J). EDTA‐AM and EGTA‐AM have very little antioxidant activity, but BHQ has similar antioxidant activity as ferrostatin ‐1 and Trolox (Figure S1K). Beside to L‐type calcium channels, the P/Q, N, R‐type ion channels and receptor‐gated channels are also common calcium channels. We measured the RNA level of these calcium channels and found that only L‐type calcium channel CaV1.4 and T‐type calcium channel CaV3.2 significantly express in HT1080 cells and MDA‐MB‐231 cells (Figure S1L). Interestingly, treatment with T‐type calcium channels inhibitor ML218 cannot block RSL3 or erastin induced lipid peroxidation and ferroptosis (Figure S1M), suggesting L‐type calcium channels play major roles in ferroptosis in the tested cell lines. These findings collectively demonstrate that calcium influx is a critical requirement for ferroptosis execution.

### 2.2 Calcium‐Dependent PKCβ Activation is Required for Ferroptosis

To elucidate the molecular mechanisms by which calcium regulates ferroptosis, we first compared the expression level of several well‐characterized ferroptosis regulators between control conditions and treatments with L‐type calcium channel blockers or calcium chelators. As shown in Figure S2A, no significant differences in the expression of these proteins were observed, suggesting that calcium's role in ferroptosis is not mediated through modulation of these proteins.

Calcium is a critical signaling molecule involved in numerous biological processes, often acting through downstream pathways such as calpain and calmodulin [33, 34]. However, we found that the pan‐calpain inhibitor calpeptin failed to block ferroptosis (Figure S2B). Similarly, the calmodulin antagonist W‐7 hydrochloride did not inhibit erastin‐induced ferroptosis (Figure S2C), indicating that these pathways are not essential for calcium's role in ferroptosis.

**FIGURE 2:**
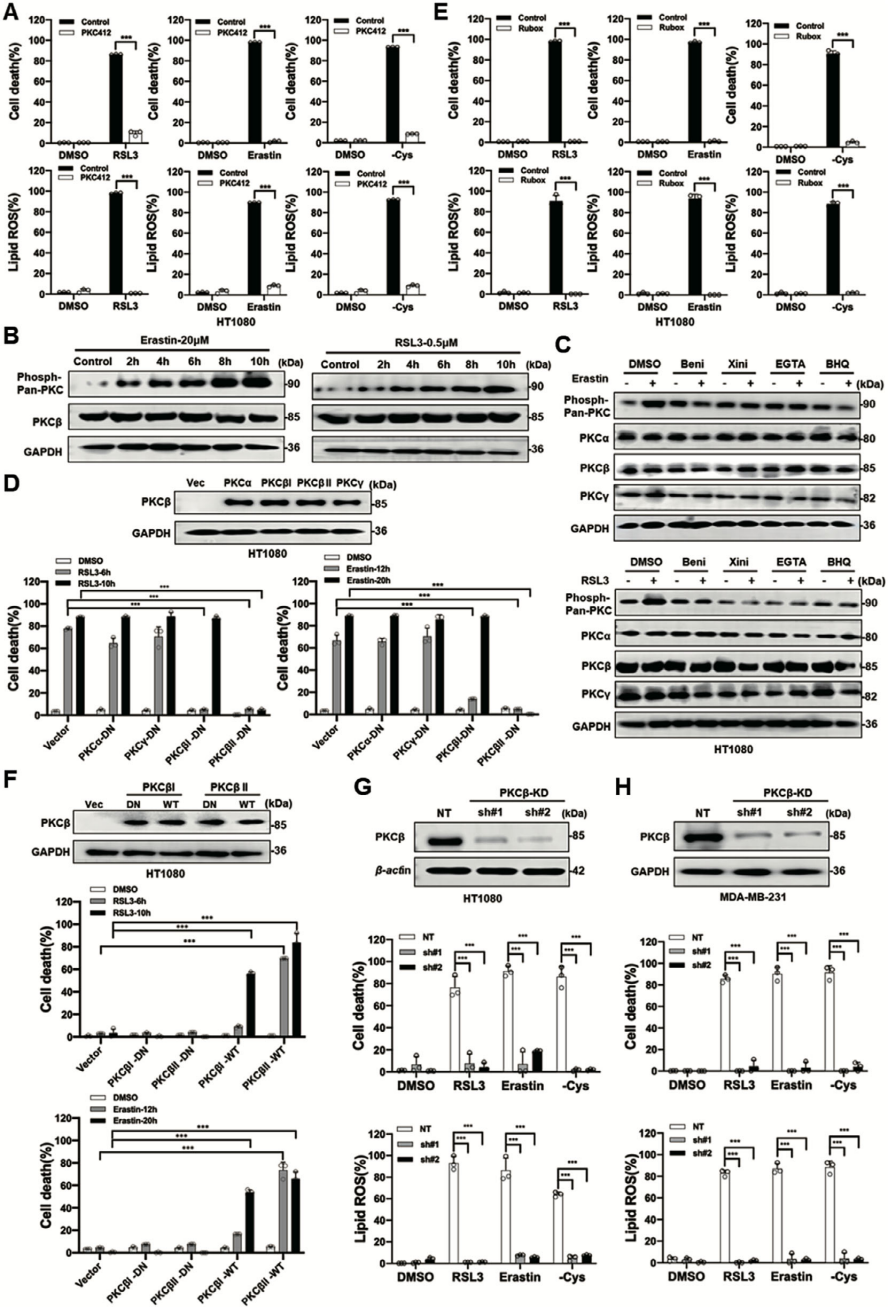
Calcium dependent PKCβ activation is required for ferroptosis A) Pan PKC inhibitor PKC412 treatment blocks ferroptosis and lipid peroxidation. HT1080 cells were treated with RSL3 (1 µM, for 10 h), erastin (10 µM, for 20 h), or cystine starvation (for 30 h) with or without PKC412 (5 µM), then cell death and lipid ROS were measured as described in Figure 1B. B) Erastin or RSL3 induced time‐dependent phosphorylation of PKC in HT1080 cells. Western blot images confirm the expression of the indicated proteins. Erastin (10 µM), RSL3 (1 µM). C) L‐type calcium channel blockers or calcium chelators treatment suppress erastin (10 µM, for 8 h) or RSL3 (1 µM, for 6 h) induced phosphorylation of cPKC without changing their protein level. HT1080 cells were treated with indicated compounds. Western blot images confirmed the expression of the indicated proteins. Erastin (10 µM), Beni: Benidipine (50 µM) Xini: Xinidipine (20 µM), EGTA‐AM (50 µM), BHQ (50 µM). D) Overexpression of dominant‐negative mutant of PKCβI or PKCβII but not PKCα or PKCγ suppress ferroptosis induced by RSL3 or erastin. Western blot images confirmed the expression of the indicated proteins. Indicated HT1080 stable cells were treated with RSL3 (1 µM, for 10 h) or eastin (10 µM, for 20 h) as indicated, then then cell death was measured as described in Figure 1B. E) PKCβ inhibitor ruboxistaurin treatment blocked RSL3 (1 µM, for 10 h), erastin (10 µM, for 20 h) or cystine starvation (for 30 h) induced ferroptosis and lipid peroxidation in MDA‐MB‐231 cells. Rubox: ruboxistaurin (5 µM). F) Overexpression of wildtype but not dominant‐negative mutant of PKCβI or PKCβII promoted ferroptosis induced by RSL3 or erastin. Indicated HT1080 stable cells were treated with RSL3 (1 µM) or eastin (10 µM) as indicated, then cell death was measured as described in Figure 1B. G,H) Knockdown of PKCβ suppressed ferroptosis and lipid peroxidation. Indicated HT1080 (G) or MDA‐MB‐231 (H) stable cells were treated with RSL3 (1 µM) or eastin (10 µM) as indicated, then cell death and lipid ROS were measured as described in Figure 1B. Data are derived from three independent experiments, and each value represents the mean ± SD. ^*^ *p* < 0.05, ^**^ *p* < 0.01, ^***^ *p* < 0.001, t test. (*n* = 3).

We then investigated the involvement of the classical protein kinase C (cPKC) family, which is known to be activated by calcium [35]. Treatment with the pan‐cPKC inhibitor PKC412 (midostaurin) significantly suppressed ferroptosis induced by RSL3, erastin or cystine starvation in HT1080 cell (Figure 2A) and MDA‐MB‐231 cells (Figure S2D). Furthermore, cPKC activation was observed upon ferroptosis induction (Figure 2B), and this activation was inhibited by L‐type calcium channel blockers or calcium chelators (Figure 2C). L‐type calcium channel blockers or calcium chelators have little effect on the expression of cPKCs (Figure 2C).

The cPKC family comprises four members: PKCα, PKCβI, PKCβII, and PKCγ. To identify which cPKC isoform(s) are involved in ferroptosis, we overexpressed the dominant‐negative (DN) mutant of each isoform in wild‐type HT1080 cells and assessed the ferroptosis sensitivity. Overexpression of DN mutants of PKCβI or PKCβII, but not PKCα or PKCγ, significantly suppressed ferroptosis induced by RSL3 or erastin (Figure 2D). Consistent with these findings, the PKCβ activator Ingenol‐5,20‐acetonide‐3‐O‐angelate promoted lipid ROS accumulation and ferroptosis induced by erastin or RSL3 in HT1080 (Figure S2E), and the PKCβ‐specific inhibitor ruboxistaurin inhibited lipid ROS accumulation and ferroptosis induced by cysteine starvation, erastin or RSL3 in both HT1080 and MDA‐MB‐231 cells (Figure 2E; Figure S2F), and overexpression of wild‐type (WT) PKCβI or PKCβII, but not their DN mutants, promoted ferroptosis, with PKCβII exhibiting a stronger pro‐ferroptotic effect compared to PKCβI (Figure 2F). To further validate these findings, we generated PKCβ knockdown (KD) in multiple cell lines using shRNAs targeting both PKCβI and PKCβII. PKCβ knockdown significantly suppressed lipid peroxidation and ferroptosis induced by cystine starvation, erastin, or RSL3 in multiple cells (Figure 2G,H; Figure S2G,H). Additionally, PKCβ knockdown or inhibition of PKCβ with ruboxistaurin abrogated ferroptosis even in the presence of the calcium channel activator Bay K8644 (Figure S2I,J). These results collectively demonstrate that PKCβ (both PKCβI and PKCβII) play a critical role in positively regulating ferroptosis downstream of calcium signaling.

### 2.3 PKCβ Forms a Complex with ACSL4 and ALOX15

To investigate how PKCβ regulates ferroptosis, we first compared the expression levels of several well‐studied ferroptosis regulators between control cells and PKCβ knockdown (KD) cells. As shown in Figure S3A, no significant differences in the protein levels of these ferroptosis regulators were observed, suggesting that PKCβ’s role in ferroptosis is not mediated through transcriptional or translational modulation of these proteins.

**FIGURE 3:**
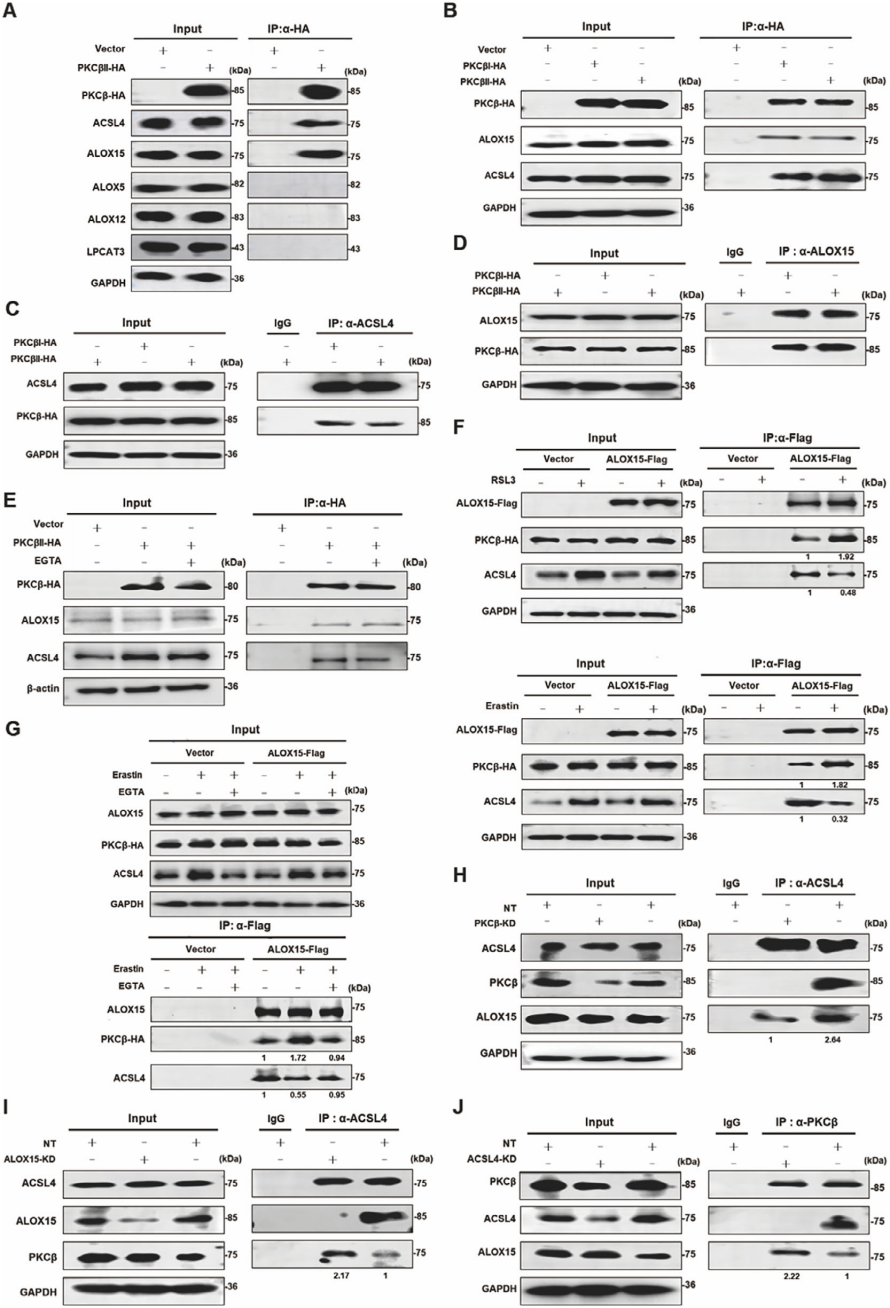
PKCβ forms a complex with ACSL4 and ALOX15. A) PKCβ interacts with ACSL4 and ALOX15 but no other ferroptosis positive regulators. The IP assay was performed using an antibody against HA tag using indicated cell lysate. Western blot images confirmed the expression of the indicated proteins. B) Both PKCβI and PKCβII interact with ACSL4 and ALOX15. The IP assay was performed using an antibody against HA tag using indicated cell lysate. Western blot images confirm the expression of the indicated proteins. C) Both PKCβI and PKCβII interact with ACSL4 and ALOX15. The IP assay was performed using an antibody against ACSL4 or IgG control using indicated cell lysates. Western blot images confirmed the expression of the indicated proteins. D) Both PKCβI and PKCβII interact with ACSL4 and ALOX15. The IP assay was performed using an antibody against ALOX15 or IgG control using indicated cell lysate. Western blot images confirmed the expression of the indicated proteins. E) EGTA‐AM (50 µM for 8 h) does not affect the formation of ACSL4‐PKCβ‐ALOX15 complex. The IP assay was performed using an antibody against HA tag using indicated cell lysate. Western blot images confirmed the expression of the indicated proteins. F) RSL3 or erastin treatment enhanced the interaction between ALOX15 and PKCβ but impaired the interaction between ALOX15 and ACSL4. The IP assay was performed using an antibody against FLAG tag using indicated MDA‐MB‐231 cell lysate. Western blot images confirmed the expression of the indicated proteins. RSL3 (1 µM for 6 h), erastin (20 µM for 8 h). G) EGTA treatment blocked the effect of the erastin on the formation of ACSL4‐PKCβ‐ALOX15 complex. The IP assay was performed using an antibody against FLAG tag using indicated cell lysate. Western blot images confirmed the expression of the indicated proteins in MDA‐MB‐231 cells. erastin (20 µM for 8 h), EGTA (50 µM for 8 h). H) Knockdown of PKCβ impaired the interaction between ALOX15 and ACSL4. The IP assay was performed using an antibody against ACSL4 or IgG control using indicated cell lysate. Western blot images confirmed the expression of the indicated proteins. I) Knockdown of ALOX15 enhances the interaction between ACSL4 and PKCβ. The IP assay was performed using an antibody against ACSL4 or IgG control using indicated cell lysate. Western blot images confirmed the expression of the indicated proteins. J) Knockdown of ACSL4 enhances the interaction between ALOX15 and PKCβ. The IP assay was performed using an antibody against PKCβ or IgG control using indicated cell lysate. Western blot images confirmed the expression of the indicated proteins.

We hypothesized that PKCβ might directly interact with key ferroptosis positive regulators. To test this, we performed co‐immunoprecipitation (Co‐IP) assays and revealed that PKCβ interacts with ACSL4 and ALOX15 but no other ferroptosis positive regulators (Figure 3A). Subsequent Co‐IP experiments confirmed that both PKCβI and PKCβII form a protein complex with ACSL4 and ALOX15 (Figure 3B–D; Figure S3B). Interestingly, while calcium is essential for ferroptosis, depletion of cellular calcium using EGTA‐AM did not disrupt the formation of the ACSL4‐PKCβ‐ALOX15 ternary complex (Figure 3E). We further observed that treatment with erastin or RSL3 enhanced the interaction between ALOX15 and PKCβ but weakened the interaction between ALOX15 and ACSL4 (Figure 3F). Furthermore, erastin induced enhancement of the interaction between ALOX15 and PKCβ and erastin induced suppression of the interaction between ALOX15 and ACSL4 could be blocked by EGTA treatment (Figure 3G), highlighting the crucial role of calcium in ferroptosis.

ALOX15 contains two functional domains: the N terminal PLAT domain and the C terminal Lipoxygenase domain. We found that ALOX15 interacts with PKCβ through C terminal Lipoxygenase domain (Figure S3C). And the C terminal Lipoxygenase domain is sufficient for ferroptosis (Figure S3D,E).

PKCβ contains three functional domains. In the N terminal, there are two conserved domains (C1 domain and C2 domain) and the C terminal is a kinase domain. Which domain(s) mediate its interaction with ACSL4 or ALOX15? We found that the C1 domain and C2 domain of PKCβ interact with ACSL4 strongly, but the kinase domain of PKCβ interacts with ACSL4 weakly. The C2 domain and kinase domain of PKCβ interact with ALOX15 strongly, but the C1 domain of PKCβ could not interact with ALOX15 (Figure S3F).

We also investigated the role of each protein in maintaining the integrity of the ACSL4‐PKCβ‐ALOX15 complex. Knockdown of PKCβ disrupted the interaction between ACSL4 and ALOX15 (Figure 3H), while knockdown of ALOX15 enhanced the interaction between ACSL4 and PKCβ (Figure 3I). Similarly, knockdown of ACSL4 strengthened the interaction between ALOX15 and PKCβ (Figure 3J). These findings suggest that PKCβ occupies a central position within the ACSL4‐PKCβ‐ALOX15 complex, acting as a scaffold that facilitates the interaction between ACSL4 and ALOX15. These results collectively demonstrate that PKCβ forms a functional complex with ACSL4 and ALOX15, providing a mechanistic link between calcium signaling, lipid metabolism, and ferroptosis execution.

### 2.4 The Thr^484th^ of ACSL4, a Novel Phosphorylated Site by PKCβ, is Required for Ferroptosis

**FIGURE 4:**
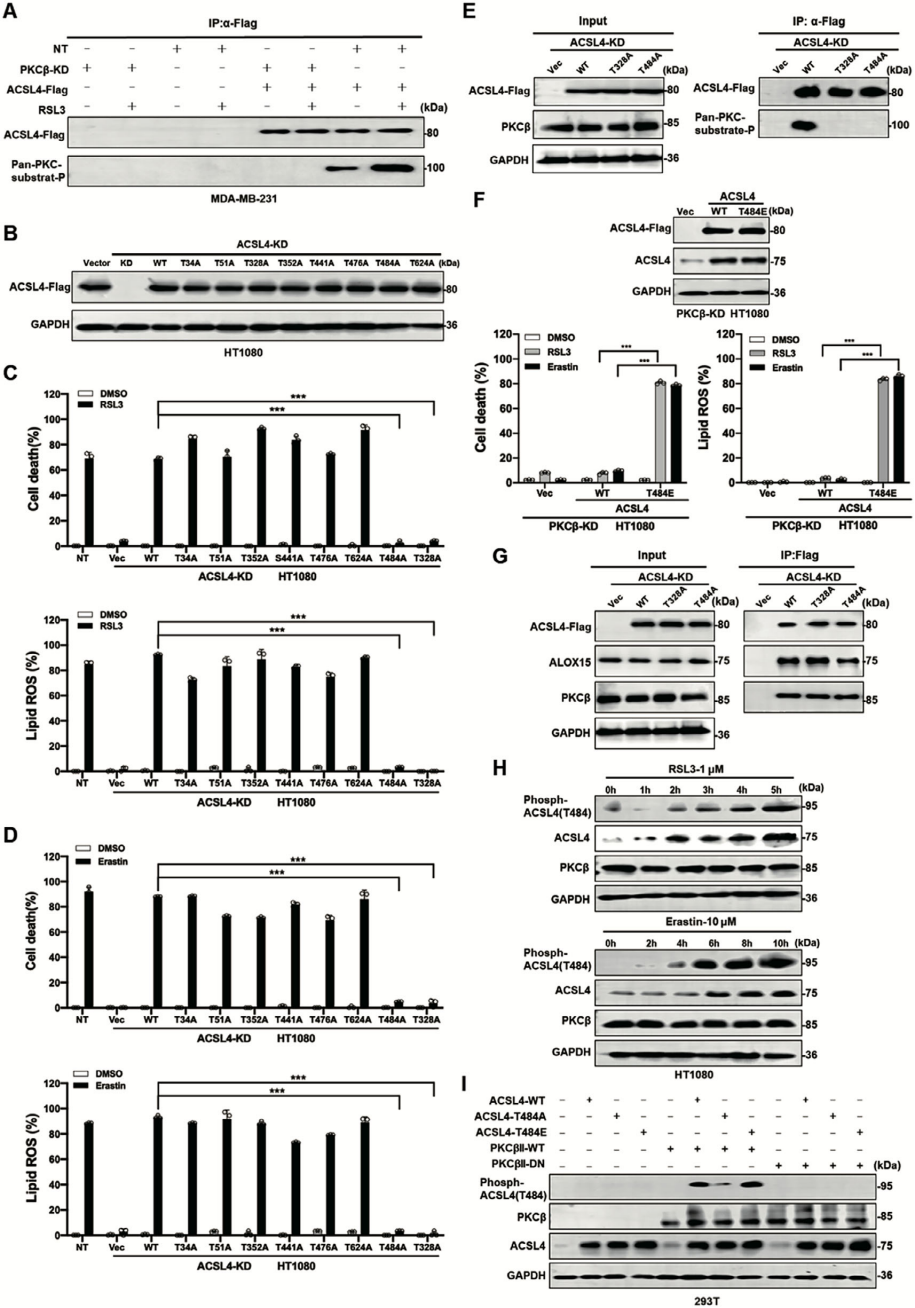
The Thr^484th^ of ACSL4, a novel phosphorylated site by PKCβ, is required for ferroptosis. A) RSL3 treatment enhances the phosphorylation of ACSL4 by PKCβ. The IP assay was performed using an antibody against FLAG using indicated cell lysate. Western blot images confirmed the expression of the indicated proteins. RSL3 (1 µM for 8 h). B) Reconstitution of individual ACSL4 mutant back to ACSL4 KD HT1080 cells. Western blot images confirmed the expression of the indicated proteins. C) ACSL4 with T328A or T484A loss of function mutant could not rescue RSL3 induced ferroptosis and lipid peroxidation. ACSL4 KD HT1080 cells reconstituted with individual ACSL4 mutant as indicated were treated with RSL3 (1 µM, for 10 h), then cell death and lipid ROS were measured as described in Figure 1B. D) ACSL4 with T328A or T484A loss of function mutant could not rescue erastin induced ferroptosis and lipid peroxidation. ACSL4 KD HT1080 cells reconstituted with individual ACSL4 mutant as indicated were treated with erastin (10 µM) for 20 h, then cell death and lipid ROS were measured as described in Figure 1B. E) ACSL4 with T328A or T484A mutant cannot be phosphorylated by PKCβ. The IP assay was performed using an antibody against FLAG using indicated cell lysates. Western blot images confirm the expression of the indicated proteins. F) Overexpression of ACSL4 with T484E phosphorylation mimic mutant promotes ferroptosis and lipid peroxidation in PKCβ KD HT1080 cells. PKCβ KD HT1080 cells overexpressed with ACSL4^T484E^ phosphorylation mimic mutant were treated with RSL3 (1 µM for 10 h) or erastin (10 µM, for 20 h), then cell death and lipid ROS were measured as described in Figure 1B. G) The effect of ACSL4 loss of function mutants on the formation of ACSL4‐PKCβ‐ALOX15 complex. The IP assay was performed using an antibody against FLAG using indicated cell lysates. Western blot images confirmed the expression of the indicated proteins. H) Time‐dependent phosphorylation of ACSL4 at T484 detected by the antibody against p‐ACSL4^T484^. HT1080 cells were treated with RSL3 (1 µM) or erastin (10 µM) as indicated. Western blot images confirmed the expression of the indicated proteins. I) Phosphorylation of ACSL4 at T484 by wildtype PKCβII but not PKCβII dominant‐negative mutant. 293T cells with transient expression of indicated proteins. Western blot images confirmed the expression of the indicated proteins. Data are derived from three independent experiments, and each value represents the mean ± SD. ^*^ *p* < 0.05, ^**^ *p* < 0.01, ^***^ *p* < 0.001, *t* test. (*n* = 3).

Given the interaction between PKCβ and ACSL4, we hypothesized that ACSL4 might be a substrate of PKCβ. To test this, FLAG‐tagged ACSL4 was expressed in either PKCβ wild‐type (WT) or PKCβ knockdown (KD) cells. ACSL4 was immunoprecipitated using an anti‐FLAG antibody and probed with a phospho‐PKC substrate antibody. We observed that ACSL4 was phosphorylated in PKCβ WT cells but not in PKCβ KD cells, and RSL3 treatment further enhanced ACSL4 phosphorylation (Figure 4A), confirming that ACSL4 is a substrate of PKCβ.

To identify the specific phosphorylation sites on ACSL4, we performed immunoprecipitation followed by mass spectrometry. However, no phosphorylation sites were detected (data not shown). We then used bioinformatics tools to predict potential PKCβ phosphorylation sites on ACSL4. These predicted serine/threonine residues were mutated to alanine, and the mutants were individually expressed in ACSL4 KD cells to assess their impact on ferroptosis sensitivity (Figure 4B). The ACSL4^T328A^ and ACSL4^T484A^ mutants failed to restore ferroptosis sensitivity (Figure 4C,D), indicating that Thr^328^ and Thr^484^ are critical for ACSL4 function. Immunoprecipitation experiments confirmed that both Thr^328^ and Thr^484^ are phosphorylated by PKCβ, as only WT ACSL4, and not the T328A or T484A mutants, was detected by the phospho‐PKC substrate antibody (Figure 4E). While phosphorylation of Thr^328^ by PKCβ has been previously reported[36], we focused on the novel Thr^484^ site. Expression of the phosphomimetic ACSL4^T484E^ mutant in PKCβ KD HT1080 cells or MDA‐MB‐231 cells not only rescued but also enhanced ferroptosis sensitivity ferroptosis sensitivity (Figure 4F; Figure S4A,B). Furthermore, the phosphomimetic ACSL4^T484E^ mutant has higher activity compared to wildtype ACSL4 (Figure S4C). Interestingly, Thr^484^ is essential for ferroptosis, but did not impair the interaction between ACSL4 and PKCβ (Figure 4G).

We generated a phosphorylation‐specific antibody against ACSL4^T484^ (p‐ACSL4^T484^) and found that RSL3 or erastin treatment induced time‐dependent phosphorylation of ACSL4^T484^ (Figure 4H). Co‐expression of WT PKCβ, but not dominant‐negative (DN) mutant, with WT ACSL4 or the ACSL4^T484E^ mutant, but not the ACSL4^T484A^ mutant, confirmed the specificity of the p‐ACSL4^T484^ antibody (Figure 4I). The in vitro kinase assay further confirmed that PKCβ directly phosphorylates wildtype ACSL4 but not ACSL4^T484A^ mutant (Figure S4D). Importantly, phosphorylation of ACSL4^T484^ was detected exclusively during ferroptosis and not in necroptosis or pyroptosis, two well studied regulated necrosis (Figure S4E), establishing p‐ACSL4^T484^ as a specific biomarker for ferroptosis.

### 2.5 Phosphorylation of Multiple Ser/Thr Sites on ALOX15 by PKCβ is Required for Ferroptosis

**FIGURE 5:**
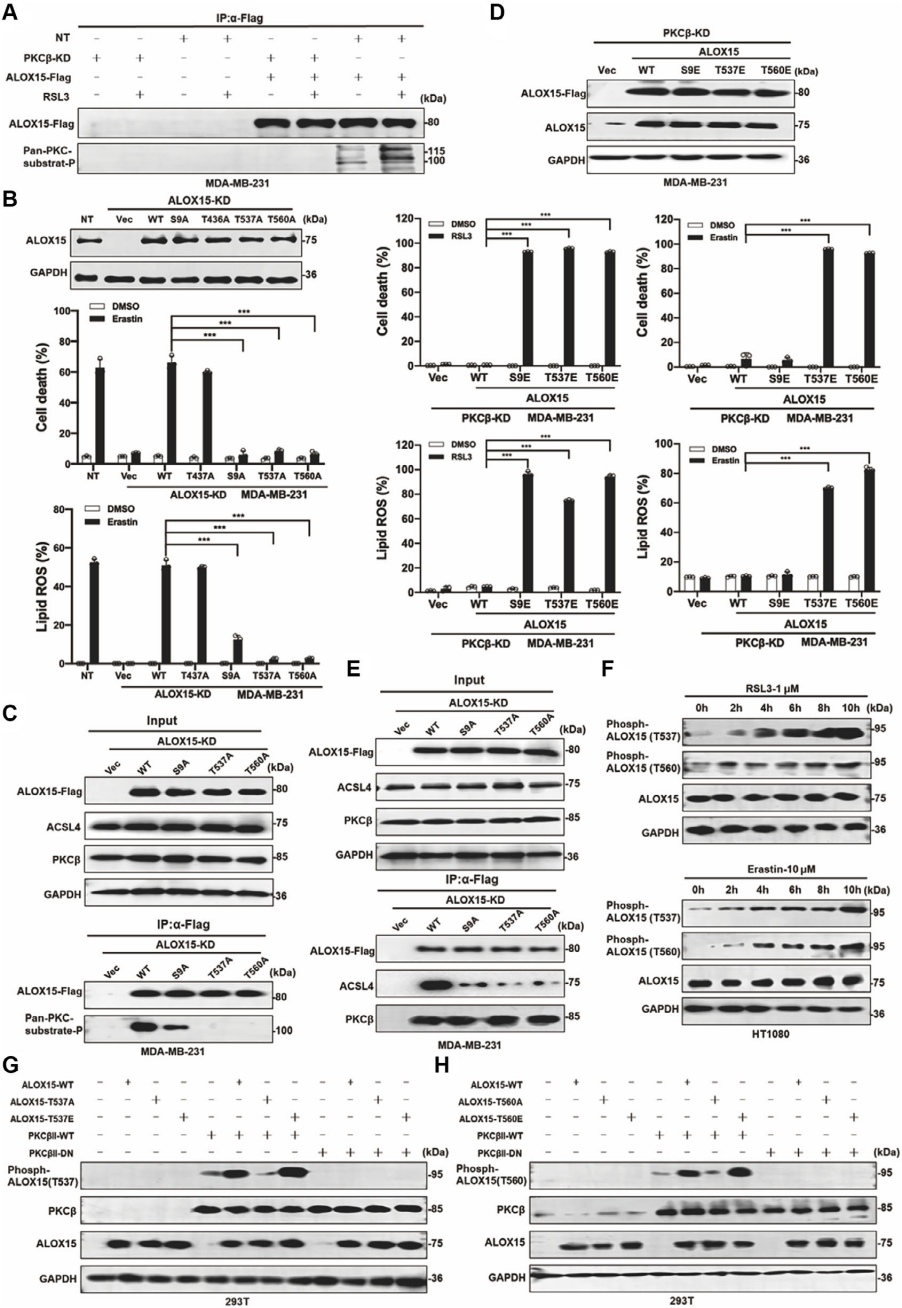
Phosphorylation of multiple Ser/Thr sites of ALOX15 by PKCβ are required for ferroptosis. a) RSL3 (1 µM for 8 h) treatment enhances the phosphorylation of ALOX15 by PKCβ. The IP assay was performed using an antibody against FLAG using indicated cell lysate. Western blot images confirmed the expression of the indicated proteins. b) ALOX15 with S9A, T537A, or T560A loss of function mutant could not rescue erastin (20 µM, for 20 h) induced ferroptosis and lipid peroxidation in ALOX15 KD MDA‐MB‐231 cells. Reconstitution of individual ALOX15 mutant back to ALOX15 KD MDA‐MB‐231 cells. Western blot images confirmed the expression of the indicated proteins. c) Phosphorylation of ALOX15 by PKCβ is impaired by ALOX15^S9A^, ALOX15^T537A^, or ALOX15^T560A^ mutation. The IP assay was performed using an antibody against FLAG using indicated cell lysate. Western blot images confirmed the expression of the indicated proteins. d) Overexpression of ALOX15 with S9E, T537E or T560E phosphorylation mimic mutants promote ferroptosis and lipid peroxidation in PKCβ KD MDA‐MB‐231 cells. Western blot images confirmed the expression of the indicated proteins. PKCβ KD MDA‐MB‐231 cells as indicated were treated with RSL3 (0.5 µM, for 10 h) or erastin (20 µM, for 20 h), then cell death and lipid ROS were measured as described in Figure 1b. e) ALOX15 S9A, T537A or T560A loss of function mutant impaired the interaction between ACSL4 and ALOX15, but did not change the interaction between ALOX15 and PKCβ. The IP assay was performed using an antibody against FLAG using indicated cell lysate. Western blot images confirmed the expression of the indicated proteins. f) Time‐dependent phosphorylation of ALOX15 at T537 and T560 detected by the antibodies against p‐ALOX15^T537^ or p‐ALOX15^T60^. HT1080 cells were treated with RSL3 (1 µM) or erastin (10 µM) as indicated. Western blot images confirmed the expression of the indicated proteins. g) Phosphorylation of ALOX15 at T537 by wildtype PKCβII but not PKCβII dominant‐negative mutant. 293T cells with transient expression of indicated proteins. Western blot images confirmed the expression of the indicated proteins. h) Phosphorylation of ALOX15 at T560 by wildtype PKCβII but not PKCβII dominant‐negative mutant. 293T cells with transient expression of indicated proteins. Western blot images confirmed the expression of the indicated proteins. Data are derived from three independent experiments, and each value represents the mean ± SD. ^*^ *p* < 0.05, ^**^ *p* < 0.01, ^***^ *p* < 0.001, *t* test. (*n* = 3).

Similar to ACSL4, we hypothesized that ALOX15 is another substrate of PKCβ. FLAG‐tagged ALOX15 was expressed in PKCβ WT or KD cells, immunoprecipitated, and probed with a phospho‐PKC substrate antibody. ALOX15 phosphorylation was observed in PKCβ WT cells but not in PKCβ KD cells, and RSL3 treatment enhanced this phosphorylation (Figure 5A), confirming ALOX15 as a PKCβ substrate.

Although mass spectrometry failed to identify specific phosphorylation sites, bioinformatics analysis predicted potential PKCβ phosphorylation sites on ALOX15 (Figure 5B). Mutation of these serine/threonine residues to alanine revealed that ALOX15^S9A^, ALOX15^T537A^, and ALOX15^T560A^ mutants could not restore ferroptosis sensitivity in ALOX15 KD cells (Figure 5B), indicating that Ser^9^, Thr^537^, and Thr^560^ are essential for ALOX15 function. Immunoprecipitation confirmed that these sites are phosphorylated by PKCβ, as the ALOX15^T537A^ and ALOX15^T560A^ mutants were not detected by the phospho‐PKC substrate antibody, and the signal for ALOX15^S9A^ was significantly reduced (Figure 5C). Expression of phosphomimetic mutants (ALOX15^T537E^, and ALOX15^T560E^) in PKCβ KD cells not only rescued but also enhanced ferroptosis sensitivity (Figure 5D; Figure S5A–C). Furthermore, the phosphomimetic mutants (ALOX15^T537E^, and ALOX15^T560E^) have higher activity compared to wildtype ALOX15 (Figure S5D). Interestingly, expression phosphomimetic mutant of ALOX15^S9E^ in PKCβ KD cells rescued and enhanced RSL3 induced ferroptosis but not erastin induced ferroptosis (Figure 5D; Figure S5A–C). While these mutants retained their interaction with PKCβ, their binding to ACSL4 was significantly impaired (Figure 5E).

We generated phosphorylation‐specific antibodies against p‐ALOX15^T537^ and p‐ALOX15^T560^. RSL3 or erastin treatment induced time‐dependent phosphorylation of these sites (Figure 5F). Co‐expression of WT PKCβ, but not PKCβ DN mutant, with WT ALOX15 or the phosphomimetic mutants confirmed the specificity of these antibodies (Figure 5G,H). The in vitro kinase assay further confirmed that PKCβ directly phosphorylates wildtype ALOX15 but not ALOX15^T537A^ mutant or ALOX15^T560A^ (Figure S5E,F). Similar to ACSL4^T484^, phosphorylation of ALOX15^T537^ and ALOX15^T560^ was specific to ferroptosis and not observed in necroptosis and pyoptosis, two forms of regulated necrosis (Figure S5G), establishing these phosphorylation (p‐ALOX15^T537^ and p‐ALOX15^T560^) as additional biomarkers for ferroptosis.

### 2.6 The PKCβ‐ACSL4‐ALOX15 Complex Translocate to Lipid Droplet During Ferroptosis

**FIGURE 6:**
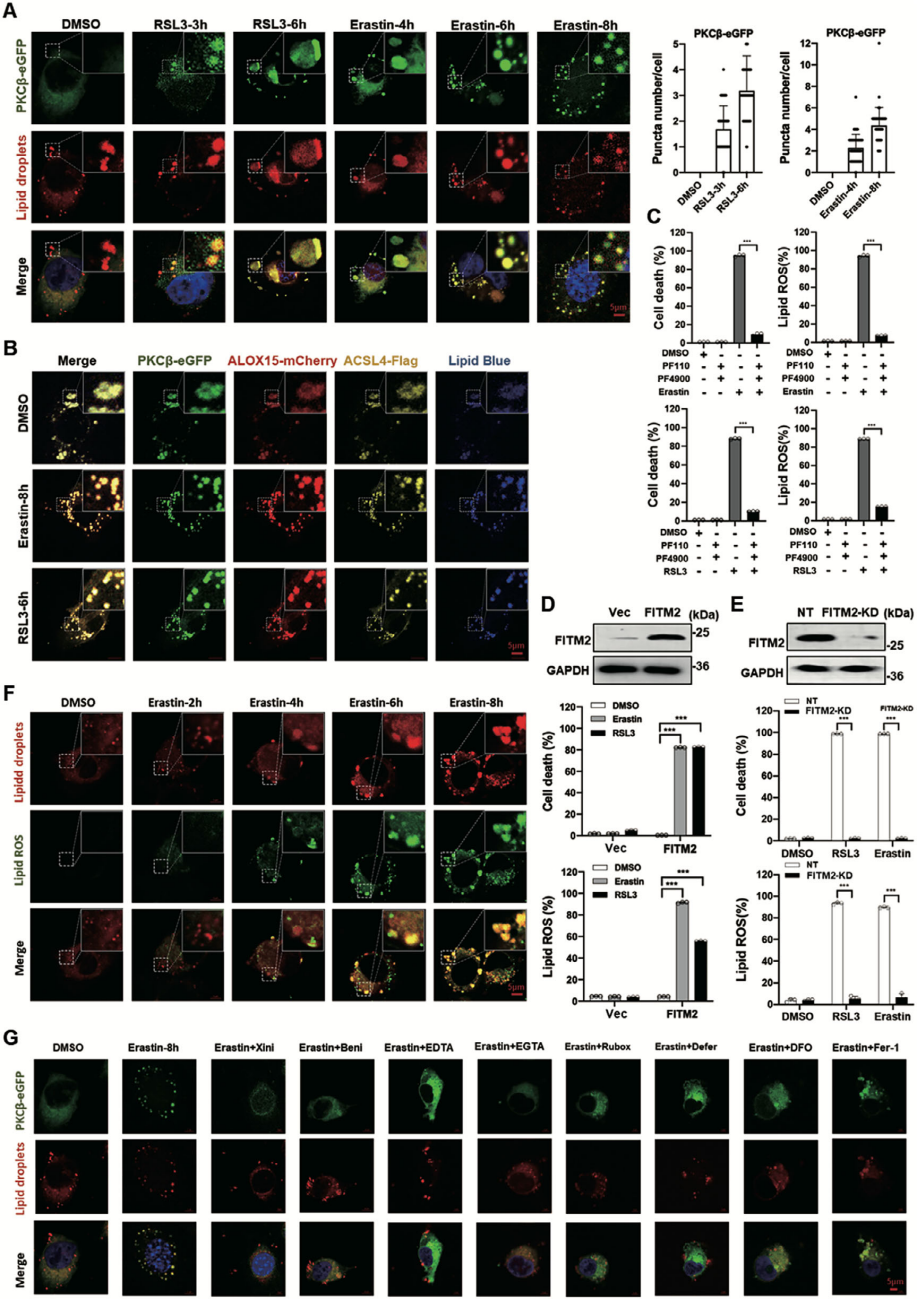
The ACSL4‐PKCβ‐ALOX15 complex translocate to lipid droplets during ferroptosis. A) RSL3 or erastin treatment induces puncta formation of PKCβ and translocation of PKCβ puncta to lipid droplets. PKCβ‐eGFP HT1080 cells were treated with RSL3 (1 µM) or erastin (10 µM) as indicated, then stained with lipid droplets dye Nile Red, and imaged with Confocal Microscopy. Quantitative bar shows the average puncta number per cell, 50 cells were counted. b, RSL3 or erastin treatment induces puncta formation of PKCβ‐ACSL4‐ALOX15 complex and its translocation to lipid droplets. HT1080 cells co‐expressed with PKCβ‐eGFP, ALOX15‐mCherry, and ACSL4‐FLAG were treated with RSL3 or erastin as indicated, then stained with lipid droplets dye Lipi‐blue, then imaged with Confocal Microscopy. RSL3 (1 µM), erastin (10 µM). c, Inhibition of DGAT1 and DGAT2 block ferroptosis and lipid peroxidation. HT1080 cells were treated RSL3 (1 µM, for 10 h) or erastin (10 µM, for 20 h) as indicated, then cell death and lipid ROS were measured as described in Figure 1B. PF110 (20 µM), PF4900 (40 µM). d, Overexpression of FITM2 promotes ferroptosis and lipid peroxidation. HT1080 cells as indicated were treated with RSL3 (1 µM, for 10 h) or erastin (10 µM, for 16 h), then cell death and lipid ROS were measured as described in Figure 1B. e, Knockdown of FITM2 suppressed ferroptosis and lipid peroxidation. HT1080 cells as indicated were treated with RSL3 (1 µM, for 10 h) or erastin (10 µM, for 16 h), then cell death and lipid ROS were measured as described in Figure 1B. F) Colocalization of erastin induced lipid ROS and lipid droplets. HT1080 cells were treated with erastin (10 µµ) as indicated, cells were stained with Nile Red for lipid droplets and Liperfluo for lipid ROS, then imaged with Confocal Microscopy. G) The role of ferroptosis inhibitors on erastin induced PKC puncta formation and its lipid droplets translocation. PKCβ‐eGFP HT1080 cells were treated as indicated, then stained with Nile Red for lipid droplets, then imaged with Confocal Microscopy. Xini: xinidipine (20 µM), Beni: benidipine (50 µM), EDTA‐AM: 40 µM, EGTA‐AM: 50 µM, Rubox: ruboxistaurin (5 µM), Defer: 50 µM, DFO 100 µM, Fer‐1: 100 µM. Data are derived from three independent experiments, and each value represents the mean ± SD. ^*^ *p* < 0.05, ^**^ *p* < 0.01, ^***^ *p* < 0.001, *t* test. (*n* = 3). Scale bar: 5 µm.

Upon activation, PKCβ is typically recruited to the membrane [35]. Given that ferroptosis stimuli induce PKCβ phosphorylation, we investigated whether these stimuli alter the subcellular localization of PKCβ. We found that PKCβ formed punctate structures in response to erastin or RSL3 treatment in a time‐dependent manner (Figure 6A; Figure S6A). Further analysis revealed that co‐expression of PKCβ with either ACSL4 or ALOX15 results in the constitutive formation of ACSL4‐PKCβ or ALOX15‐PKCβ puncta. And treatment with erastin or RSL3 enhances the formation of these puncta over time (Figure S6B,C). Importantly, we found that the PKCβ puncta co‐localize with lipid droplets, suggesting a potential role of lipid droplets in PKCβ‐mediated ferroptosis signaling (Figure 6A). Similarly, erastin or RSL3 induced time‐dependent ACSL4 puncta or ALOX15 puncta co‐localize with lipid droplets (Figure S6D–G). We further determined that the ACSL4‐PKCβ‐ALOX15 complex constitutively form puncta which co‐localize with lipid droplets, and erastin or RSL3 treatment promotes the formation of these puncta (Figure 6B).

The Lipid droplets, known as central organelles in lipid and energy homeostasis, have a debated role in ferroptosis [2]. To clarify this, we targeted diacylglycerol O‐acyltransferases (DGATs), the rate‐limiting enzymes in triacylglycerol (TAG) synthesis. Inhibition of DGATs by small molecule inhibitors significantly attenuated erastin or RSL3 induced ferroptosis (Figure 6C). Consistently, knockdown of DGAT1 significantly suppressed erastin or RSL3 induced ferroptosis (Figure S6H), while overexpression of DGAT1 promoted erastin or RSL3 induced ferroptosis (Figure S6I). Additionally, we examined the role of Fat Storage Inducing Transmembrane Protein 2 (FITM2), a key regulator of lipid droplet formation. Knockdown of FITM2 conferred resistance to ferroptosis, while its overexpression enhanced cellular sensitivity to ferroptosis (Figure 6D,E). To further elucidate the relationship between lipid droplets and ferroptosis, we co‐stained cells with the lipid ROS marker Lipofluo and the lipid droplet marker Nile red. We observed that lipid peroxides generated by erastin treatment co‐localized with lipid droplets (Figure 6F), indicating that lipid droplets serve as critical sites for the accumulation of lipid peroxides during ferroptosis. Notably, the interaction enhanced by erastin treatment between PKCβ and ALOX15 was impaired by the DGATs inhibitors treatment, and the interaction decreased by erastin treatment between ACSL4 and ALOX15 was enhanced by the DGATs inhibitors treatment (Figure S6J). The DGATs inhibitors treatment blocked erastin induced PKCβ activation and the phosphorylation of PKCβ downstream substrates ACSL4 and ALOX15 (Figure S6K). These data highlight the crucial role of lipid droplets in ferroptosis regulation.

We next carefully investigated the function of a series of ferroptosis inhibitors on ferroptosis stimuli induced re‐localization of ACSL4‐PKCβ‐ALOX15 complex to lipid droplets. L‐ L‐type calcium channel blockers benidipine and xinidipine, or calcium chelator EGTA‐AM and EDTA treatment blocked the translocation of PKCβ, ACSL4 and ALOX15 to lipid droplets (Figure 6G; Figure S6L,M), suggesting calcium is required for the translocation of PKCβ, ACSL4 and ALOX15 to lipid droplets. PKCβ inhibitor ruboxistaurin treatment blocked the translocation of PKCβ, ACSL4, and ALOX15 to lipid droplets (Figure 6G; Figure S6L,M). Furthermore, ferroptosis inhibitor iron chelator deferiprone (DEP) and DFO blocked the translocation of PKCβ, ACSL4 and ALOX15 to lipid droplets (Figure 6G; Figure S6L,M), suggesting iron is required for the translocation of PKCβ, ACSL4 and ALOX15 to lipid droplets. Interestingly, the classic ferroptosis inhibitor ferrostatin‐1 treatment could not block the translocation of PKCβ, ACSL4, and ALOX15 to lipid droplets (Figure 6G and Figure S6L,M).

Whether loss‐of‐function mutants of ACSL4 or ALOX15 affect their translocation to lipid droplets? We introduced mCherry tagged ACSL4^T484A^ mutant into ACSL4 KD cells. We found that ACSL4^T484A^ does not affect the translocation of ACSL4 to lipid droplets induced by erastin or RSL3 treatment (Figure S6N). Similarly, we introduced mCherry tagged ALOX15^S9A^, ALOX15^T537A^ or ALOX15^T560A^ mutant into ALOX15 KD cells individually. We found that ALOX15^S9A^, ALOX15^T537A^, or ALOX15^T560A^ do not affect the translocation of ALOX15 to lipid droplets (Figure S6O).

Do gain‐of‐function mutants of ACSL4 or ALOX15 affect their translocation to lipid droplets? We introduced mCherry tagged ACSL4^T484E^ mutant into PKCβ KD cells. Interestingly, we found that ACSL4^T484E^ mutant constitutively forms lipid droplet localized puncta in PKCβ KD cells, and there are much more lipid droplet localized ACSL4^T484E^ puncta than the wild‐type ACSL4 puncta induced by erastin, while the size of lipid droplets localized ACSL4^T484E^ puncta is much smaller than the wild‐type ACSL4 puncta induced by erastin (Figure S6P,Q). Similarly, we introduced mCherry tagged ALOX15^S9E^, ALOX15^T537E^ or ALOX15^T560E^ mutant into PKCβ KD cells individually. We found that ALOX15^S9E^, ALOX15^T537E^ or ALOX15^T560E^ mutant constitutively forms lipid droplet localized puncta in PKCβ KD cells, and there are much more lipid droplet localized puncta of ALOX15^S9E^, ALOX15^T537E^ or ALOX15^T560E^ than the wild‐type ALOX15 puncta induced by erastin, while the size of lipid droplet localized puncta of ALOX15^S9E^, ALOX15^T537E^ or ALOX15^T560E^ is much smaller than the wild‐type ALOX15 puncta induced by erastin (Figure S6R,S).

### 2.7 High PKCβ Signaling Promotes Ferroptosis‐Inducing Cancer Therapy

Protein kinase C beta (PKCβ) signaling has been implicated as a critical regulator of tumor initiation and progression [37]. Consistent with its oncogenic role, tumor tissues exhibit significantly higher levels of PKCβ expression compared to adjacent normal tissues [38]. Furthermore, elevated PKCβ expression is associated with poor overall survival in patients across multiple cancer types, underscoring its potential as a prognostic marker and therapeutic target.

**FIGURE 7:**
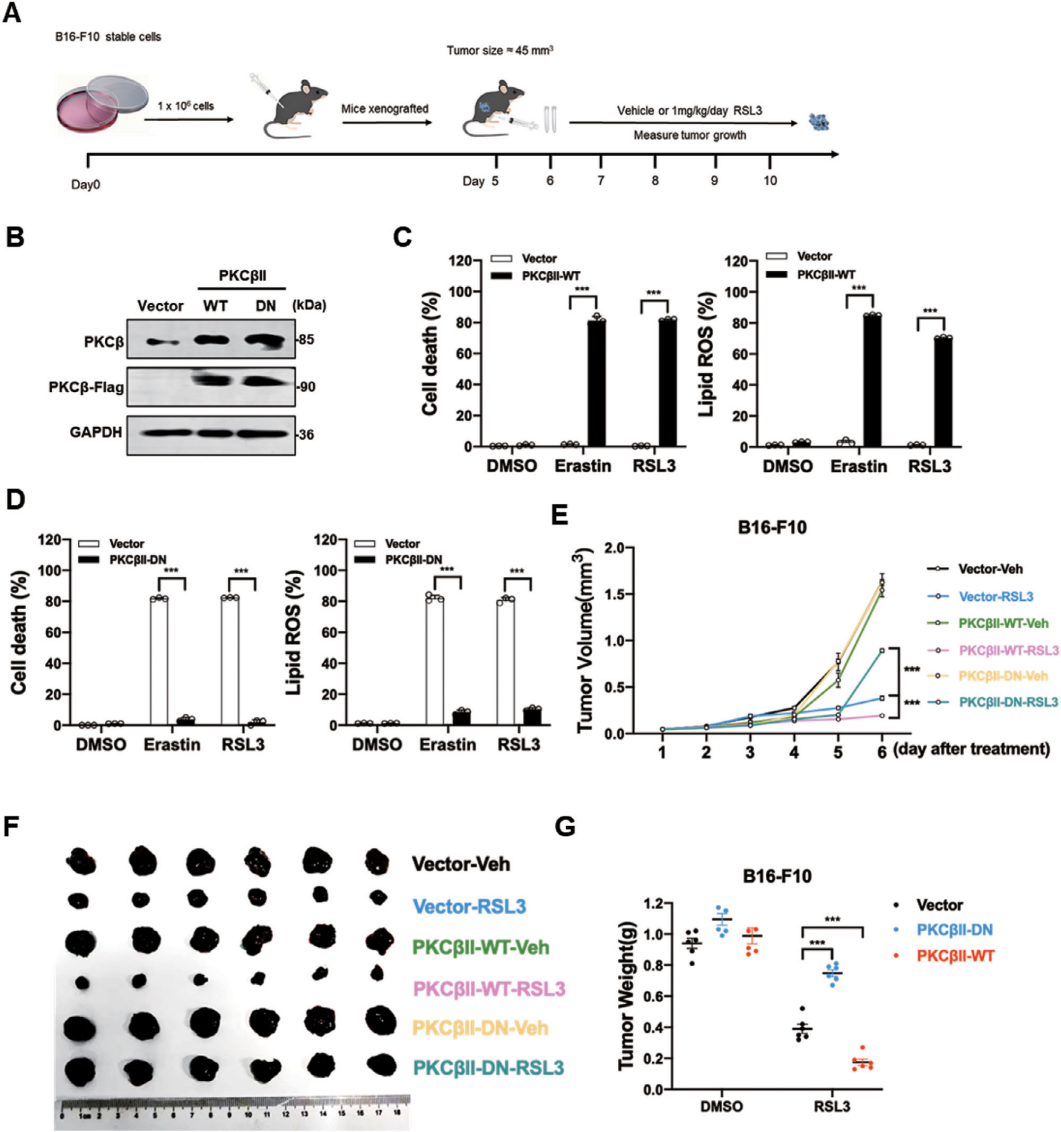
High PKCβ Signaling Promotes Ferroptosis‐inducing Cancer Therapy. A) A schematic diagram shows the procedure of the mouse xenograft model experiment. B) Western blot images confirmed the expression of the indicated proteins. C) Overexpression of PKCβII promotes ferroptosis induced by RSL3 or erastin in B16‐F10 cells. D) Overexpression of dominant‐negative mutant of PKCβII suppress ferroptosis induced by RSL3 or erastin in B16‐F10 cells. B16‐F10 cells were treated with RSL3 (1 µM, for 10 h) or erastin (15 µM, for 20 h) as indicated, then then cell death and lipid peroxidation was measured as described in Figure 1B. E,F) Overexpression of PKCβII sensitizes cancer cells to ferroptosis‐inducing tumor therapy in a mouse xenograft model. Mouse xenograft model experiments with indicated stable B16‐F10 cells were performed as described in Figure 8C. Tumor volume was measured daily. Tumor volume post‐RSL3 (1 mg/ kg) treatment is shown and tumors were photographed and weighted after 6 days RSL3 treatment. G) the working model showing the mechanism that calcium‐dependent phosphorylation and lipid droplets relocation of ACSL4 ‐PKCβ‐ ALOX15 complex dictate ferroptosis. Data are derived from three independent experiments, and each value represents the mean ± SD. ^*^ *p* < 0.05, ^**^ *p* < 0.01, ^***^ *p* < 0.001, *t* test. (*n* = 6).

Given these observations, we investigated whether PKCβ could serve as a predictive biomarker for ferroptosis‐inducing cancer therapies. Using a mouse xenograft model of melanoma (Figure 7A), we evaluated the impact of PKCβ modulation on sensitivity to RSL3 induced ferroptosis. Overexpression of wild‐type PKCβ significantly enhanced melanoma sensitivity to erastin or RSL3 treatment. In contrast, overexpression of a dominant‐negative (DN) PKCβ mutant reduced tumor responsiveness to erastin or RSL3 (Figure 7B–G). These findings suggest that PKCβ expression levels may influence the efficacy of ferroptosis‐inducing therapies, highlighting PKCβ as a promising biomarker for identifying cancer patients who may benefit from ferroptosis‐targeted treatments.

### 2.8 Inhibition of Ca^2+^‐PKCβ Signal Protects Pancreas from Acute Pancreatitis by Suppressing Ferroptosis

**FIGURE 8:**
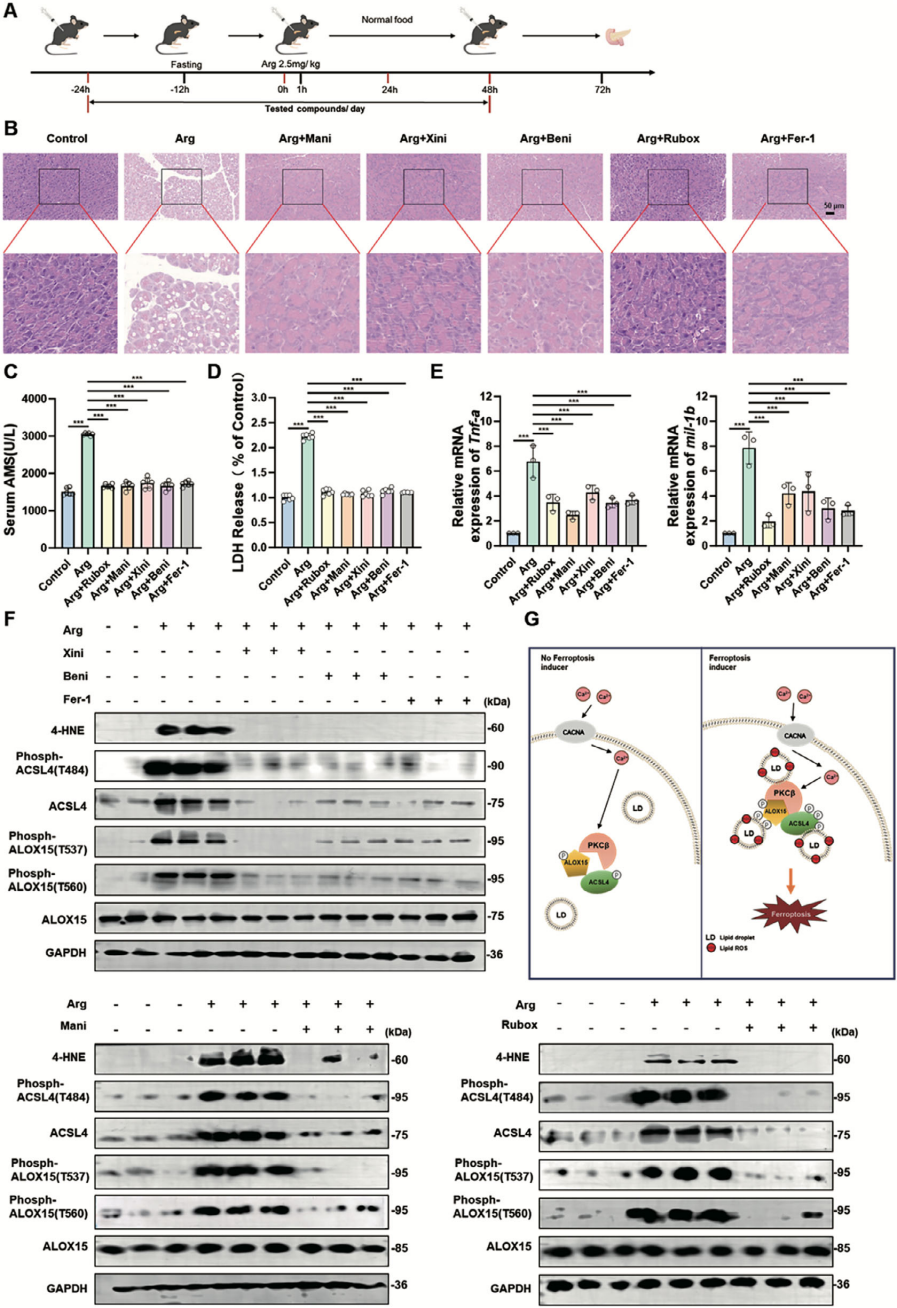
Inhibition of Ca^2+^‐PKCβ Signal Protects Pancreas from Acute Pancreatitis by Suppressing Ferroptosis. A) A schematic diagram shows the procedure of arginine‐induced acute pancreatitis experiment. B) Ferroptosis inhibitors treatment (L‐type calcium channel blockers, PKCβ inhibitor or ferrostatin‐1) protects from arginine induced acute pancreatitis analyzed by HE staining. C57 mice were treated as Figure 8a, then scarified and pancreatic tissue was fixed and stained by HE. Arg: (2.5 mg/kg) L‐type calcium channel blockers: Xini (xinidipine, 5 mg/kg), Beni (Benidipine, 5 mg/kg), Mani (manidipine, 5 mg/kg), PKCβ inhibitor: Rubox (ruboxistaurin, 1.5 mg/kg) or Fer‐1 (ferrostatin‐1, 10 mg/kg). C) Ferroptosis inhibitors protect from arginine induced acute pancreatitis shown by serum amylase (AMS). C57 mice were treated as Figure 8A,B, then scarified and serum amylase was analyzed by α‐amylase assay kit. D) Ferroptosis inhibitors protect from arginine induced acute pancreatitis shown by serum lactate dehydrogenase (LDH). C57 mice were treated as Figure 8A,B, then scarified and serum lactate dehydrogenase was analyzed by lactate dehydrogenase assay kit. E) Ferroptosis inhibitors protect from arginine induced acute pancreatitis shown by the expression of pro‐inflammatory cytokines (IL‐1β and TNF‐α) in pancreatic tissue. C57 mice were treated as Figure 8A,B, then scarified and mRNA was isolated from pancreatic tissue and analyzed by RT‐PCR. F) Ferroptosis inhibitors blocked arginine induced acute pancreatitis associated ferroptosis. Western blot images confirmed the expression of the indicated proteins. G) the working model showing the mechanism that calcium‐dependent phosphorylation and lipid droplets relocation of ACSL4 ‐PKCβ‐ ALOX15 complex dictate ferroptosis. Data are derived from three independent experiments, and each value represents the mean ± SD. ^*^ *p* < 0.05, ^**^ *p* < 0.01, ^***^ *p* < 0.001, *t* test. (*n* = 6).

Acute pancreatitis (AP) is a severe inflammatory condition characterized by extensive tissue damage and cellular death. Emerging evidence implicates ferroptosis in the pathogenesis of AP [39]. Given our previous finding that the Ca^2+^‐PKCβ signaling pathway positively regulates ferroptosis, we investigated whether inhibition of this pathway could mitigate pancreatic injury in a arginine‐induced AP model as illustrated in Figure 8A. Arginine administration induced severe AP, as evidenced by HE staining (Figure 8B), a marked increase in serum amylase (AMS; Figure 8C) and lactate dehydrogenase (LDH; Figure 8D) levels, alongside elevated expression of pro‐inflammatory cytokines (IL‐1β and TNF‐α) in pancreatic tissue (Figure 8E). Treatment with the ferroptosis inhibitor ferrostatin‐1 significantly attenuated these pathological changes, suggesting a protective role of ferroptosis inhibition in AP (Figure 8B–E).

To further explore the therapeutic potential of targeting Ca^2+^‐PKCβ signaling in AP, we evaluated the effects of L‐type calcium channel blockers (Benidipine, Xinidipine, and Manidipine) and the PKCβ inhibitor ruboxistaurin. Both L‐type calcium channel blockers and ruboxistaurin effectively alleviated arginine‐induced AP, as demonstrated by HE staining, reduced serum AMS and LDH levels and decreased expression of inflammatory cytokines (Figure 8B–E). Arginine‐induced AP was associated with elevated levels of 4‐hydroxynonenal (4‐HNE), a marker of lipid peroxidation and upregulated expression of ACSL4 (Figure 8F). More importantly, arginine treatment induced dramatical phosphorylation of ACSL4 (T484) and phosphorylation of ALOX15 (both T537 and T560) (Figure 8F). Co‐treatment with ferrostatin‐1, L‐type calcium channel blockers, or ruboxistaurin suppressed these changes (Figure 8F), further supporting the involvement of ferroptosis and Ca^2+^‐PKCβ mediated phosphorylation of ACSL4 and ALOX15 signal in AP pathogenesis. Our findings highlight the critical role of ferroptosis in AP and suggest that pharmacological inhibition of Ca^2+^‐PKCβ signaling, either through L‐type calcium channel blockers or PKCβ inhibitors, represents a promising therapeutic strategy for mitigating pancreatic injury in AP.

## 3 Discussion

Collectively, we identified several L‐type calcium channel blockers as novel inhibitors of ferroptosis. We further elucidated that calcium‐dependent activation of PKCβ drives ferroptosis by phosphorylating key enzymes, ACSL4 and ALOX15, at multiple sites. Upon ferroptosis induction, the PKCβ‐ACSL4‐ALOX15 complex translocate to lipid droplets, suggesting a crucial role of lipid droplets in ferroptosis. More importantly, inhibition of the Ca^2^^+^‐PKCβ signaling pathway protects the pancreas from acute pancreatitis by suppressing ferroptosis. These findings suggest that modulation of Ca^2^^+^‐PKCβ‐mediated ferroptosis holds significant promise for developing novel therapeutic strategies for these diseases.

Pedrera et al., recently reported that Ca^2+^ fluxes can activate the ESCRT‐III‐dependent membrane repair machinery to counterbalance the kinetics of ferroptosis, suggesting the anti‐ferroptosis function of calcium [30]. However, we here found that blockage of calcium uptake by L‐type calcium channel blockers suppress ferroptosis. Furthermore, depletion of cellular calcium by calcium chelators inhibits ferroptosis. These data indicate the pro‐ferroptosis function of calcium signaling.

Zhang et al., recently reported that PKCβII but not PKCβI promotes ferroptosis by phosphorylate Thr^328th^ of ACSL4 [36]. We here found that both PKCβI and PKCβII positively regulate ferroptosis, although PKCβII has much stronger effect in promoting ferroptosis compared to PKCβI. We also identified a novel PKCβ phosphorylation site, Thr^484th^ of ACSL4. Furthermore, we showed that ALOX15 is another substrate of PKCβ and PKCβ phosphorylates ALOX15 at the sites Ser^9th^, Thr^539th^, and Thr^560th^. We further developed specific antibodies against p‐ACSL4^T484^, p‐ALOX15^T537^, and p‐ALOX15^T537^, and we used these antibodies confirming that PKCβ mediated phosphorylation of ACSL4 and ALOX15 can only be detected in ferroptosis but no other kinds of regulated cell death, highlighting the specificity phosphorylation of ACSL4 and ALOX15 in ferroptosis. We also confirmed these phosphorylation antibodies can be used to detect ferroptosis in vivo. Lack of specific markers has delayed the progress of ferroptosis study. We believe these specific phosphorylation antibodies are important tools for the study of ferroptosis biology.

Furthermore, our study reveals the dynamic nature of the ACSL4‐PKCβ‐ALOX15 complex. Our data suggest that PKCβ acts as a central scaffold. Under basal conditions, it facilitates the interaction between ACSL4 and ALOX15, potentially keeping the system in a poised state. Upon ferroptosis induction and calcium‐dependent PKCβ activation, we observe a critical shift: the interaction between ALOX15 and PKCβ is enhanced, while the interaction between ALOX15 and ACSL4 is weakened. A model is proposed as following: Phosphorylation of ACSL4 (at T484) and ALOX15 (at S9, T537, T560) by activated PKCβ likely induces conformational changes. This phosphorylation disrupts the ACSL4‐ALOX15 interface, leading to the dissociation of the “closed” ternary complex. The result is the formation of active PKCβ‐ALOX15 and PKCβ‐ACSL4 subcomplexes on the lipid droplet (LD) membrane. This spatial reorganization is crucial because: It may release autoinhibitory constraints on ALOX15, enhancing its ability to peroxidize lipids. It positions the enzymes in proximity to their PUFA‐CoA and phospholipid substrates concentrated within or on the surface of LDs. This model explains why phosphorylation‐mimetic mutants (ACSL4‐T484E, ALOX15‐T537E, ALOX15‐T560E) are constitutively active and localize to LDs even in the absence of full ferroptosis induction.

Lipid droplets (LDs) are dynamic, ER‐derived organelles that serve as lipid storage compartments and play a context‐dependent role in regulating ferroptosis. In certain cell types, the sequestration of polyunsaturated fatty acids (PUFAs) within LDs—shielding them from incorporation into membrane phospholipids—reduces cellular susceptibility to ferroptosis [40, 41]. Conversely, inhibition of system Xc^–^ combined with dehydroascorbic acid treatment induces ferroptosis in a manner associated with LD oxidation [42]. We demonstrate that genetic or pharmacological inhibition of key LD regulators, including DGATs and FITM2, suppresses ferroptosis, whereas DGAT1 or FITM2 overexpression exacerbates it. Moreover, lipid peroxides induced by erastin or RSL3 colocalize with LDs. Notably, under ferroptotic conditions, the ACSL4‐PKCβ‐ALOX15 complex translocate to LDs. LDs may serve as a platform that concentrates the enzymes and substrates, thereby dramatically increasing the efficiency of lipid peroxidation. The assembly and activity of the complex are indeed regulated by LDs, as evidenced by the fact that disrupting LD biogenesis prevents the ferroptosis‐induced translocation and complex phosphorylation. Our data with the phosphomimetic mutants (ACSL4‐T484E, ALOX15‐T537E and ALOX15‐T560E) show an increase in the number of LD‐associated puncta but a decrease in their size. This phenotype is consistent with an increase in LD nucleation or a fragmentation of existing LDs. Crucially, these cells exhibit enhanced ferroptosis sensitivity. This suggests that the increased surface area provided by a larger number of smaller LDs may facilitate more efficient access for the ACSL4‐PKCβ‐ALOX15 complex to lipid substrates, thereby amplifying the peroxidation signal.

Ferroptosis has significant implications for various diseases and modulation of ferroptosis holds great potential to treat these diseases. Acute pancreatitis (AP) is an inflammatory condition of the pancreas that can cause local injury, systemic inflammatory response syndrome, and organ failure [43]. The mechanism of cell damage during acute pancreatitis has not been fully elucidated. In recent years, various regulatory cell deaths (RCDs), such as apoptosis, pyroptosis, autophagy, and necroptosis, have also been found to play important roles in the pathogenesis of AP [44]. The progression of AP is closely related to the regulatory transitions between different RCDs. Ferroptosis is an iron dependent RCD. Here, we further confirmed the crucial role of ferroptosis in arginine‐induced acute pancreatitis, as evidence by elevated 4‐HNE and MDA, two biomarkers of ferroptotic lipid peroxidation. More importantly, inhibition of the Ca^2^^+^‐PKCβ signaling pathway protects against acute pancreatitis by suppressing ferroptosis. The L‐type calcium channel blockers are used to treat conditions of the heart or blood vessels including high blood pressure, angina, arrhythmias, and circulatory disorders. Here we found that they can protect against arginine induced acute pancreatitis in the mouse model. Since there is still no specific or effective treatment for acute pancreatitis, clinical trials to evaluate the efficacy of L‐type calcium channel blockers in managing this condition should be conducted as soon as possible. In addition, we found that elevated PKCβ levels enhance the efficacy of ferroptosis‐inducing cancer therapies, highlighting PKCβ as a promising biomarker for identifying cancer patients who may benefit from ferroptosis‐inducing treatments. We also confirmed the inhibition effect of L‐type calcium channel blockers on ferroptosis using human cardiomyocyte AC16 cells, which are highly relevant for calcium biology and ferroptosis in ischemia‐reperfusion injury. These findings underscore the therapeutic potential of targeting Ca^2^^+^‐PKCβ‐mediated ferroptosis, offering new avenues for the treatment of acute pancreatitis and other ferroptosis related diseases.

## 4 Experimental Section

### 4.1 Cell Culture

The following cell lines were obtained from the American Type Culture Collection (ATCC): Human fibrosarcoma HT1080 (CCL‐121), human HEK293T (CRL‐3216), human glioblastoma U373 (HTB‐17), human breast adenocarcinoma MDA‐MB‐231 (HTB‐26), murine embryonic fibroblasts MEF (SCRC‐1008), human cardiomyocyte AC16 (CRL‐3568) and murine connective tissue L‐929 (CCL‐1). Cell lines were maintained in DMEM (Biological Industries) with high glucose, sodium pyruvate (1 mM), glutamine (4 mM), penicillin (100 U/mL), streptomycin (0.1 mg/mL) and 10% (v/v) FBS at 37°C and 5% CO_2_. All the cell lines were free of mycoplasma contamination.

### 4.2 Chemicals and Critical Commercial Assays

A list of all Chemicals and Critical commercial kits used in this study can be found as following. Discovery Probe‐FDA‐approved‐Drug‐Library (Cat: L1021, APExBIO), PKC412 (Midostaurin; Cat: HY‐10230, MCE), Ruboxistaurin (LY333531; Cat: HY‐10195, MCE), EDTA‐AM (Cat: HY‐D1746, MCE), EGTA‐AM (Cat: HY‐D0973, MCE), BHQ (Cat: S3628, Selleckchem), Ferrostatin‐1(Fer‐1; Cat: HY‐100579, MCE), Deferoxamine (Cat: HY‐B1625 MCE), Erastin (Cat: HY‐15763, MCE), RSL3 (Cat: HY‐100218A, MCE), Xinidipine (Cat: 132203‐70‐4, MACKLIN), Benidipine (Cat: 105979‐17‐7, MACKLIN), Manidipine (Cat: 89226‐50‐6, MACKLIN), Lacidipine (Cat: 103890‐78‐4, MACKLIN), Nisoldipine (Cat: N858016, MACKLIN), Z‐VAD‐FMK (Cat: 161401‐82‐7, TargetMol), Lipi‐Blue (Cat: L247‐LD01, Dojindo), Liperfluo (Cat: NG661, Dojindo), BODIPYTM 493/503 (Cat: D3922, Invitrogen), Nile Red (Cat: 7385‐67‐3, MACKLIN), Lipofectamine 2000 (Cat: 11668‐019, Thermo Fisher), Anti‐fluorescence fade sealing reagent (Cat: S2100, Solarbio), PF‐04620110 (Cat: T6937, TargetMol), PF‐06424439 (Cat: T12425, TargetMol), Ingenol‐5,20‐acetonide‐3‐O‐angelate (Cat: TQ0316, TargetMol), Protease Inhibitor Cocktail (Cat: K1007, APExBIO), Taq PCR Master Mix (Cat: K1034, APExBIO), RIPA lysate (Cat: PR20035, Proteintech), Mycoplasma Removal Agent Plus (Cat: C0290S, Beyotime), Penicillin‐Streptomycin (Cat: P1400, Solarbio), Trypsin‐EDTA (Cat: 25200072, Gibco), BayK8644 (Cat: 98625‐26‐4, MACKLIN), Raptinal (Cat: 1176‐09‐6, TargetMol), BODIPYTM 581/591 (Cat: 217075‐36‐0, TargetMol), TNF‐α (Cat: TMPY‐00936, TargetMol), propidium iodide (Cat: C0080, Solarbio), Smac mimetics (Cat: T2080, TargetMol), ML218 (Cat: T12076L, TargetMol),

#### 4.2.1 Critical Commercial Assays

Pierce ECL Plus Western Blotting Substrate (Cat: 32209, Thermo Fisher Scientific), SYBR Green PCR Master Mix (Cat: A46109, Thermo Fisher), Prime Script RT reagent Kit with gDNA Eraser (Perfect Real Time; Cat: RR047A, Takara Bio), TaqMan Universal PCR Master Mix (Cat: 4304437, Thermo Fisher), Gene JET Gel Extraction Kit (Cat: K0691, Thermo Fisher), Axygen Prep DNA Gel Extraction Kit (Cat: AP‐MX‐P‐25, Axygen), TaqMan Reverse Transcription (Cat: N8080234, Thermo Fisher Scientific), Mycoplasma Removal Agent (Cat: C0288M, Beyotime), TIAN prep Mini Plasmid Kit (Cat: DP103‐03, TIANGEN), Enhanced BCA Protein Assay Kit (Cat: P0010, Beyotime), Lightening cloning Kit (Cat: BDIT0014, Biodragon), Protein A/G Magnetic Beads (Cat: HY‐K0202, MCE).

### 4.3 Antibodies

A list of primary antibodies and secondary antibody used in this study can be found as following. Beta Actin Recombinant antibody (Cat: 81115‐1‐RR, Proteintech), anti‐human α‐Tubulin Antibody (Cat: T6074, Sigma‐Aldrich), HA Tag Recombinant Antibody (Cat: 51064‐2‐ap, Proteintech), Mouse monoclonal GAPDH Antibody (Cat: 60004‐1‐Ig, Proteintech), Mouse monoclonal GPX4 Antibody (Cat: 67763‐1‐Ig, Proteintech), PRKCB Monoclonal Antibody (Cat: 68620‐1‐Ig, Proteintech), DYKDDDDK tag Recombinant Antibody (Cat: 80010‐1‐RR, Proteintech), Phospho‐(Ser) PKC Substrate Antibody (Cat: 2261S, Cell Signaling Technology), Rabbit monoclonal anti‐15 Lipoxygenase 1 Antibody (Cat: ab244205, Abcam), Phospho‐PKC (pan) (βIISer660) Antibody Cell Signaling Technology (Cat: 9371, Cell Signaling Technology), Rabbit monoclonal Glutathione Peroxidase 4 Antibody (Cat: ab125066, Abcam), Anti‐Glutathione Peroxidase 4 Antibody (ab40993, Abcam), Mouse monoclonal ACSL4 Antibody (Cat: sc‐271800, Santa Cruz Biotechnology), 4‐HNE Antibody (Cat: MA5‐27570, Invitrogen), GSDME Antibody (Cat: HA723251, HUABIO), SLC7A11/xCT Polyclonal antibody (Cat: 26864‐1‐ap, Proteintech), DHODH Polyclonal antibody (Cat: 14877‐1‐AP, Proteintech), FSP1/AIM2 Antibody (Cat: 68049‐1‐Ig, Proteintech), Alox5 Antibody (Cat: GB111330, Servicebio), ALOX12 Antibody (Cat: GB114423, Servicebio), LPCAT3 Antibody (Cat: HA723171, HUABIO), RIP3 Rabbit Polyclonal Antibody (Cat: ER1901‐27, HUABIO), Phospho‐RIP3 (S232) Recombinant Rabbit Monoclonal Antibody (HA721428, HUABIO), MBAOT2 Antibody (Cat: AP17786C, abcepta),Anti‐HA tag Antibody (HA721750, HUABIO), Caspase3 Antibody (Cat: 19677‐1‐AP, Proteintech), Anti‐PKCγ (WLO2938, Wanlei), Anti‐PKCα (WLO2234, Wanlei), Phospho‐ACSL4 (T484) Recombinant Rabbit polyclonal Antibody (This study, HA500600, HUABIO), Phospho‐ALOX15 (T537) Recombinant Rabbit polyclonal Antibody (This study, HA500601, HUABIO), Phospho‐ALOX15 (T537) Recombinant Rabbit polyclonal Antibody (This study, HA500599, HUABIO).

### 4.4 Generation of Stable Cells

Plasmids to generate overexpression cell lines are listed as following: Dominant‐negative (DN) PKC isoforms: PKCβII‐DN (# 16385, Addgene), PKCβI‐DN (# 16381, Addgene), PKCα‐DN (# 21235, Addgene), PKCγ‐DN (# 21239, Addgene). Calcium channel overexpression constructs in pLV3‐CMV: Human FITM2 (# P48832, MiaoLing Bio), Rat Cacna1s (# P71183; MiaoLing Bio), Rat Cacna1c (# P49161, MiaoLing Bio), Rat Cacna1d (provided by MiaoLing Bio), Rat Cacna1f (# P70996, MiaoLing Bio). Other plasmids were generated in this study.

Specific small‐hairpin RNA (shRNA) sequences targeting human or mouse genes were got from Sigma‐Aldrich Advanced Genomics RNAi Libraries. The TRC numbers of shRNAs are listed as following: Non‐targeting control: pLKO.1‐NT (8453, Addgene), PKCβ: shPKCβ#1 (TRCN0000003117), shPKCβ#2 (TRCN0000196841), ALOX15: shALOX15#1 (TRCN0000420755), shALOX15#2 (TRCN0000056579), ACSL4: shACSL4#1 (TRCN0000045540), shACSL4#2 (TRCN0000045541), shDGAT1 (TRCN0000236203), FITM2: shFITM2#1 (TRCN0000125380), shFITM2#2 (TRCN0000125381).

Retrovirus or lentivirus were packaged in 293T cells and used to infect target cells, which were then selected with puromycin for at least 3 days prior to use in experiments.

### 4.5 Immunoprecipitation and Immunoblotting

Cells were lysed in IP buffer (20 mM Tris‐HCl pH 8.0, 137 mM NaCl, 1% NP‐40, 2 mM EDTA, 1% Proteases Inhibitor Cocktail). The 4 × 10^3 ^µg cell lysates were incubated overnight with 3 µg specific primary antibody against indicated protein or IgG antibody (Biodragon, Cat#BF02008) at 4°C. The mix of cell lysates and antibody then were incubated overnight with 25 µL protein A/G magnetic beads (MCE, Cat#HY‐K0202) at 4°C. Beads were washed with IP buffer 3 times, collected with IP buffer, then analyzed by WB assay.

### 4.6 Measurement of Cell Death

Cells treated as indicated were incubated with 1 µL of 10 mg/mL propidium iodide (C0080, Solarbio) at 37°C for 30 min. Collect both the culture medium and the digested cells into a 1.5 mL tube, and then perform centrifugation (2 000–3 000 g, 5 min) at 4°C. The supernatant was discarded, and the pellet was gently washed with 1 mL of PBS. Cells in a 12‐well plate were trypsinized using serum‐free medium, incubated at 37°C, and digestion was terminated by adding an equal volume of serum‐containing medium. The cell suspension was transferred to a 1.5 mL EP tube, centrifuged (1,000 g, 5 min) at 4°C, resuspended in 1 mL PBS, and recentrifuged under the same conditions. The final pellet was resuspended in 200–300 µL of PBS containing 5% FBS, combined with the corresponding pellet from the same well, and filtered into flow cytometry tubes for analysis.

### 4.7 Measurement of Lipid ROS

Cells were seeded at a density of 3.5 × 10^5^ per well in a 6‐well dish and grow overnight in DMEM. 5 µM BODIPY C11 was added into cell culture medium and incubated for 30 min after indicated treatment. Excess fluorescent probe was then removed by washing the cells with PBS twice. Labeled cells were trypsinized and resuspended in PBS plus 2% FBS. Oxidation of BODIPY C11 resulted in a shift of the fluorescence emission peak from 590 to 510 nm were measured by flow cytometry.

### 4.8 Confocal Microscopy and Immunofluorescence

Wild‐type HT1080 cells were seeded at 5 × 10^4^ cells/well on glass coverslips in 12‐well plates for 24 h. After indicated treatment, cells were stained with specific dyes as indicated (1 µM Liperfluo or 5 µM of BODIPYTM 581/591 for lipid ROS, and 2 µM BODIPY 493/503, 1 µM Lipi‐Blue or 200 µM Nile Red for lipid droplets) in serum‐free medium at 37°C for 30 min protected from light. Cells were then washed 5 times with PBS for 5 min and 3 times with HBSS for 3 min. Coverslips were mounted in 15 µL HBSS on microscope slides and immediately imaged using a Zeiss LSM 800 confocal system.

HT1080 cells with indicated tags were seeded at 5 × 10^4^ cells/well on glass coverslips in 12‐well plates for 24 h. After indicated treatment, cells were incubated with lipid droplets dye (2 µM BODIPY 493/503, 1 µM Lipi‐Blue or 200 µM Nile Red for lipid droplets) for 30 min at 37°C in the dark, then fixed with 4% paraformaldehyde for 15 min, and permeabilized with 0.1% Triton X‐100 for 10 min at 22°C. Cells on coverslips were then washed 5 times with PBS for 5 min, and incubated with anti‐ACSL4 mouse monoclonal antibody (sc‐271800, Santa Cruz Biotechnology; 1:200 in 5% BSA/PBS) overnight at 4°C, after washed 6 times with PBS, cells on coverslips were incubated with Alexa Fluor 647‐conjugated goat anti‐mouse IgG for 1 h at 22°C in the dark. After three additional 5‐min PBS washing, coverslips were mounted with Pro Long Diamond Antifade Mountant and imaged on confocal microscope using a 63×/1.40 Oil objective with the following settings. eGFP:488 nm laser, Lipi‐Blue:405 nm laser; Cherry:594 nm laser; Alexa Fluor 647:633 nm laser.

### 4.9 Real‐Time Quantitative PCR

Total RNA was extracted using TRIzol reagent (Cat: 15596026, Thermo Fisher) according to the manufacturer's instructions. cDNA was synthesized using the cDNA Synthesis Kit (Cat: RR047A, Takara) according to the manufacturer's instructions. RT–qPCR was performed with universal SYBR qPCR Master Mix Kit (Cat: Q511‐03, Vazyme) in a Real‐Time PCR system (Applied Biosystems). Primer sequences are listed in supplementary material.

### 4.10 Measurement of Intracellular Calcium Concentration by Flow Cytometry

Intracellular calcium levels were assessed using the fluorescent dye Fluo‐4 AM (Yeasen Biotechnology, Cat# 10184ES) followed by flow cytometric analysis. Briefly, cells were washed twice with PBS buffer. HT1080 cells were stained by adding the Fluo‐4 AM stock solution (1 mM) to the loading buffer (Hanks' Balanced Salt Solution with 20 mM HEPES, pH 7.4) at a final concentration of 2 µM. The cells were incubated in the dark at 37°C for 45 min, with gentle shaking of the culture plate every 15 min. Subsequently, cells were washed twice with pre‐warmed, dye‐free HBSS‐HEPES buffer to remove extracellular dye, trypsinized, and centrifuged at 800 × g for 5 min. The cell pellet was then resuspended in fresh buffer and incubated for an additional 20 min at 37°C in the dark to ensure complete de‐esterification of the intracellular AM esters before subjected to flow cytometric analysis.

### 4.11 In Vitro Kinase Assay

The Flag‐ACSL4 or Flag‐ALOX15 (wildtype or mutants) and/or HA‐PKCβ‐WT plasmids were co‐transfected into 293T cells as indicated. Cells were incubated for 48 h, then proteins were purified by using anti‐FLAG beads or anti‐HA beads. Purified Flag‐ACSL4 or Flag‐ALOX15 (wildtype or mutants) was incubated with or without purified HA‐PKCβ‐WT in kinase buffer (15 mM Tris‐HCL, 0.2 mM MgCl2, 0.2 mM ATP pH 7.5) at 30°C for 30 min. The reactions were stopped by adding SDS‐PAGE loading buffer and boiling for 5 min. Proteins were then immunoblotted with the indicated antibodies.

### 4.12 ALOX15 Activity Assay

Cellular ALOX15 enzymatic activity was assessed using a commercial Lipoxygenase (LOX) Activity Assay Kit (Suzhou Grace Biotechnology Cat# G0906W) according to the manufacturer's instruction.

### 4.13 ACSL4 Activity Assay

Cellular ACSL4 enzymatic activity was accessed using a commercial Acetyl‐CoA Synthetase Activity Assay Kit (Beyotime, Cat#S0391S) according to the manufacturer's instruction.

### 4.14 2,2‐Diphenyl‐1‐Picrylhydrazyl Assay for Antioxidant Activity

The experiment was performed as described previously [45]. 2,2‐Diphenyl‐1‐picrylhydrazyl (DPPH) (Sigma Cat#D9132) was dissolved in methanol to a final concentration of 50 µM. The tested compounds were added to 1 mL of 50 µM DPPH solution. Samples were mixed well and incubated at room temperature for 1 h. The absorbance at 517 nm (indicating the concentration of non‐reduced DPPH) was measured using methanol as a negative control.

### 4.15 Xenograft Mouse Model

All animal studies were approved by the Animal Experimental Ethics Committee of Harbin Institute of Technology (licence no.: IACUC‐2023052 and IACUC‐2024047). 6–8 weeks old Male C57/B6 mice were purchased from Liaoning Changsheng biotechnology Co., Ltd. 2 × 10^6^ B16‐F10 stable cells as indicated were injected subcutaneously into one flank of each mouse. Tumor volumes were measured daily until the tumor sizes reached about 65 mm^3^. Then, 1 mg/kg body weight (BW) of RSL3 (MCE, HY‐100218A) was administered via intraperitoneal injection daily for 7 days. Tumor size was measured daily. After 7 days, tumors were collected, photographed, and weighted. A minimum of six mice were used for each group.

### 4.16 Mice Model of Acute Pancreatitis

All animal studies were approved by the Animal Experimental Ethics Committee of Harbin Institute of Technology (licence no.: IACUC‐2023052 and IACUC‐2024047). C57BL/6 mice were purchased from Liaoning Changsheng biotechnology Co., Ltd. Inducing L‐Arginine acute pancreatitis was performed as previous study with minor modifications [46, 47]. Briefly, 8‐week‐old C57BL/6 mice were pretreated with individual tested compound (xinidipine, 5 mg/kg, Benidipine, 5 mg/kg, manidipine, 5 mg/kg, ruboxistaurin, 1.5 mg/kg or ferrostatin‐1, 10 mg/kg in vehicle (5% DMSO + 30% PEG300/PEG400 + 5% Tween‐80 + 60% Saline)) via intraperitoneal (i.p.) injection 24 h before L‐arginine administration. After a 12 h fast, mice received an i.p. injection of L‐arginine (2.5 g/kg, pH 7.2) along with the respective test compound, followed by a second L‐arginine injection (without compound) 1 h later. Tested compound was i.p. injected twice at 24 and 48 h post‐arginine‐induction. Mice were monitored for 72 h with free access to food and water before sacrifice. Blood was collected via cardiac puncture using heparinized syringes, centrifuged (4°C), and plasma was stored at ‐80°C for subsequent analysis of amylase, LDH, ALT, and MDA levels. Pancreatic tissue was harvested for histological (H&E staining), mRNA, and protein expression analysis.

### 4.17 HE Staining

The paraffin sections were immersed in sequence in Environmental Friendly Dewaxing Transparent Liquid I and II for dewaxing and hydration. The frozen sections were removed from the −20°C refrigerator and restored to room temperature, fixed with tissue fixating solution for 15 min, and then rinsed with running water. The sections were then treated with HD constant staining pretreatment solution for 1 min. Put sections into Hematoxylin solution for 3–5 min. Then treat the section with Hematoxylin Differentiation solution, Hematoxylin Bluing solution. Place the sections in 95% ethanol for 1 min, Eosin dye for 15 s. Finally, dehydration and sealing.

### 4.18 Statistical Analysis

All statistical analyses were performed using Prism 8.0 GraphPad Software. *P* values were calculated with unpaired Student's *t*‐test. All data are presented as mean ± SD from 3 independent experiments. *P* <0.05 was set as the threshold for significance (^*^ *P* < 0.05, ^**^ *P* < 0.01, ^***^ *P *< 0.001).

## Author Contributions

M Gao and W Zhang conceived and supervised the study. G Hou performed most experiments in collaboration with J Luan, J Qin, S Ma, J He, and N Sun. X Xu developed the antibodies against p‐ACSL4^T484^, pALOX15^T537^, or pALOX15^T560^. G Hou and M Gao analyzed the data and wrote the paper with suggestions from other authors. All authors reviewed the paper.

## Conflicts of Interest

X Xu is an employee of Hangzhou HuaAn Biotechnology. G Hou, J Qin, and M Gao have a patent application related to this study, and other authors declare no competing financial interests.

## Supporting information




**Supporting File 1**: advs74009‐sup‐0001‐SuppMat.docx.

**Supporting File 2**: advs74009‐sup‐0002‐Data.pdf.

## Data Availability

The data that support the findings of this study are available from the corresponding author (Minghui Gao) upon reasonable request.
