# Supplementary material for: Inhibition of Calcium‐Dependent Lipid Droplets Relocation of ACSL4‐PKCβ‐ALOX15 Complex Alleviates Ferroptosis and Acute Pancreatitis
Source: Adv Sci (Weinh). 2026 Jan 27;13(19):e15768. doi: 10.1002/advs.202515768 (PMC13045411; doi:10.1002/advs.202515768)
Supplement: Supplementary file 1 — Supporting File 1: advs74009‐sup‐0001‐SuppMat.docx. [file ADVS-13-e15768-s002.docx]

**
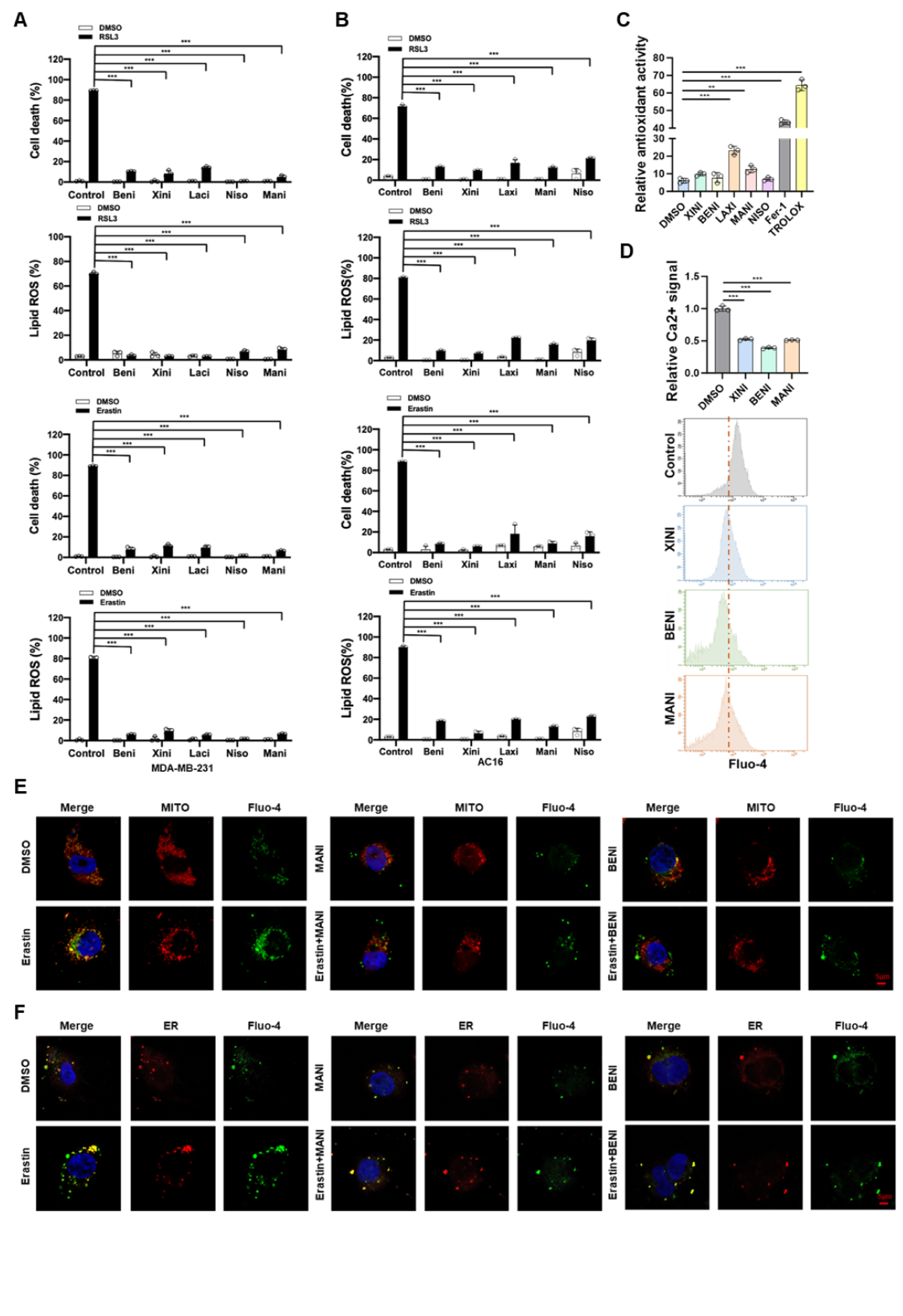

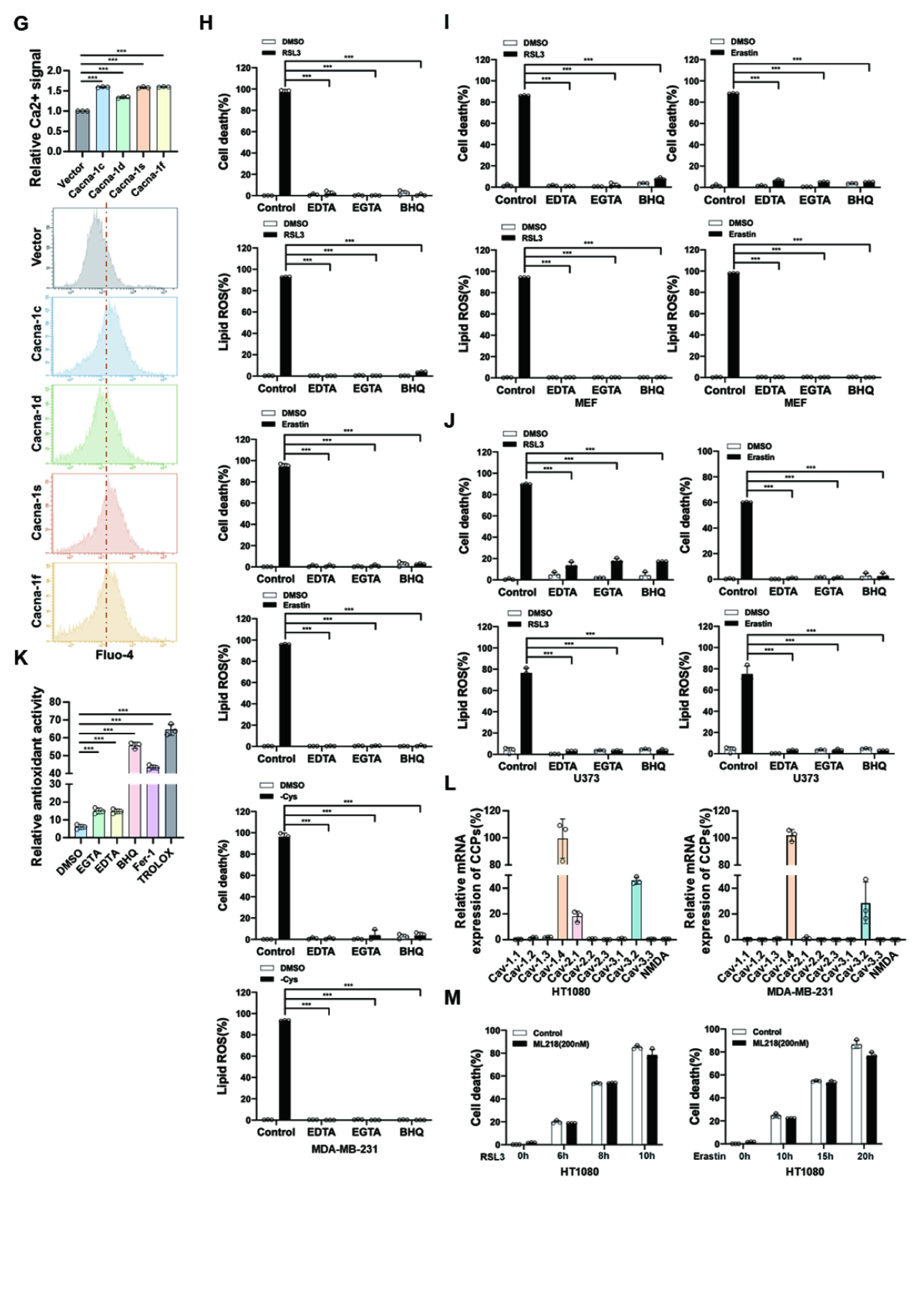
Figure S1 Multiple L-type calcium channel blockers suppress ferroptosis**

A) L-type calcium channel blockers treatment suppress erastin induced ferroptosis and lipid peroxidation. MDA-MB-231 cells were treated with RSL3 (0.5 μΜ, for 10 h) or erastin (20 μΜ, for 20 h) with or without l-type calcium channel blockers, then cell death and lipid ROS were measured as described in Figure 1b. Beni: Benidipine (50 µM), Xini: Xinidipine (20 µM), Laci: Lacidipine (40 µM), Niso: Nisoldipine (50 µM) and Mani: Manidipine (50 µM). B) L-type calcium channel blockers treatment suppress erastin induced ferroptosis and lipid peroxidation. AC16 cells were treated with RSL3 (0.5 μΜ, for 10 h) or erastin (20 μΜ, for 20 h) with or without l-type calcium channel blockers, then cell death and lipid ROS were measured as described in Figure 1b. Beni: Benidipine (50 µM), Xini: Xinidipine (20 µM), Laci: Lacidipine (40 µM), Niso: Nisoldipine (50 µM) and Mani: Manidipine (50 µM). C) The L-type calcium channel blockers have very little antioxidant activity measured by the DPPH assay. D) Treatment with the L-type calcium channel blockers individually significantly block calcium influx as shown by Fluo-4 AM staining coupled with flow cytometry analysis**.** E-F) L-type calcium channel blockers treatment decrease mitochondrial calcium (E) and endoplasmic reticulum calcium (F). G) Overexpression of L-type calcium channels promote calcium influx as shown by Fluo-4 AM staining coupled with flow cytometry analysis. H) Calcium chelators treatment suppress RSL3 or erastin induced ferroptosis and lipid peroxidation. MEFs were treated with RSL3 (2 μΜ, for 10 h) or erastin (10 μΜ, for 20 h) with or without calcium chelators, then cell death and lipid ROS were measured as described in Figure 1b. EDTA (40 μΜ), EGTA (50 μΜ), BHQ (50 μΜ). I) Calcium chelators treatment suppress RSL3 or erastin induced ferroptosis and lipid peroxidation. U373 cells were treated with RSL3 (1 μΜ, for 10 h) or erastin (10 μΜ, for 20 h) with or without calcium chelators, then cell death and lipid ROS were measured as described in Figure 1B. EDTA (40 μΜ), EGTA (50 μΜ), BHQ (50 μΜ). J) Calcium chelators treatment suppress RSL3, erastin or cystine starvation induced ferroptosis and lipid peroxidation. MDA-MB-231 cells were treated with RSL3 (0.5 μΜ, for 10 h), erastin (20 μΜ, for 20 h) or cystine starvation for 30 h with or without calcium chelators, then cell death and lipid ROS were measured as described in Figure 1B. EDTA (40 μΜ), EGTA (50 μΜ), BHQ (50 μΜ). K) EDTA-AM and EGTA-AM have very little antioxidant activity, but BHQ has similar antioxidant activity as ferrostatin -1 and Trolox. L) The expression of different calcium channels in HT1080 cells and MDA-MB-231 cells. M) T-type calcium channels inhibitor ML218 cannot block RSL3 or erastin induced lipid peroxidation and ferroptosis. HT1080 cells were treated with RSL3 (0.5 μΜ, for 10 h), erastin (20 μΜ, for 20 h) or cystine starvation for 30 h with or without calcium chelators, then cell death and lipid ROS were measured as described in Figure 1B. ML218 (200 nM).

Data are derived from three independent experiments, and each value represents the mean ± SD. **P < 0.05, **P < 0.01, ***P < 0.001*, *t* test.

**
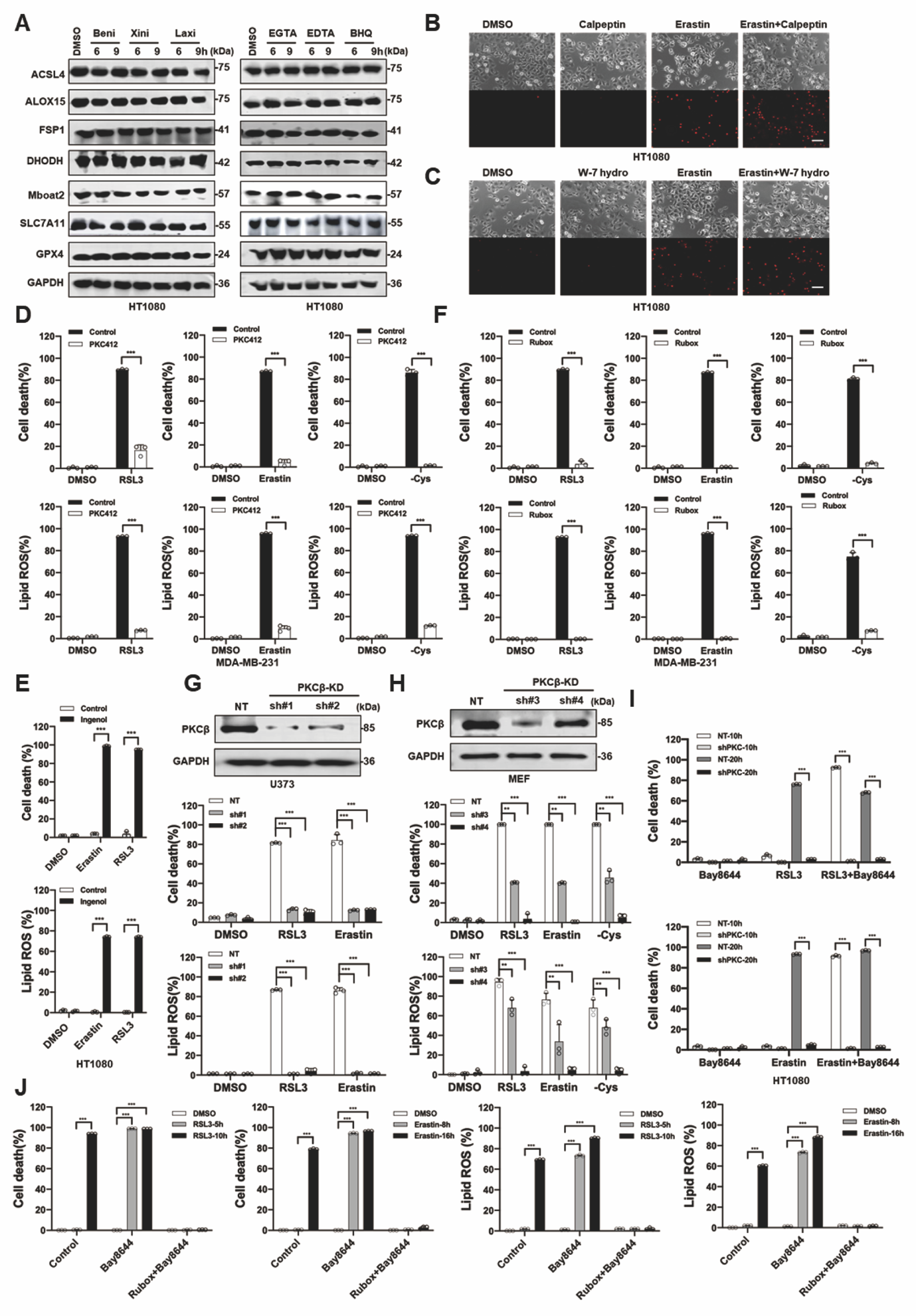
 Figure S2 Calcium dependent PKCβ activation is required for ferroptosis**

A) L-type calcium channel blockers or calcium chelators treatment do not regulate the expression of ferroptosis regulators in HT1080 cells. Western images confirmed the expression of indicated proteins. Beni (Benidipine, 50 μΜ), Xini (Xinidipine, 20 μΜ), Laci (Lacidipine, 40 μΜ), EDTA (40 μΜ), EGTA-AM (50 μΜ), BHQ (50 μΜ). B) Calpeptin (50 μΜ) treatment does not regulates erastin (10 μΜ) induced ferroptosis in HT1080 cells. C) W7 (50 μΜ) treatment does not regulates erastin (10 μΜ) induced ferroptosis in HT1080 cells. D) PKC412 (5 μΜ) treatment blocks RSL3 (0.5 μΜ, for 10 h), erastin (20 μΜ, for 20 h) or cystine starvation (for 30 h) induced ferroptosis and lipid peroxidation. MDA-MB-231 cells were treated as indicated, then cell death and lipid ROS were measured as described in Figure 1B. E) Ingenol (100 μΜ) treatment promotes erastin (10 μΜ, for 20 h) or RSL3 (2 μΜ, for 10 h) induced ferroptosis and lipid peroxidation. HT1080 cells were treated as indicated, then cell death and lipid ROS were measured as described in Figure 1B. F) Rubox treatment blocks RSL3 (0.5 μΜ), erastin (20 μΜ) or cystine starvation induced ferroptosis and lipid peroxidation. MDA-MB-231 cells were treated as indicated, then cell death and lipid ROS were measured as described in Figure1B. G) Knockdown of PKCβ suppress RSL3 (1 μΜ, for 10 h) or earstin (10 μΜ, for 20 h) induced ferroptosis and lipid peroxidation in U373 cells. Cells as indicated were treated as indicated, then cell death and lipid ROS were measured as described in Figure 1B. H) Knockdown of PKCβ suppress RSL3 (2 μΜ, for 10h) or erastin (10 μΜ, for 20 h) induced ferroptosis and lipid peroxidation in MEFs. Cells as indicated were treated as indicated, then cell death and lipid ROS were measured as described in Figure 1B. I) L-type calcium channel activator Bay K8644 (50 μΜ) promotes RSL3 (0.5 μΜ, for 10 h) or earstin (20 μΜ, for 20 h) induced ferroptosis, which can be blocked by knocking down of PKCβ in MDA-MB-231 cells. J) L-type calcium channel activator Bay K8644 (50 μΜ) promotes RSL3 (0.5 μΜ, for 10 h) or erastin (20 μΜ, for 20 h) induced ferroptosis, which can be blocked by PKCβ inhibitor rubox (5 μΜ) in MDA-MB-231 cells.

Data are derived from three independent experiments, and each value represents the mean ± SD. **P < 0.05, **P < 0.01, ***P < 0.001*, *t* test.

**
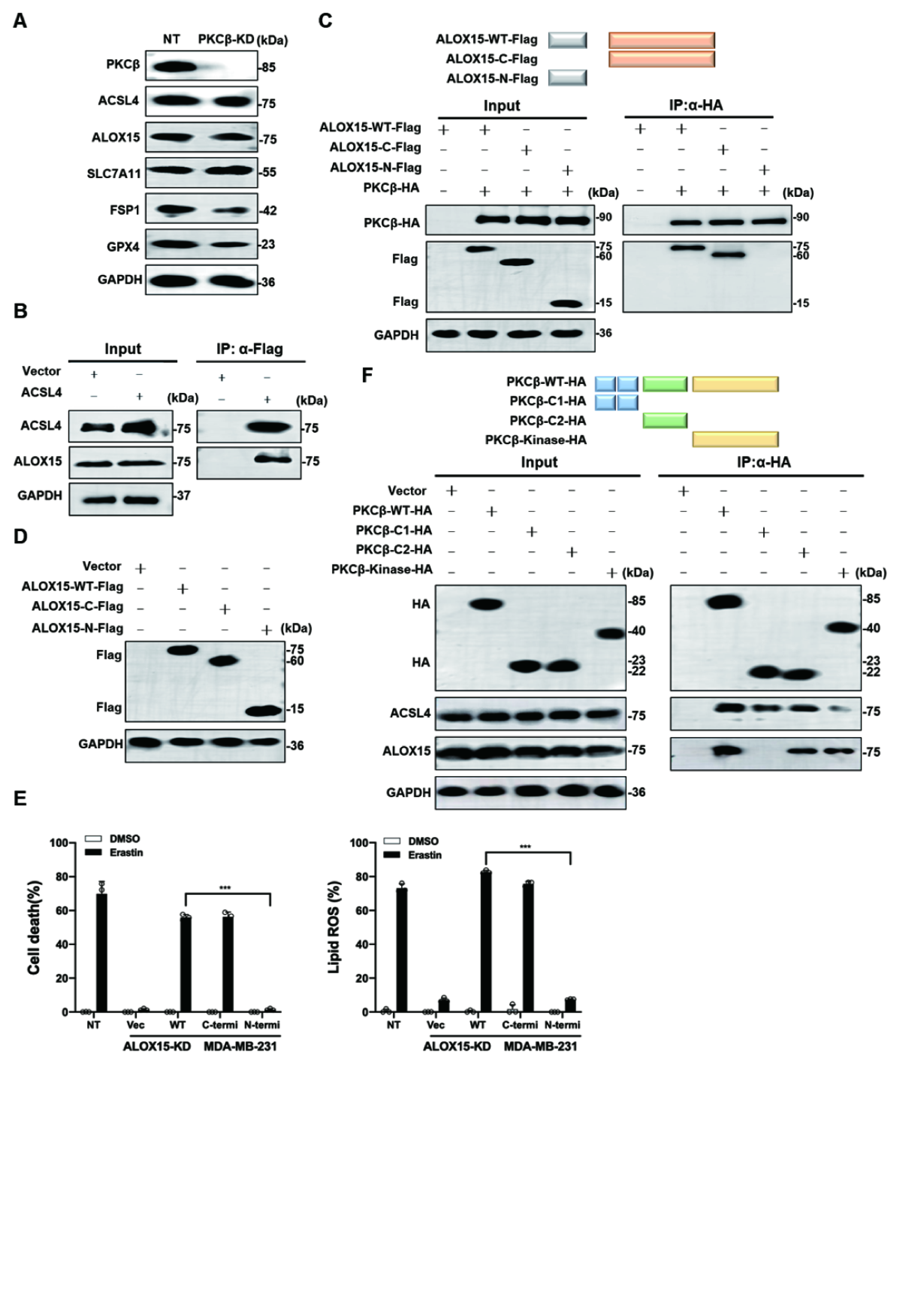
 Figure S3 PKC**β **forms a complex with ACSL4 and ALOX15**

A) The expression of ferroptosis regulators in PKCβ knockdown cells. B) ACSL4 interacts with ALOX15. The IP assay was performed using an antibody against FALG tag using indicated cell lysate. Western blot images confirm the expression of the indicated proteins. C) ALOX15 interacts with PKCβ through the C terminal of ALOX15. HA tagged ALOX15 or its domain as indicated were transient expressed in 293T cells. The IP assay was performed using an antibody against HA tag using indicated cell lysate. Western blot images confirm the expression of the indicated proteins. D-E) The C terminal of ALOX15 is required and sufficient for its function in ferroptosis. Western blot images confirm the expression of the indicated proteins. Indicated cells were treated with RSL3 (0.5 μΜ, for 10 h) or erastin (20 μΜ, for 20 h) for 20 h, then cell death and lipid ROS were measured as described in Figure 1B. F) The domain(s) of PKCβ mediates its interaction with ACSL4 or ALOX15. HA tagged PKCβ or its domain as indicated were transient expressed in 293T cells. The IP assay was performed using an antibody against HA tag using indicated cell lysate. Western blot images confirm the expression of the indicated proteins.

**
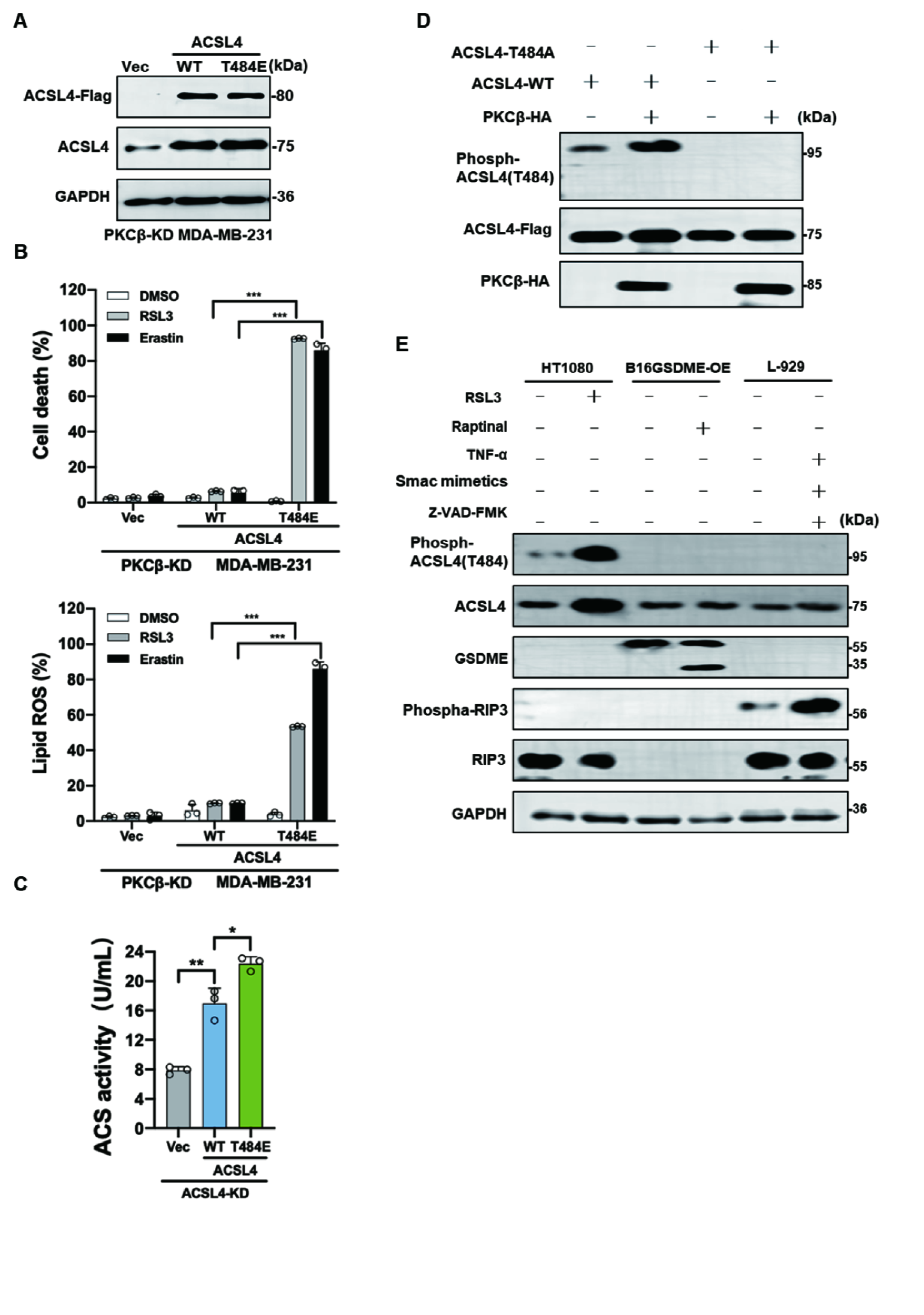
 Figure S4 The Thr^484th^ of ACSL4, a novel phosphorylated site by PKCβ, is required for ferroptosis**

A-B) Overexpression of ACSL4 with T484E phosphorylation mimic mutant promotes ferroptosis and lipid peroxidation in PKCβ KD MDA-MB-231 cells. PKCβ KD MDA-MB-231 cells overexpressed with ACSL4 T484E phosphorylation mimic mutant were treated with RSL3 (1 μΜ, 10h) or erastin (20 μΜ, 20 h), then then cell death and lipid ROS were measured as described in Figure 1B. C) The phosphomimetic ACSL4^T484E^ mutant has higher activity compared to wildtype ACSL4. The activity of ACSL4 in indicated stable cells was measured using the Acetyl-CoA Synthetase Activity Assay Kit. D) *In vitro* kinase assay confirmed that PKCβ directly phosphorylates wildtype ACSL4 but not ACSL4^T484A^ mutant. E) The phosphorylated ACSL4^T484^ is only detected in ferroptosis but not in pyroptosis or necroptosis. HT1080 cells were treated with RSL3 (1 μΜ, for 8h) to induce ferroptosis. GSDME overexpressed B16-F10 cells were treated with raptinal (30 μΜ, for 10h) to induce pyroptosis. L-929 cells were treated with T/S/Z (TNF-α, Smac mimetics and Z-VAD-FMK, for 4h) to induce necroptosis. Western images confirmed the expression of indicated markers. TNF-α: 20 ng/mL, Smac Mimetic: 10 µM, Z-VAD-FMK: 20 µM.

Data are derived from three independent experiments, and each value represents the mean ± SD. **P < 0.05, **P < 0.01, ***P < 0.001*, *t* test.

**
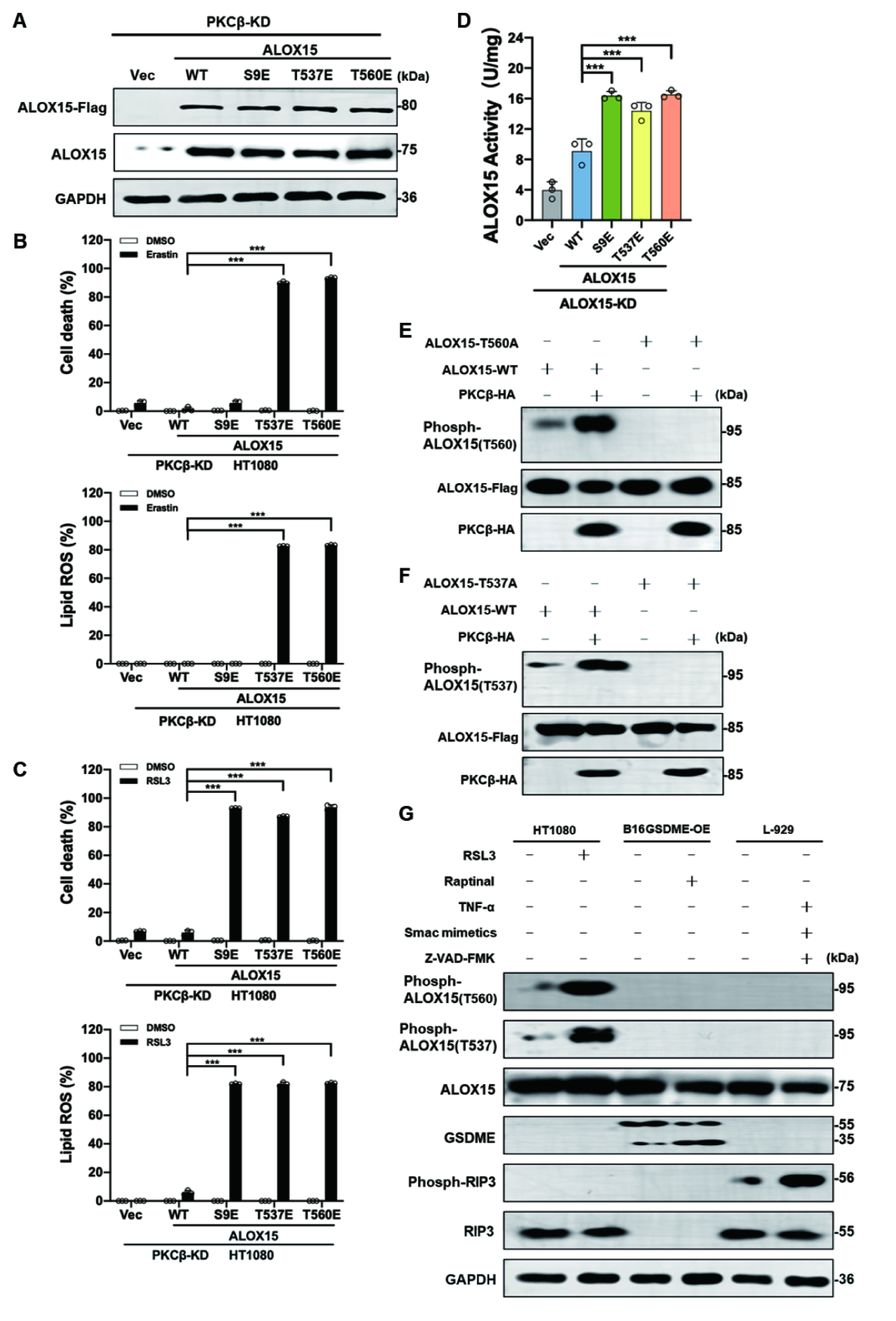
 Figure S5 Phosphorylation of multiple Ser/Thr sites of ALOX15 by PKCβ are required for ferroptosis**

A-C) Overexpression of ALOX15 with S9E, T537E or T560E phosphorylation mimic mutants promotes ferroptosis and lipid peroxidation in PKCβ KD HT1080 cells. Western blot images confirmed the expression of the indicated proteins (A). B-C) PKCβ KD HT1080 cells as indicated were treated with RSL3 (1 μΜ, for 10 h) or erastin (10 μΜ, for 20 h), then cell death and lipid ROS were measured as described in Figure 1b. D) the phosphomimetic mutants (ALOX15^T537E^, and ALOX15^T560E^) have higher activity compared to wildtype ALOX15. The activity of ALOX15 in indicated stable cells was measured using the Lipoxygenase (LOX) Activity Assay Kit. E-F) *in vitro* kinase assay further confirmed that PKCβ directly phosphorylates wildtype ALOX15 but not ALOX15^T537A^ (E) mutant or ALOX15^T560A^ (F). G) The phosphorylated ALOX15^T537^ and phosphorylated ALOX15^T560^ are only detected in ferroptosis but not in pyroptosis or necroptosis. HT1080 cells were treated with RSL3 (1 μΜ, for 8h) to induce ferroptosis. GSDME overexpressed B16-F10 cells were treated with raptinal (30 μΜ, for 10h) to induce pyroptosis. L-929 cells were treated with T/S/Z (TNF-α, Smac mimetics and Z-VAD-FMK, for 4h) to induce necroptosis. Western images confirmed the expression of indicated markers. TNF-α: 20 ng/mL, Smac Mimetic: 10 µM, Z-VAD-FMK: 20 µM.

Data are derived from three independent experiments, and each value represents the mean ± SD. **P < 0.05, **P < 0.01, ***P < 0.001*, *t* test.

**
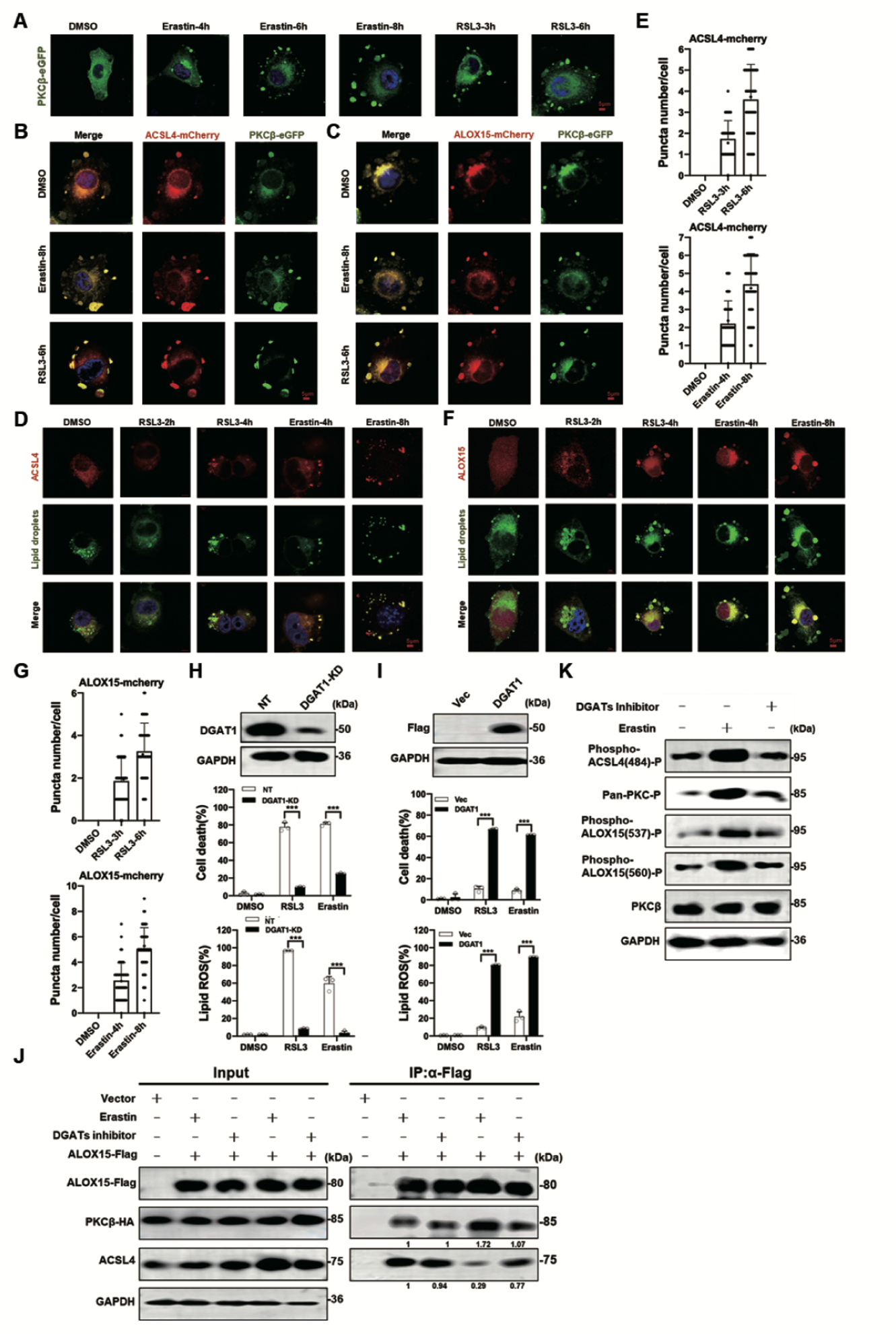

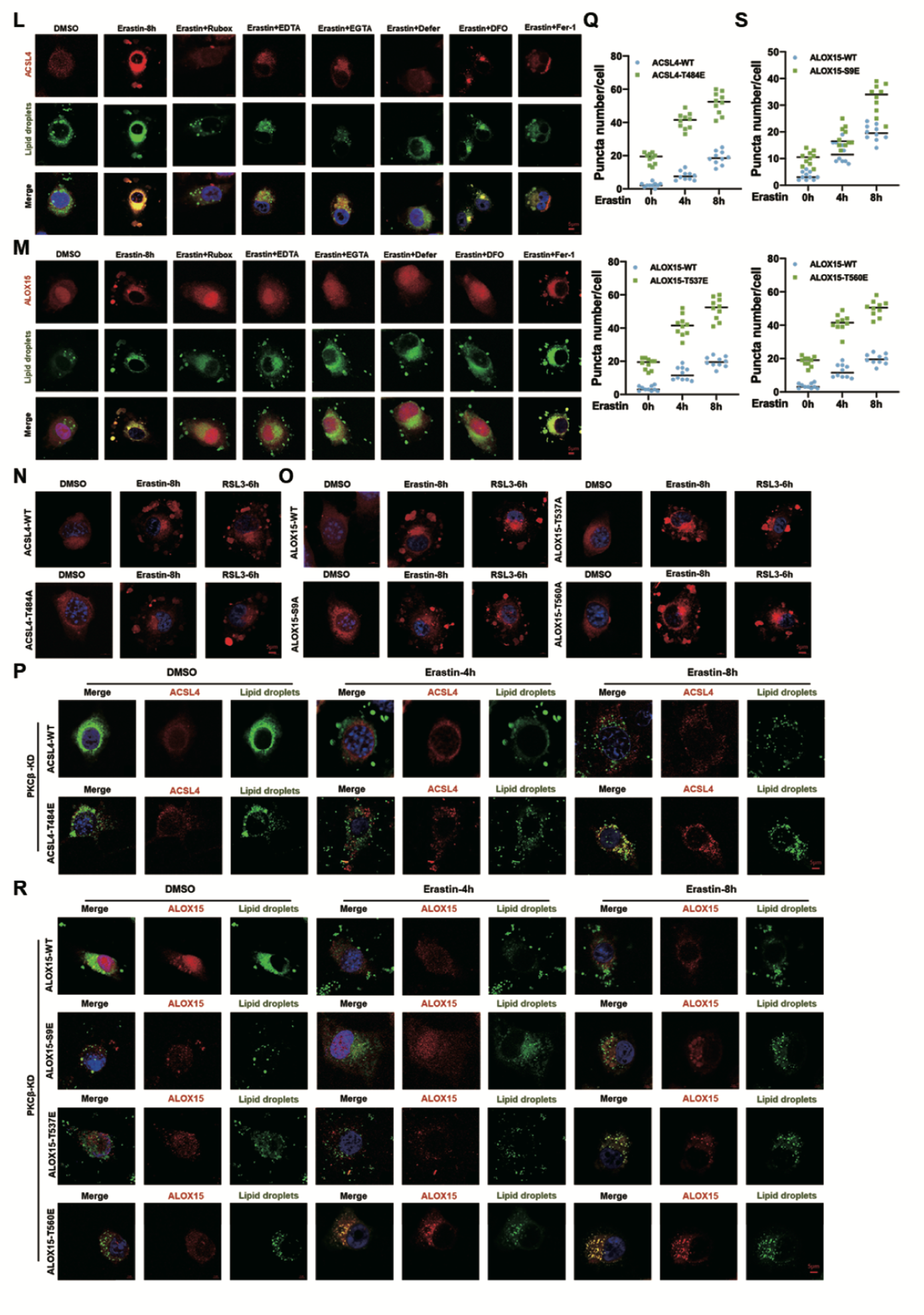
 Figure S6 The PKC**β**-ACSL4-ALOX15 complex translocate to lipid droplets during ferroptosis**

A) Erastin (10 μΜ) or RSL3 (10 μΜ) induces PKCβ puncta formation in a time dependent manner. PKCβ-eGFP labelled HT1080 cells were treated as indicated, cells were fixed and imaged with Confocal Microscopy. Scale bar: 5µM. B) Erastin (10 μΜ, for 8 h) or RSL3(1 μΜ, for 6 h) induces ACSL4-PKCβ co-localized puncta formation. HT1080 cells labelled with PKCβ-eGFP and ACSL4-mCherry were treated as indicated, cells were fixed and imaged with Confocal Microscopy. Scale bar: 5µM. C) Erastin (10 μΜ, for 8 h) or RSL3(1 μΜ, for 6 h) induces ALOX15-PKCβ co-localized puncta formation. HT1080 cells labelled with PKCβ-eGFP and ALOX15-mCherry were treated as indicated, cells were fixed and imaged with Confocal Microscopy. Scale bar: 5µM. D) Erastin (10 μΜ, for 8 h) or RSL3(1 μΜ, for 6 h) induces lipid droplets localized ACSL4 puncta formation in a time dependent manner. ACSL4-mCherry labelled HT1080 cells were treated as indicated, cells were fixed and imaged with Confocal Microscopy. Scale bar: 5µM. E) Quantitative bar shows the average number of ACSL4-mCherry puncta per cell from Figure S6D, 50 cells were counted. F) Erastin (10 μΜ, for 8 h) or RSL3(1 μΜ, for 6 h) induced lipid droplets localized ALOX15 puncta formation in a time dependent manner. ALOX15-mCherry labelled HT1080 cells were treated as indicated, cells were fixed and imaged with Confocal Microscopy. Scale bar: 5µM. G) Quantitative bar shows the average number of ALOX15-mCherry puncta per cell from Figure S6F, 50 cells were counted. H) Knockdown of DGAT1 significantly suppressed erastin or RSL3 induced ferroptosis. HT1080 cells as indicated were treated with RSL3 (1 μΜ, for 10 h) or erastin (10 μΜ, for 20 h), then cell death and lipid ROS were measured as described in Figure 1b. I) overexpression of DGAT1 promoted erastin or RSL3 induced ferroptosis. HT1080 cells as indicated were treated with RSL3 (1 μΜ, for 10 h) or erastin (10 μΜ, for 20 h), then cell death and lipid ROS were measured as described in Figure 1b. J) DGATs inhibitors treatment rescued the ACSL4-PKCβ-ALOX15 complex dynamic change induced by erastin treatment. K) The DGATs inhibitors treatment blocked erastin induced PKCβ activation and the phosphorylation of PKCβ downstream substrates ACSL4 and ALOX15. L) The effect of ferroptosis inhibitors on erastin induced ACSL4 puncta formation and its lipid droplets translocation. ACSL4-mCherry labelled HT1080 cells were treated as indicated, and stained with BODIPY for lipid droplets, then imaged with Confocal Microscopy. Erastin :10 μΜ, EDTA: 40 μΜ, EGTA: 50 μΜ, Rubox: 5 μΜ, Defer: 50 μΜ, Fer-1: 100 μΜ. Scale bar: 5µM. M) The effect of ferroptosis inhibitors on erastin induced ALOX15 puncta formation and its lipid droplets translocation. ALOX15-mCherry labelled HT1080 cells were treated as indicated, and stained with BODIPY for lipid droplets, then imaged with Confocal Microscopy. Erastin :10 μΜ, EDTA: 40 μΜ, EGTA: 50 μΜ, Rubox: 5 μΜ, Defer: 50 μΜ, Fer-1: 100 μΜ. Scale bar: 5µM. N) The ACSL4^T484A^ loss-of-function mutant does not affect ACSL4 puncta formation induced by erastin (10 μΜ, for 8 h) or RSL3(1 μΜ, for 6 h). HT1080 cells labelled ACSL4-WT-mCherry or ACSL4-T484A-mCherry were treated as indicated, cells were fixed and imaged with Confocal Microscopy. Scale bar: 5µM. O) The ALOX15^S9A^, ALOX15^T537A^ or ALOX15^T560A^ loss-of-function mutant do not affect erastin (10 μΜ, for 8 h) or RSL3 (1 μΜ, for 6 h) induced ALOX15 puncta formation. HT1080 cells labelled with ALOX15-WT-mCherry, ALOX15-S9A-mCherry, ALOX15-T537A-mCherry or ALOX15-T560A-mCherry were treated as indicated, cells were fixed and imaged with Confocal Microscopy. Scale bar: 5µM. P) The ACSL4^T484E^ mutant phosphorylation mimic mutant promotes lipid droplet localized ACSL4 puncta formation induced by erastin. PKCβ-KD HT1080 cells expressed with ACSL4-WT-Flag or ACSL4-T484E-Flag were treated with erastin (10µM) as indicated, and the cells were stained with lipid droplet dye BODIPY™ 493/503. Then the cells were subjected to immunofluorescence assay with Flag primary antibody and 594 nm red fluorescent secondary antibody, and imaged with Confocal Microscopy. Scale bar: 5µM. Q) Quantitative bar shows the average number of ACSL4-mCherry puncta per cell from Figure S6P, 50 cells were counted. R) The ALOX15^S9E^, ALOX15^T537E^ or ALOX15^T560E^ phosphorylation mimic mutant promotes lipid droplet localized ALOX15 puncta formation induced by erastin. PKCβ-KD HT1080 cells expressed with ALOX15-WT-Flag, ALOX15^S9E^, ALOX15^T537E^ or ALOX15^T560E^ were treated with erastin (10µM) as indicated, and the cells were stained with lipid droplet dye BODIPY™ 493/503. Then the cells were subjected to immunofluorescence assay with Flag primary antibody and 594 nm red fluorescent secondary antibody, and imaged with Confocal Microscopy. Scale bar: 5µM. S) Quantitative bar shows the average number of ALOX15-mCherry puncta per cell from Figure S6R, 50 cells were counted.

Data are derived from three independent experiments, and each value represents the mean ± SD. **P < 0.05, **P < 0.01, ***P < 0.001*, *t* test.

**Sequence of primers in this study**

CaV1.1-F GACCACTCAGAGCCATCAACAG

CaV1.1-R GGTAGTGACCAGCACGATGTTC

CaV1.2-F GCAGGAGTACAAGAACTGTGAGC

CaV1.2-R CGAAGTAGGTGGAGTTGACCAC

CaV1.3-F CTTCGACAACGTCCTCTCTGCT

CaV1.3-R GCCGATGTTCTCTCCATTCGAG

CaV1.4-F GGAAAGACACCACCCCAGAG

CaV1.4-R ACTGCCACTGTCTTGTGCTT

CaV2.1-F CTGGTAGCCTTTGCCTTCACTG

CaV2.1-R ACACAGCCTTGAGCTTTGGCAG

CaV2.2-F TGGACCTGGAAAGCCAAGCAGA

CaV2.2-R GGTCACGATGTAGTGGCAGAAG

CaV2.3-F AGCGTGAGACAGGCAAAGCCAT

CaV2.3-R GGATGCACATCTCAAAGTAGCGC

CaV3.1-F TTCACCGCAGTCTTTCTGGCTG

CaV3.1-R TGACGGAGATGAGCACCAACAG

CaV3.2-F GGAACATCTCCACCAAGGCACA

CaV3.2-R TCCATCCTTGGATGACAGCACG

CaV3.3-F GTGTCCAACTACATCTTCACGGC

CaV3.3-R GACAAGAAAGCCATCCAGCACG

NMDA-F GAACATCCGCAGCAACTATCCC

NMDA-R GCAGCATCGTAGATGAAGGCGT

hACTIN-F CATGTACGTTGCTATCCAGGC

hACTIN-R CTCCTTAATGTCACGCACGAT

TNF-α-F GACCCTCACACTCAGATCATCTTC,

TNF-α-R TGCTACGACGTGGGCTACA,

IL-1β-F TCGCTCAGGGTCACAAGAAA,

IL-1β-R ATCAGAGGCA GGGAAACAC,

mACTIN-F TGACGTTGACATCCGTAAAGA

mACTIN-R GCCGGACTCATCGTACTCC
