# Supplementary material for: Inhibition of Calcium‐Dependent Lipid Droplets Relocation of ACSL4‐PKCβ‐ALOX15 Complex Alleviates Ferroptosis and Acute Pancreatitis
Source: Adv Sci (Weinh). 2026 Jan 27;13(19):e15768. doi: 10.1002/advs.202515768 (PMC13045411; doi:10.1002/advs.202515768)
Supplement: Supplementary file 2 — Supporting File 2: advs74009‐sup‐0002‐Data.pdf. [file ADVS-13-e15768-s001.pdf]

Figure 1E

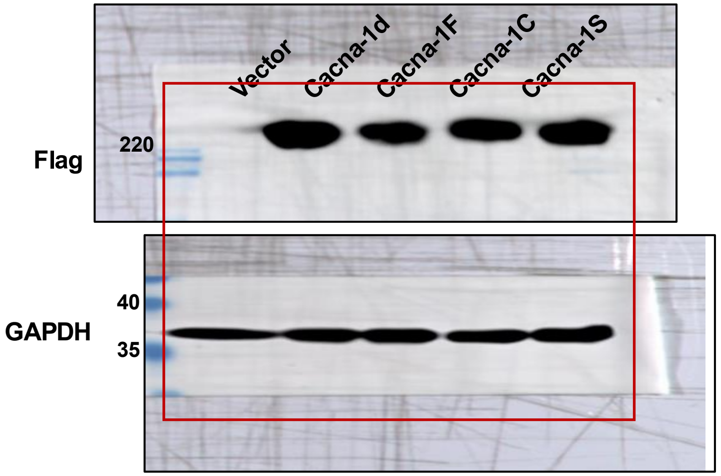

Figure 2B

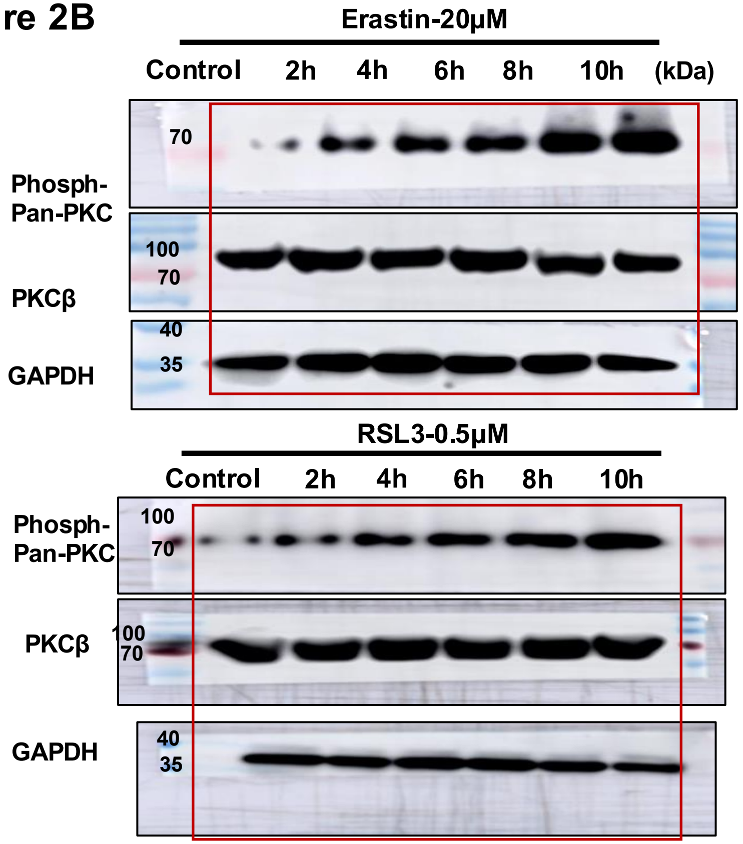

Figure 2C

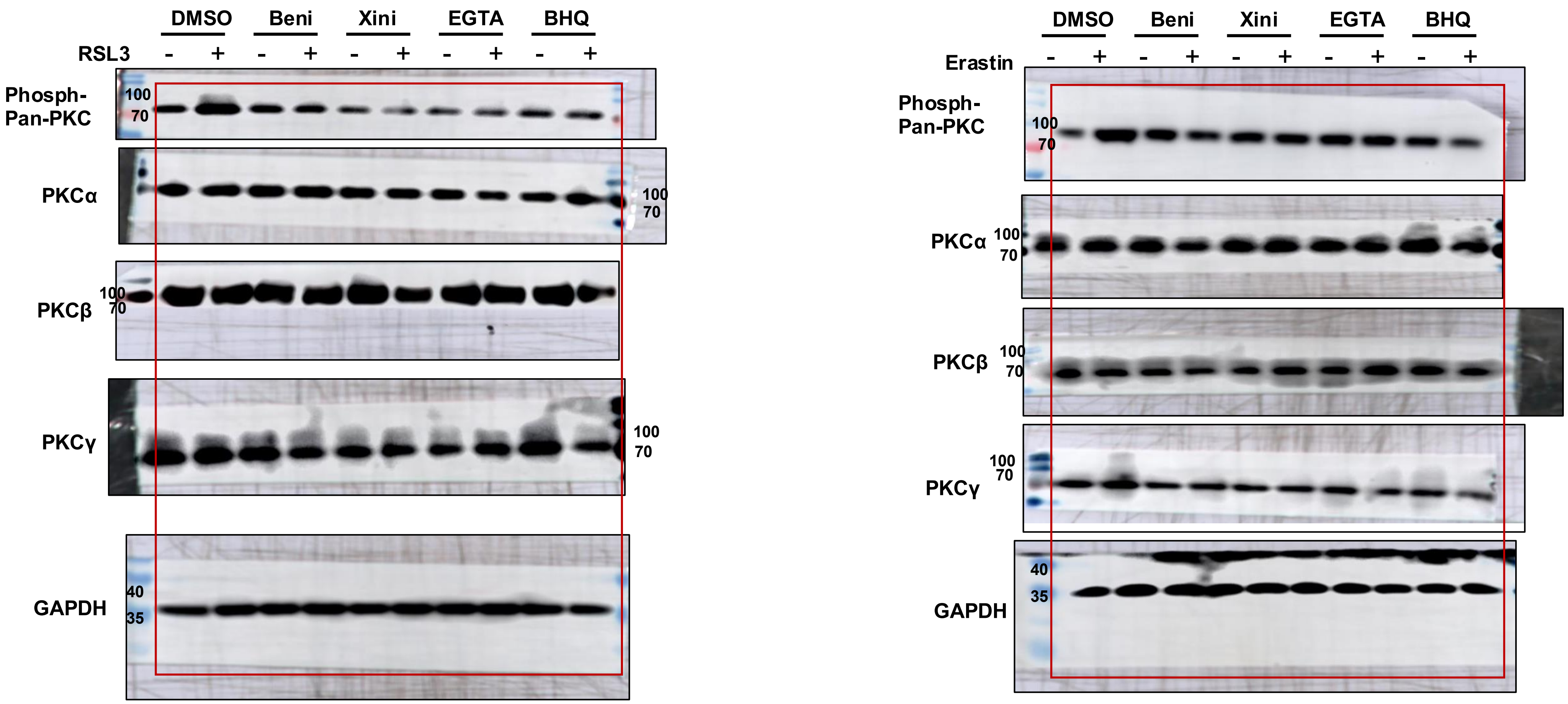
DMSO
Beni
Xini
EGTA
BHQ
RSL3
- + - + - + - + - +
Phosph-Pan-PKC
PKCα
PKCβ
PKCγ
GAPDH
100
70
40
35
DMSO
Beni
Xini
EGTA
BHQ
Erastin
- + - + - + - + - +
Phosph-Pan-PKC
PKCα
PKCβ
PKCγ
GAPDH

Figure 2D

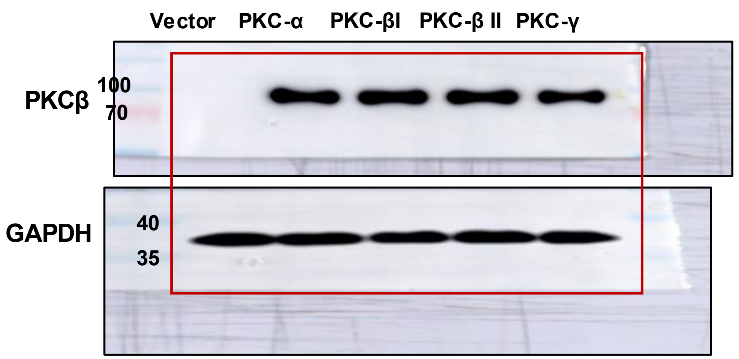

Figure 2F

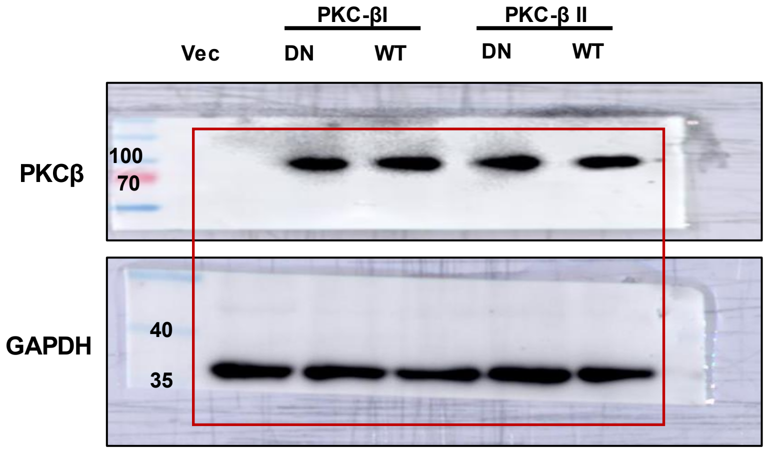

Figure 2G

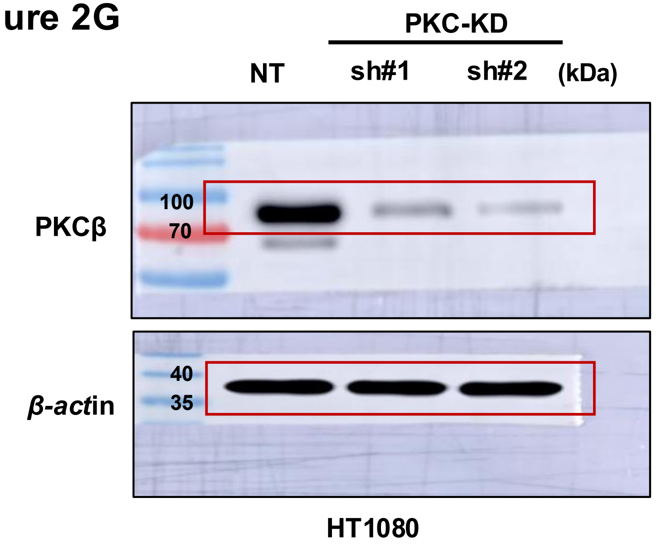

Figure 2H

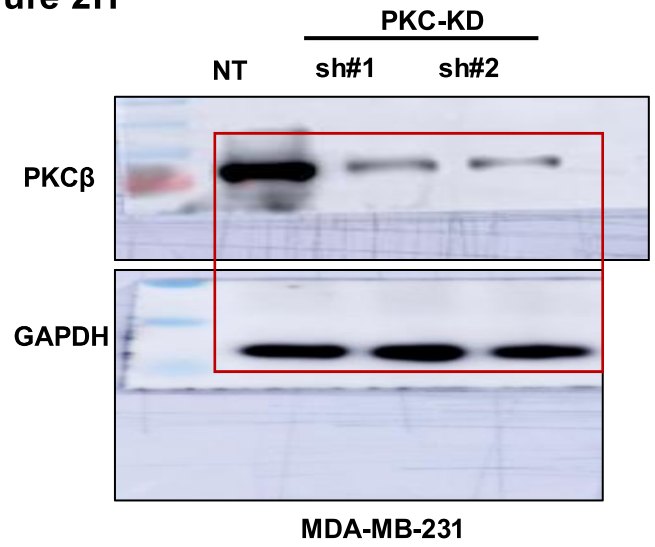

Figure S2A

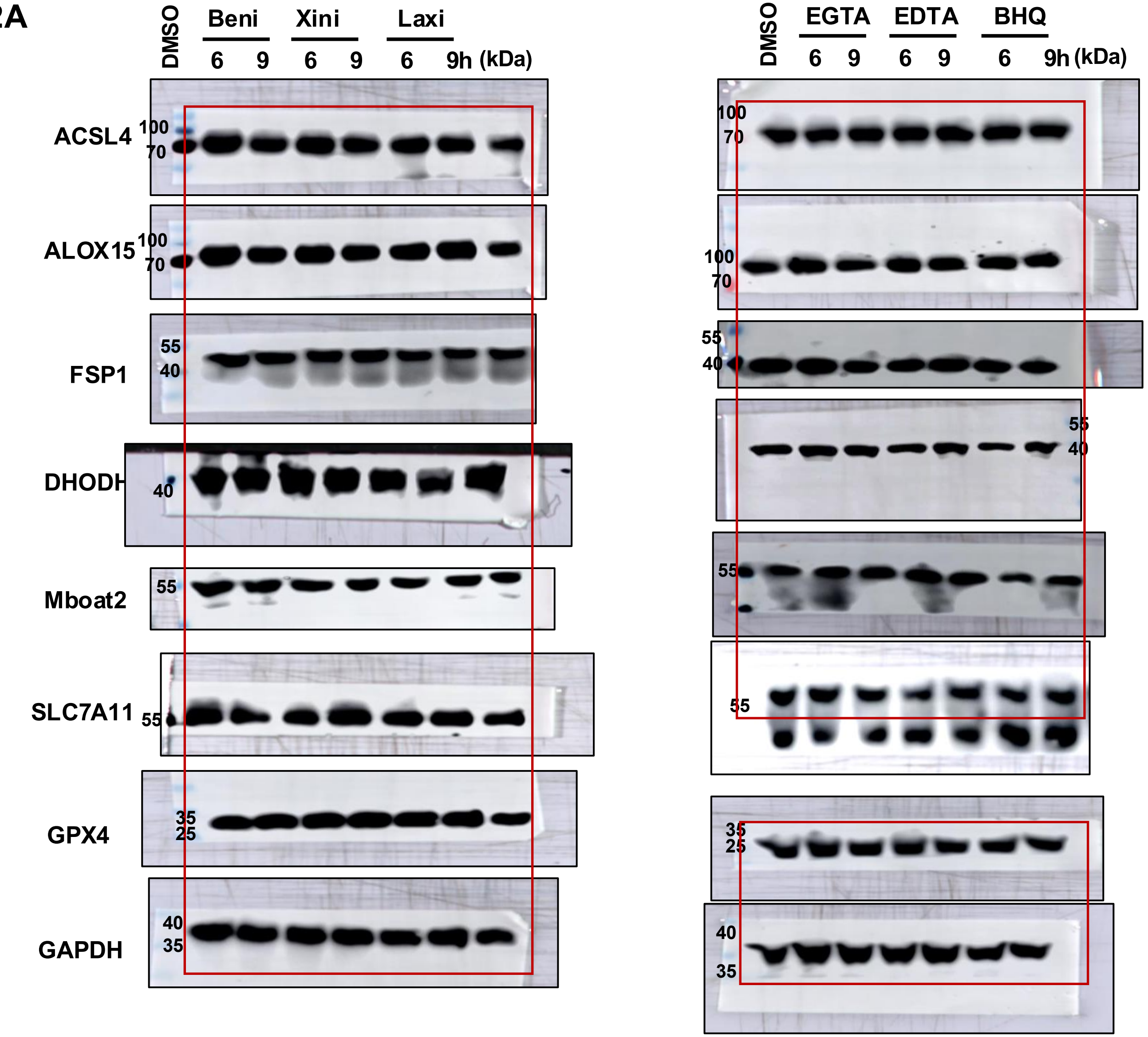

Figure S2G

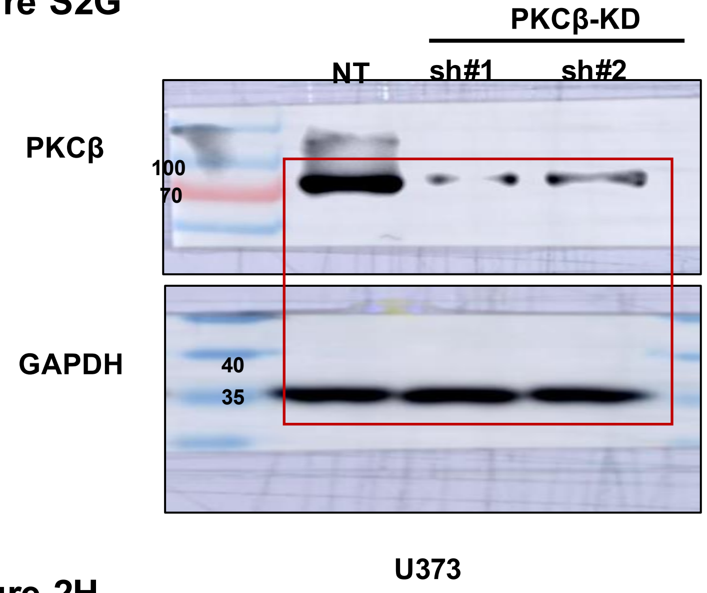

Figure 2H

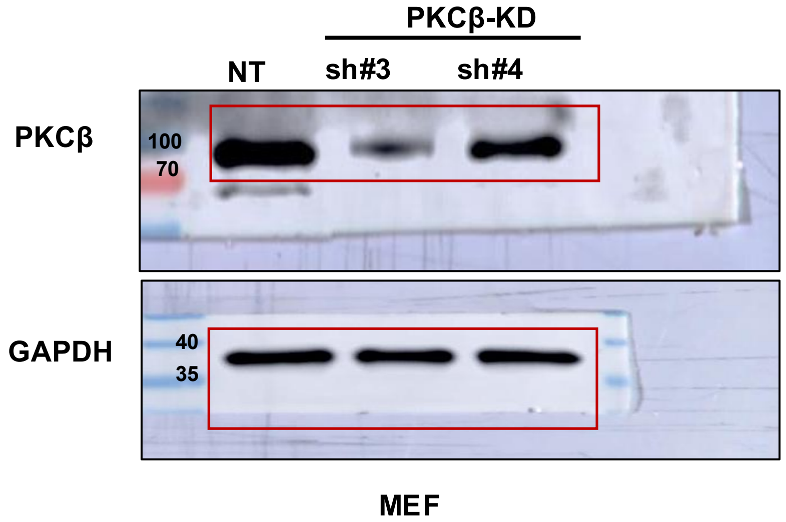

Figure 3A

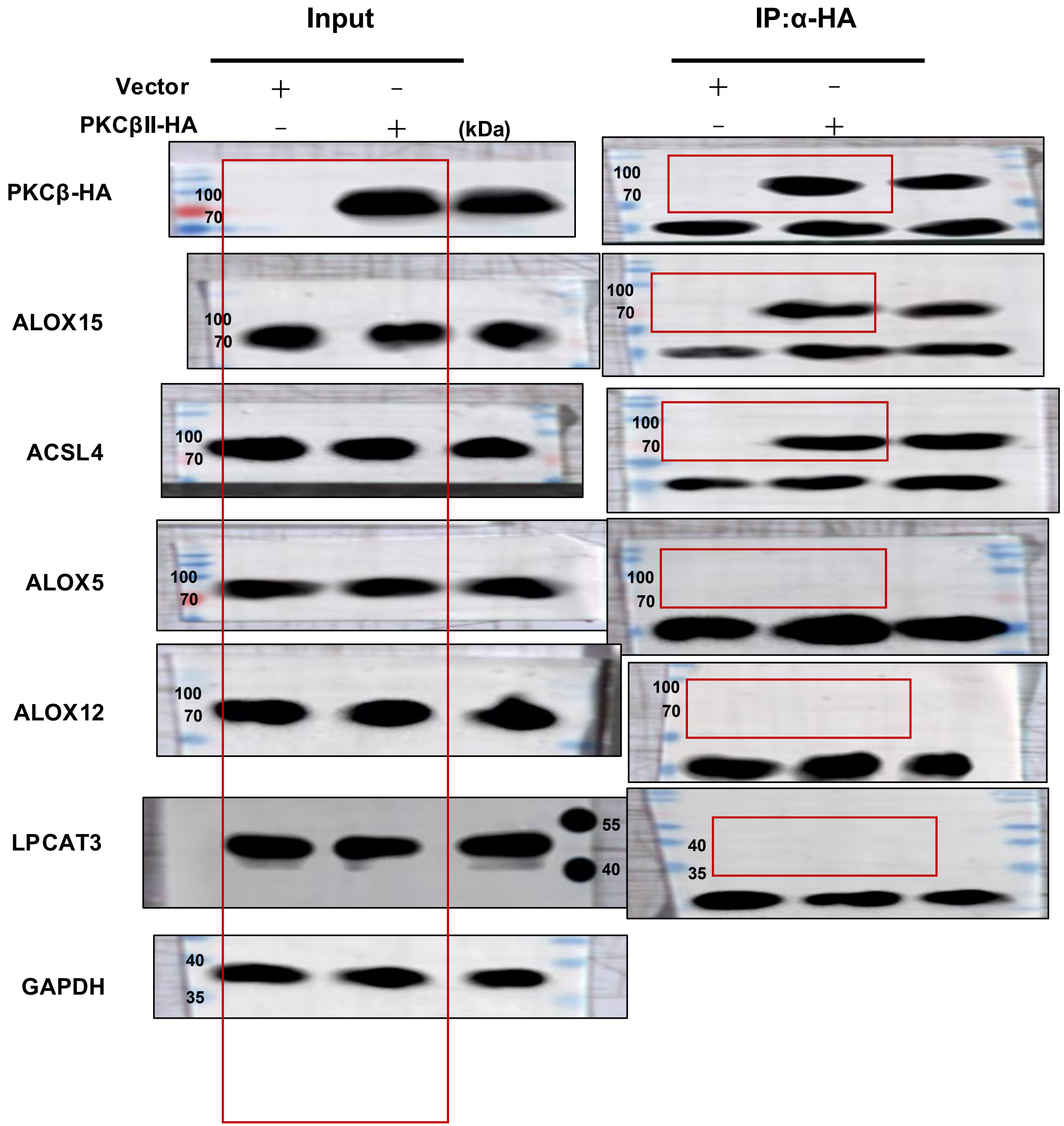
Input
IP:α-HA
Vector
PKCβII-HA
(kDa)
PKCβ-HA
ALOX15
ACSL4
ALOX5
ALOX12
LPCAT3
GAPDH
100
70
55
40
35

Figure 3B

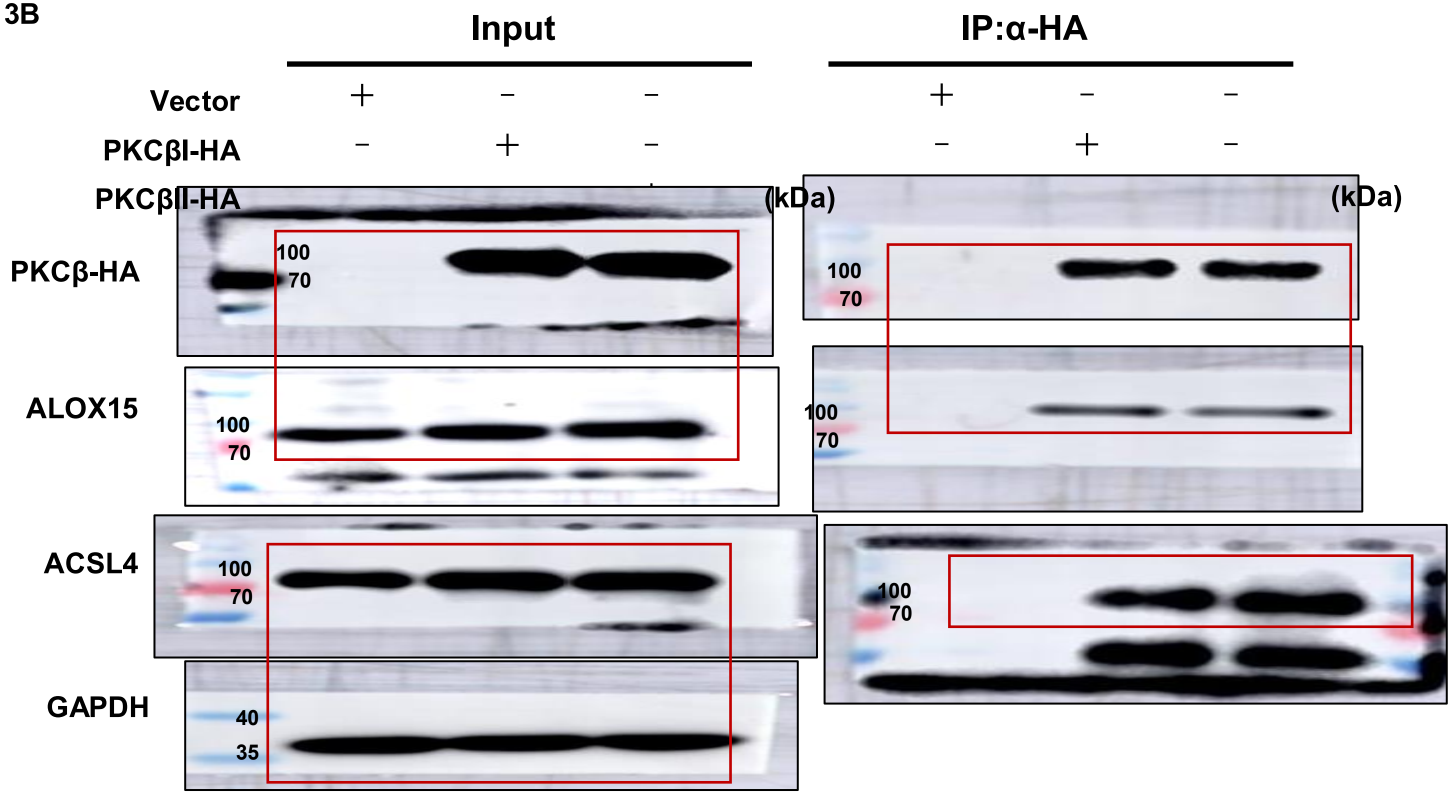

Figure 3C

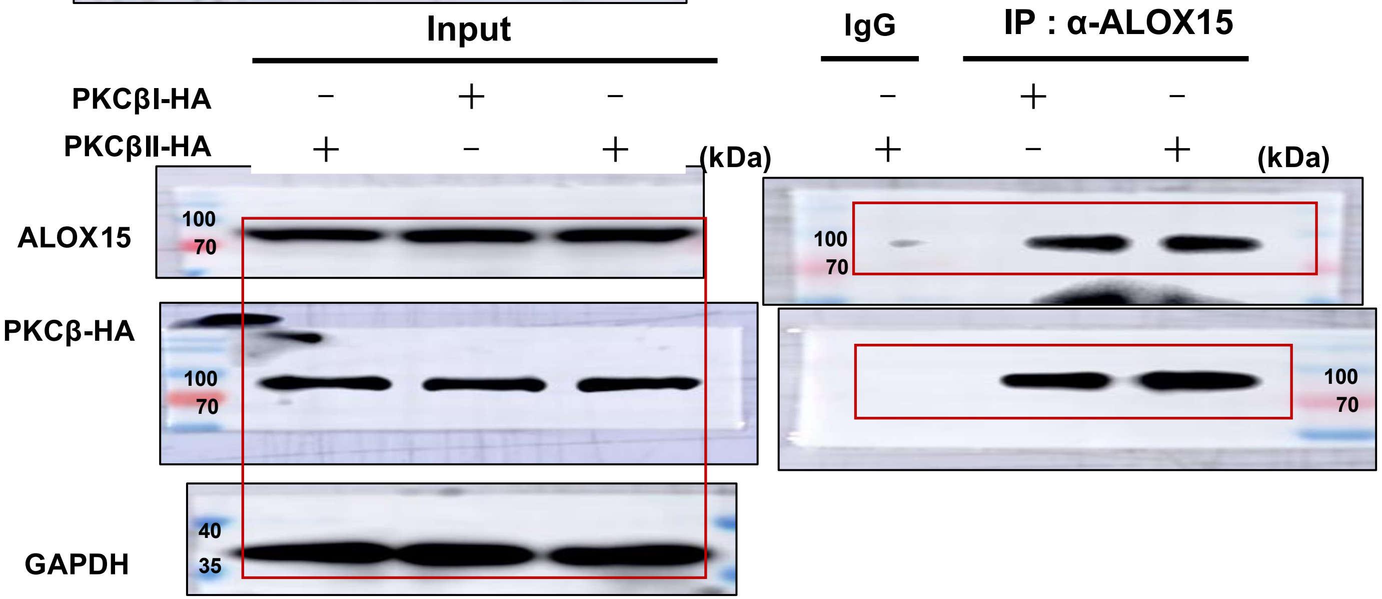

Figure 3D

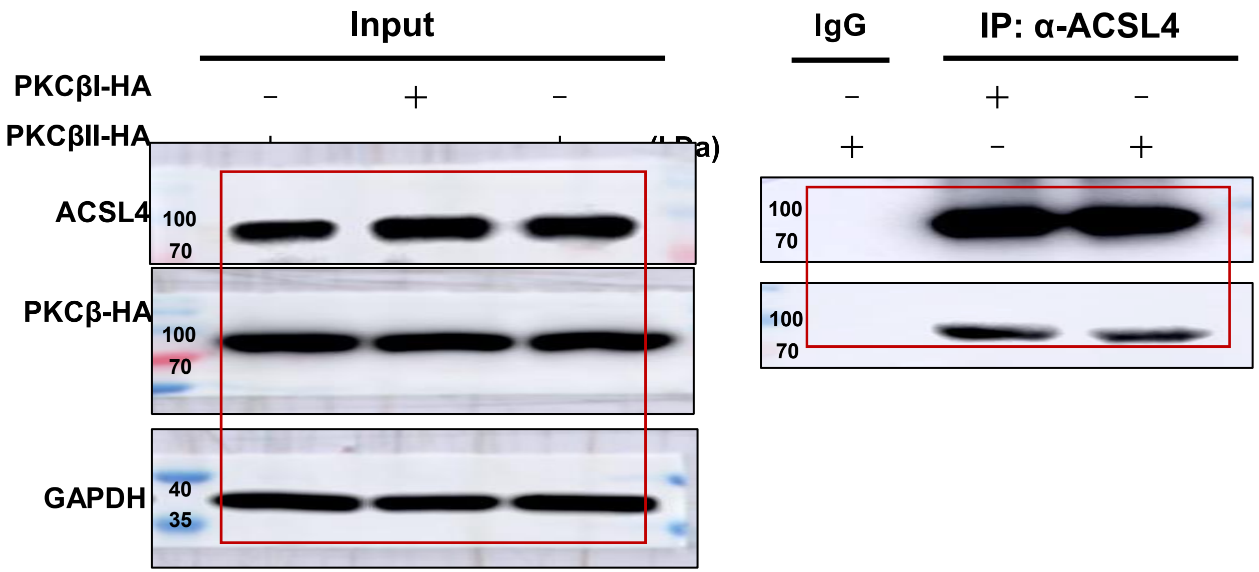

Figure 3E

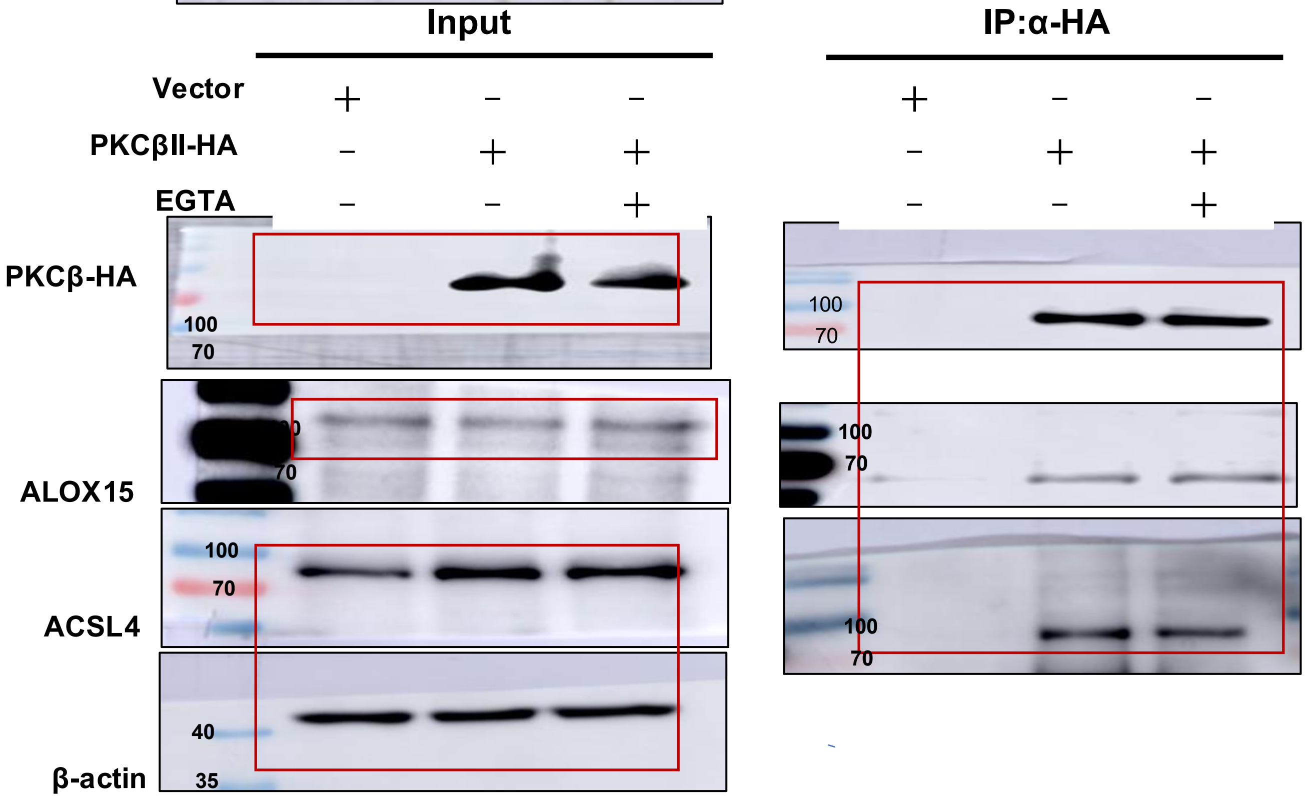

Figure 3F

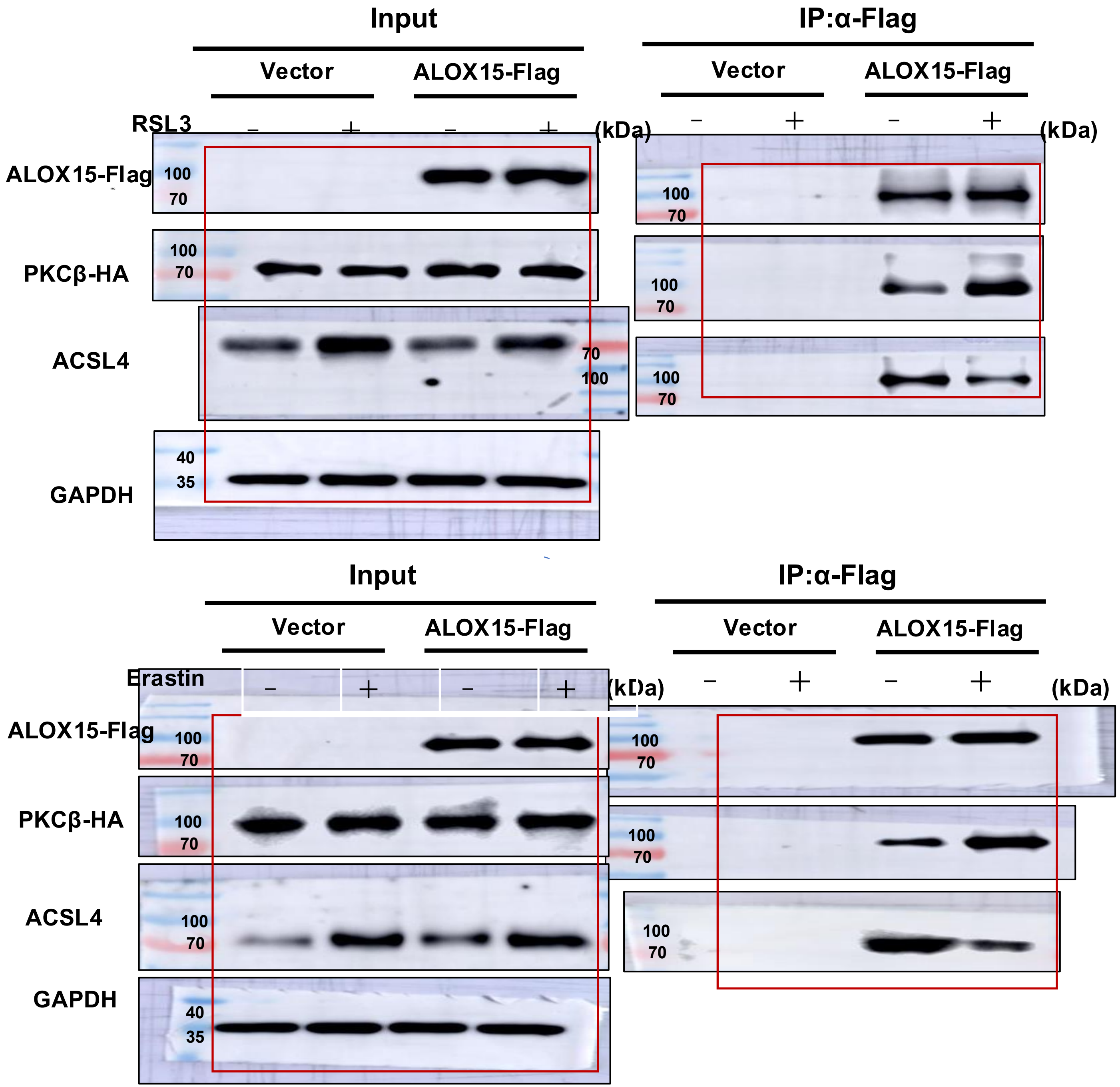
Input
IP:α-Flag
Vector
ALOX15-Flag
RSL3
(kDa)
ALOX15-Flag
PKCβ-HA
ACSL4
GAPDH
100
70
40
35
Erastin

Figure 3G

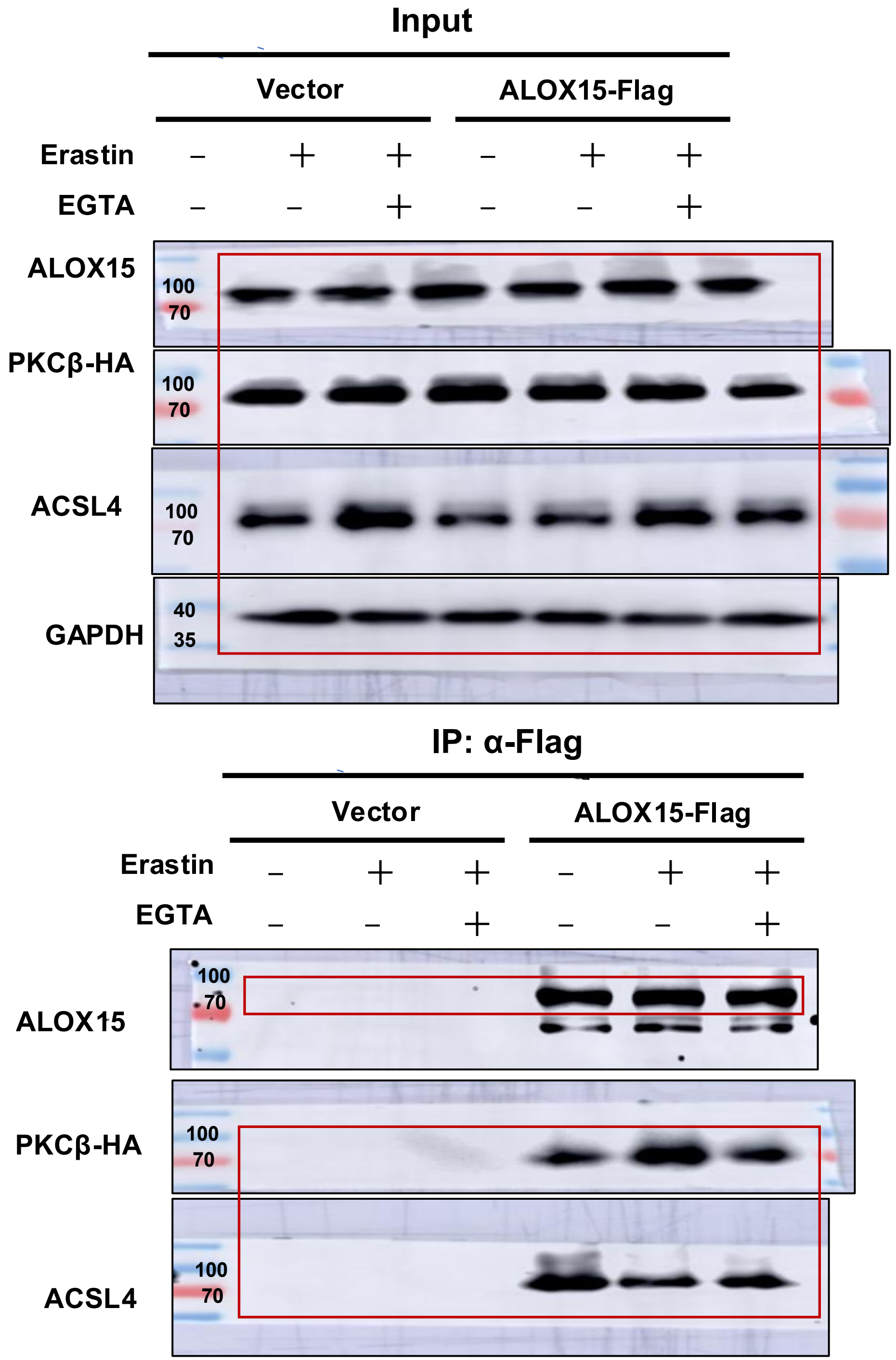
Input
Vector
ALOX15-Flag
Erastin
EGTA
ALOX15
PKCβ-HA
ACSL4
GAPDH
100
70
40
35
IP: α-Flag
Vector
ALOX15-Flag
Erastin
EGTA
ALOX15
PKCβ-HA
ACSL4

Figure 3H

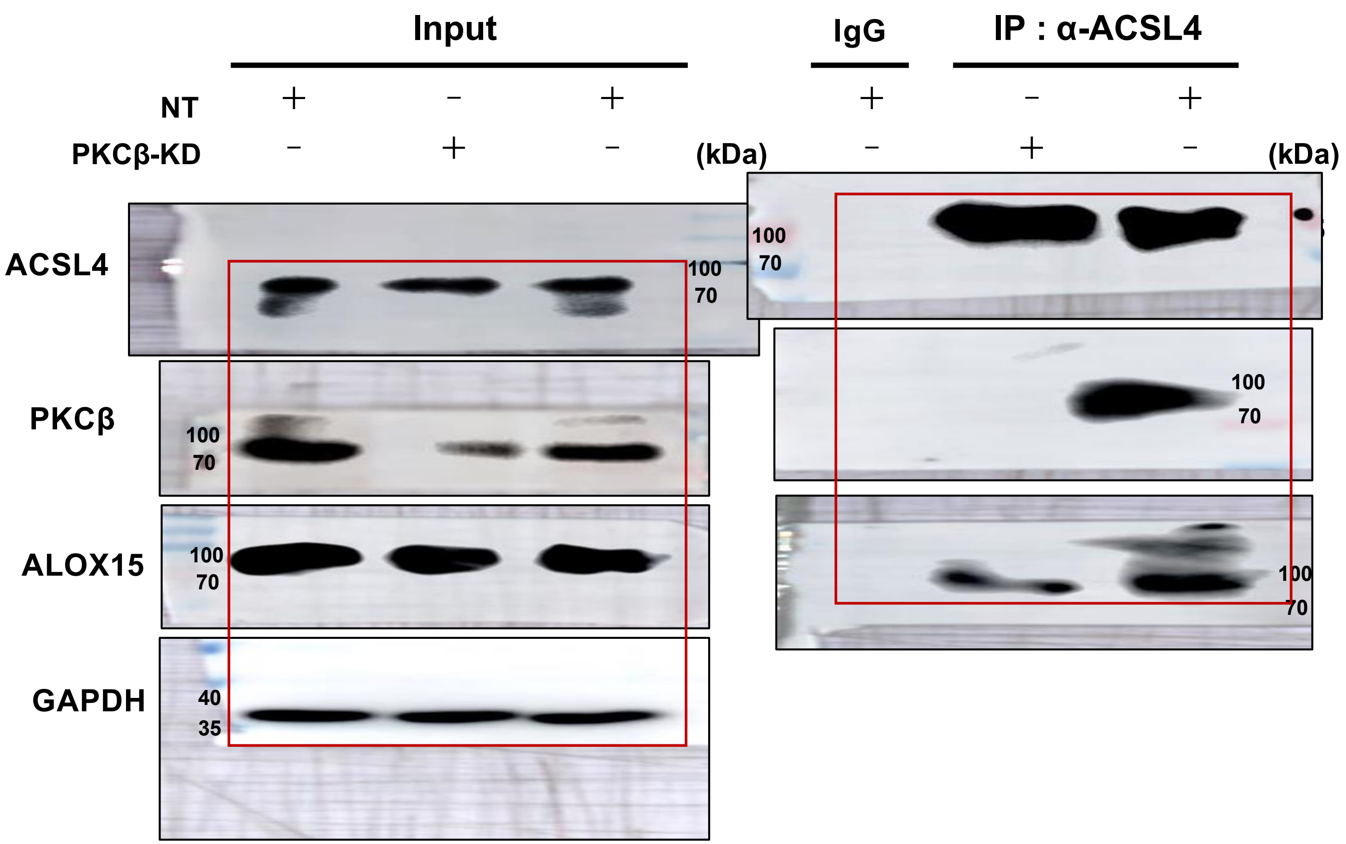

Input
IgG
IP : α-ACSL4
NT
PKCβ-KD
(kDa)
(kDa)
ACSL4
PKCβ
ALOX15
GAPDH
100
70
40
35

Figure 3I

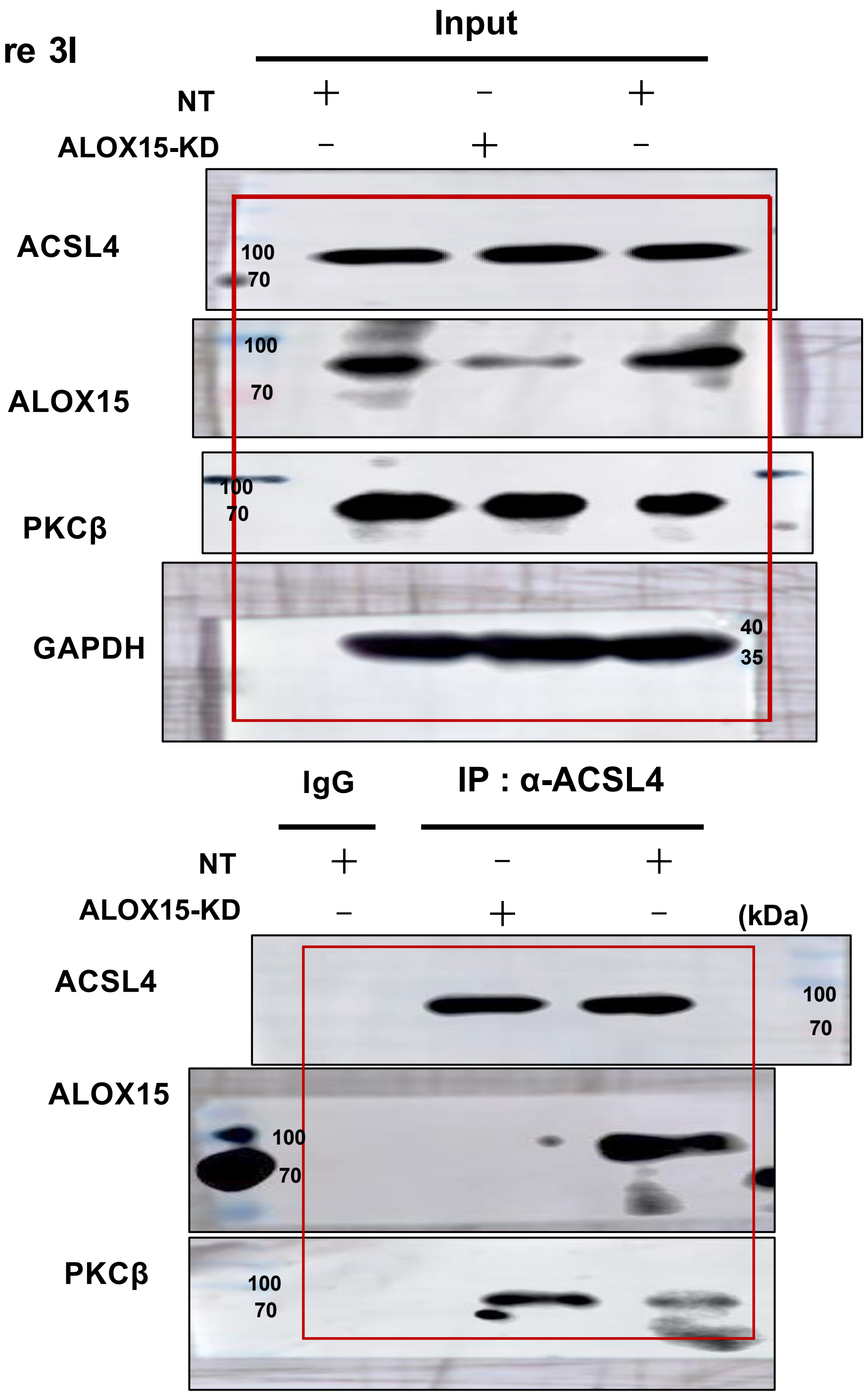

Figure 3J

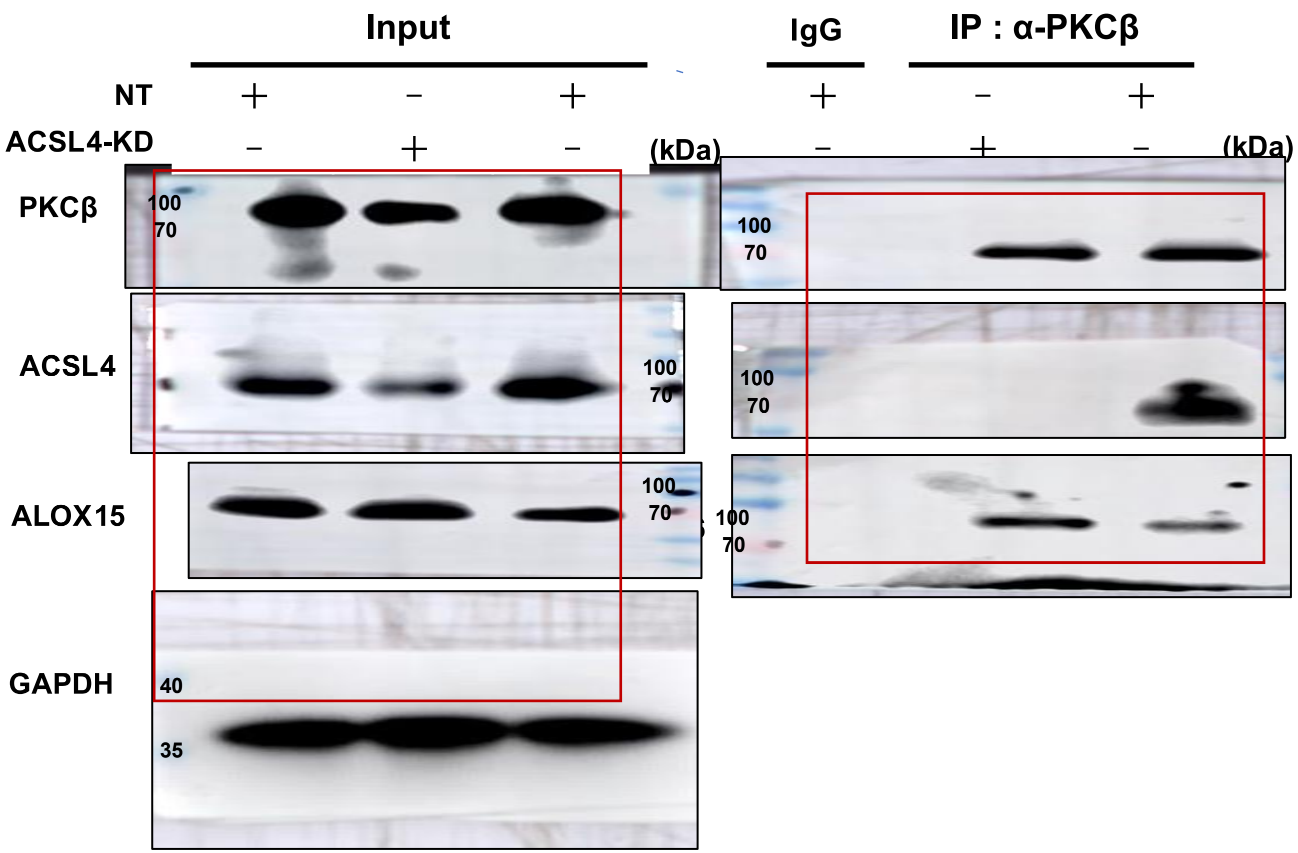

Figure S3A

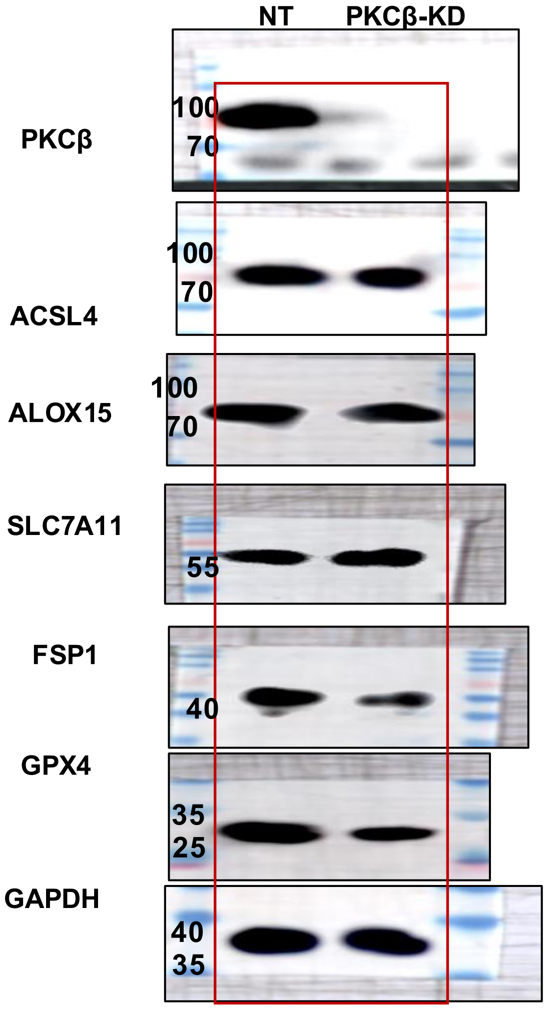

Figure S3B

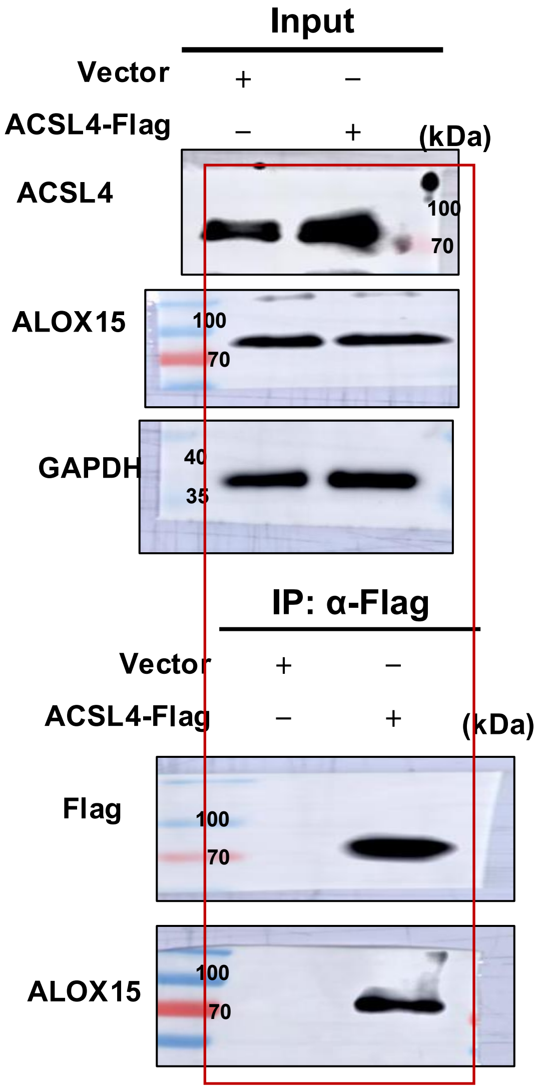

Figure S3C

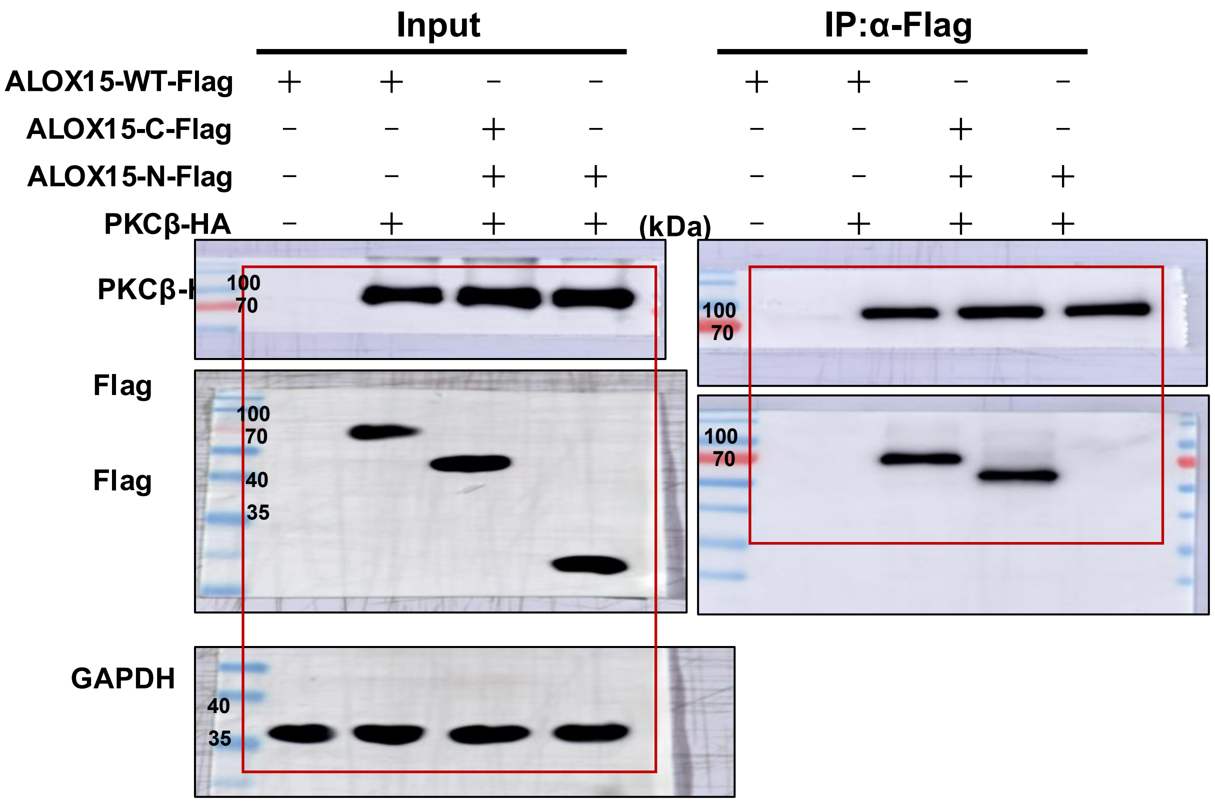

Figure S3D

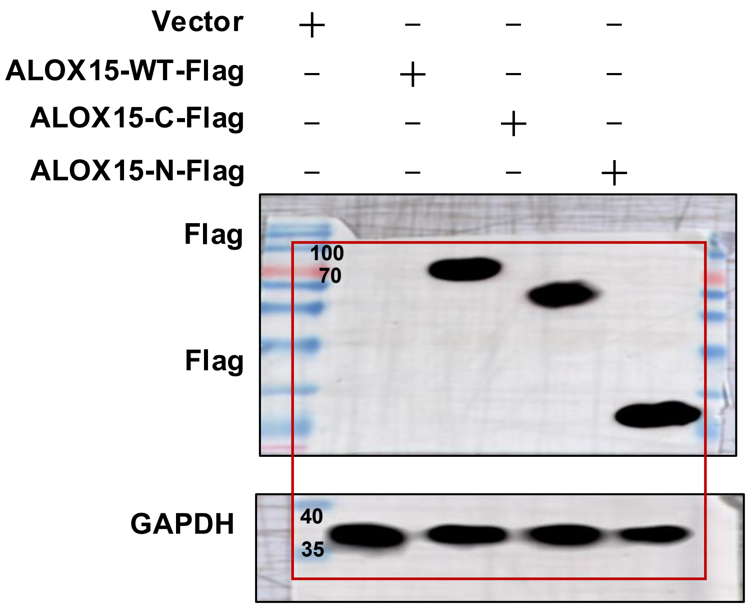

Figure S3F

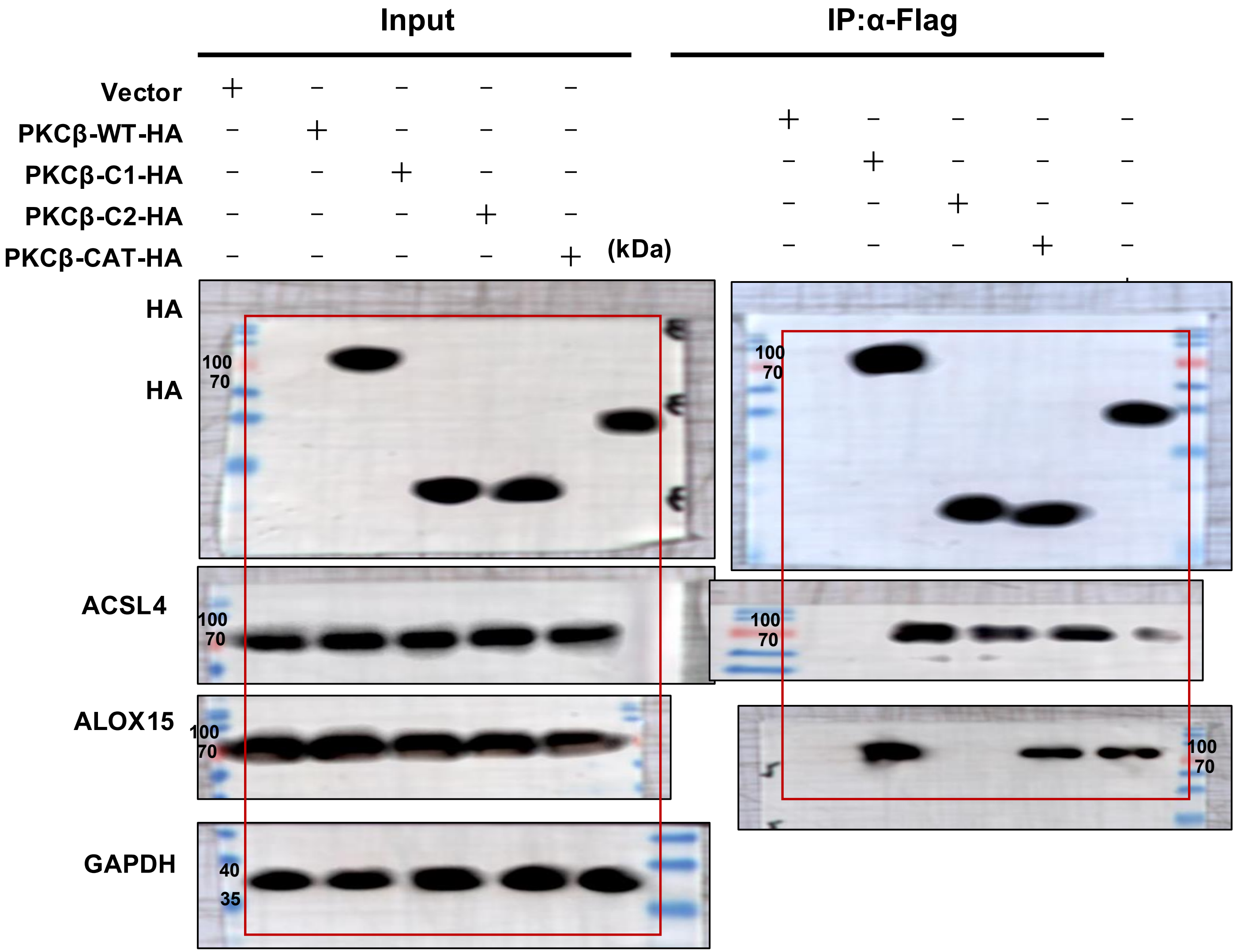
Input
IP:α-Flag
Vector
PKCβ-WT-HA
PKCβ-C1-HA
PKCβ-C2-HA
PKCβ-CAT-HA
(kDa)
HA
HA
ACSL4
ALOX15
GAPDH
100
70
40
35

Figure 4A

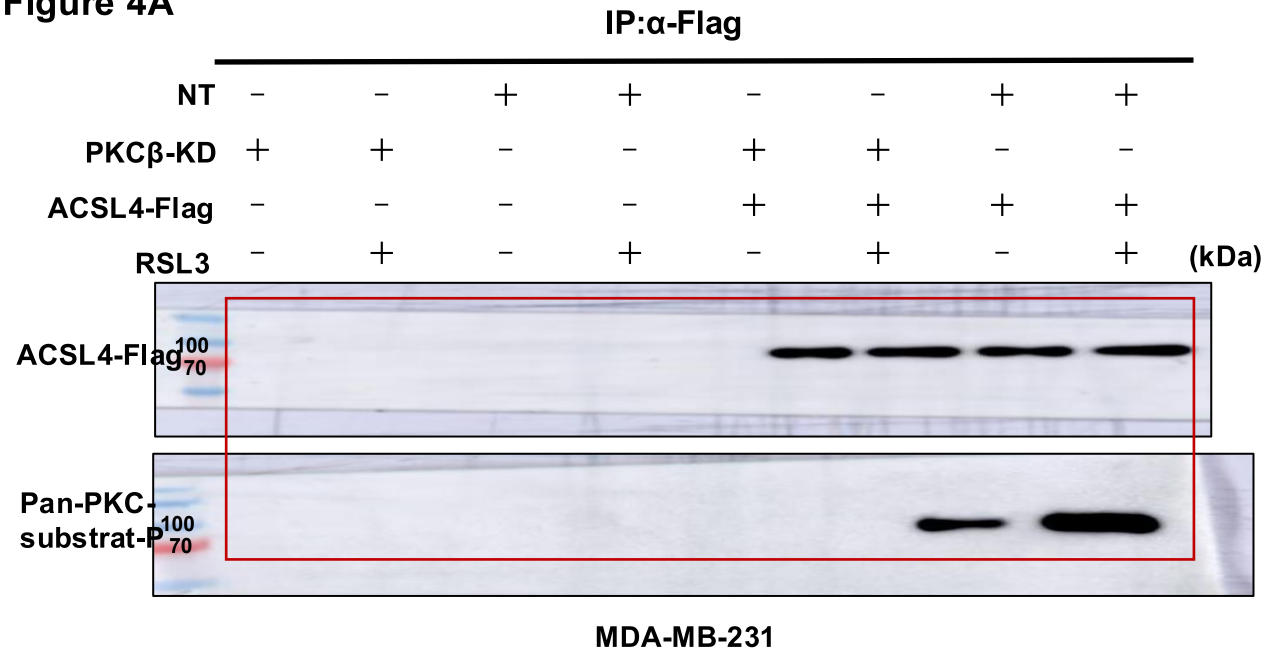

Figure 4B

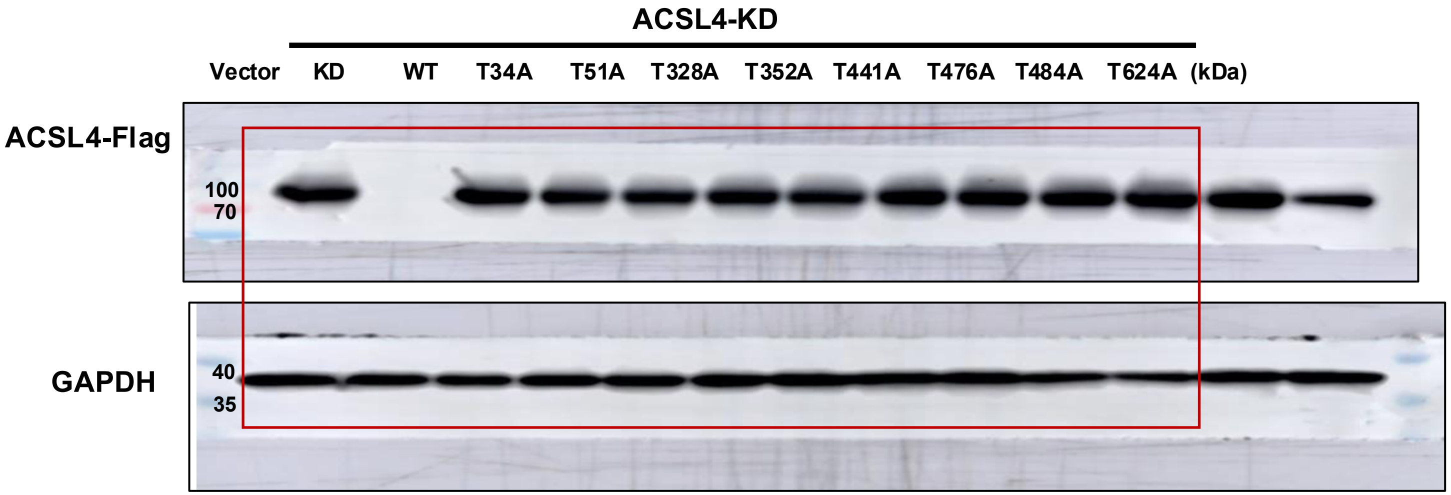

Figure 4E

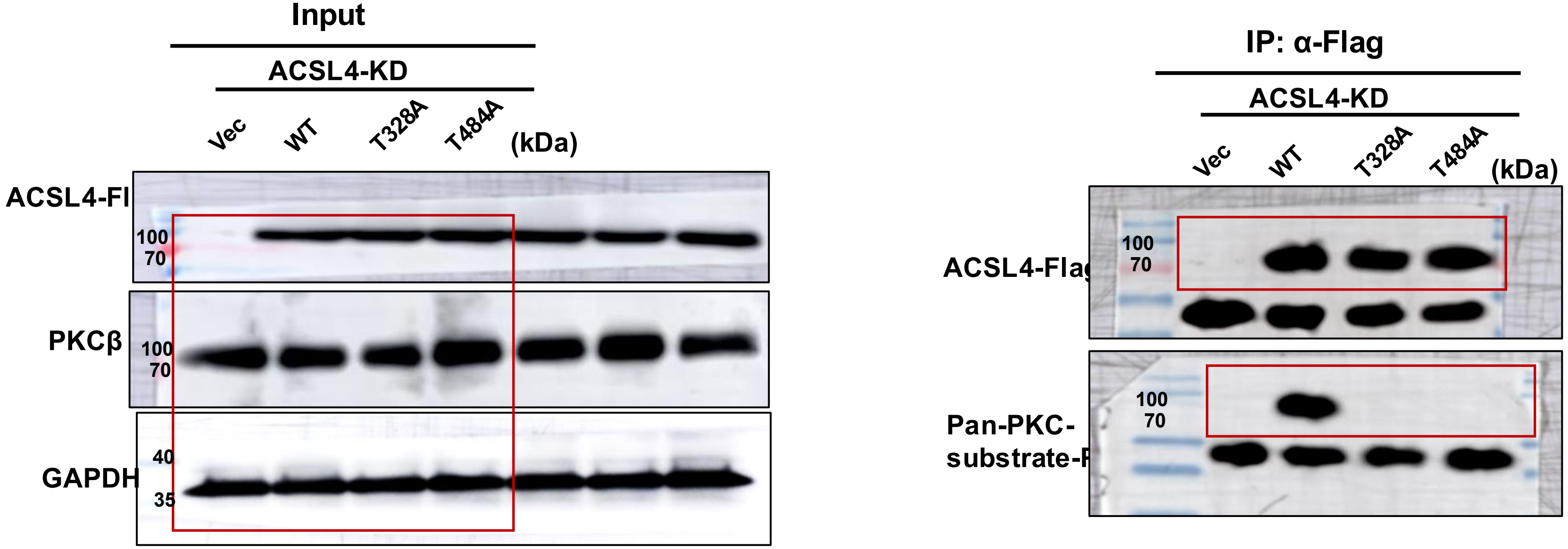

Figure 4F

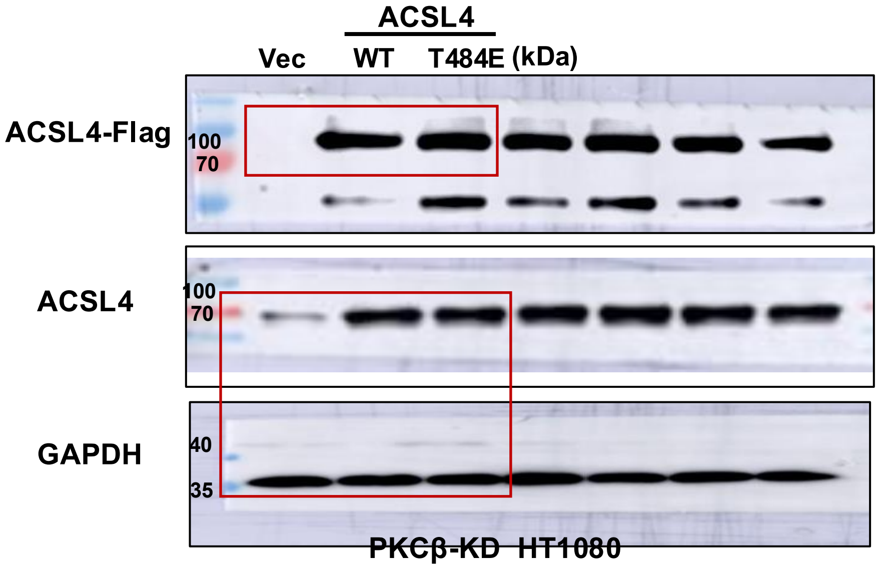

Figure 4G

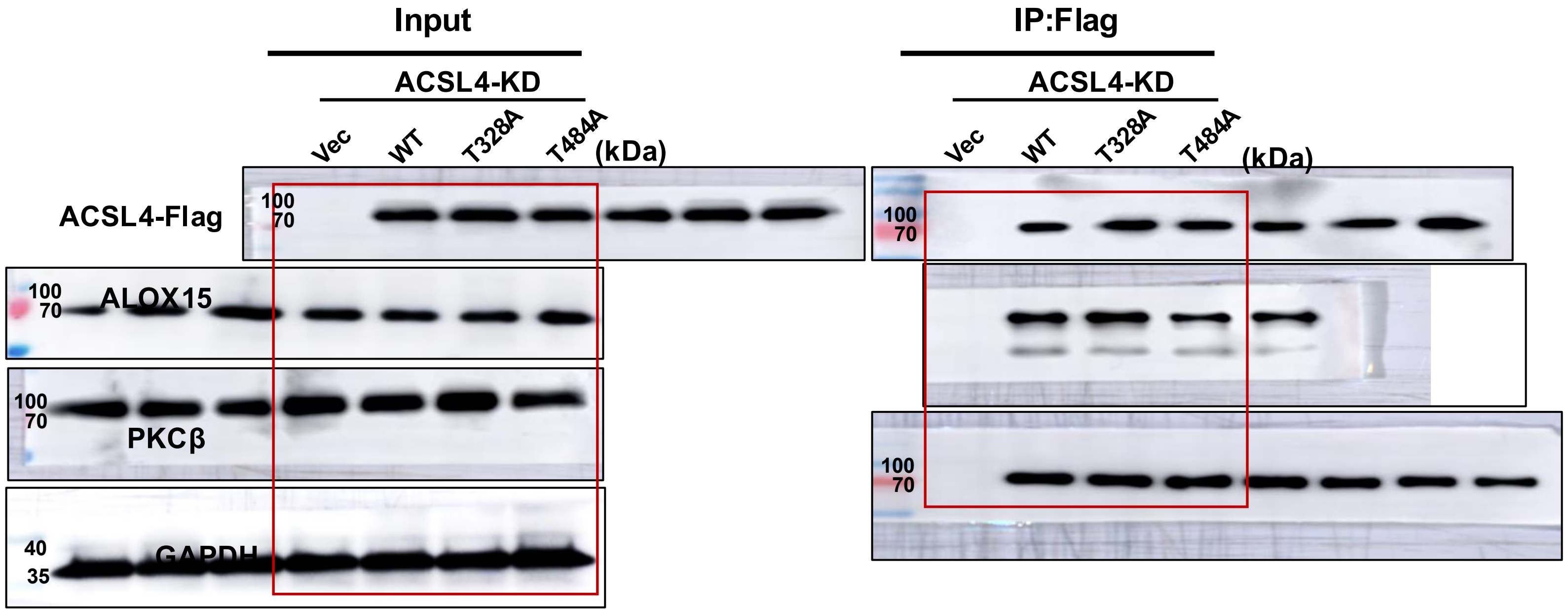

Figure 4I

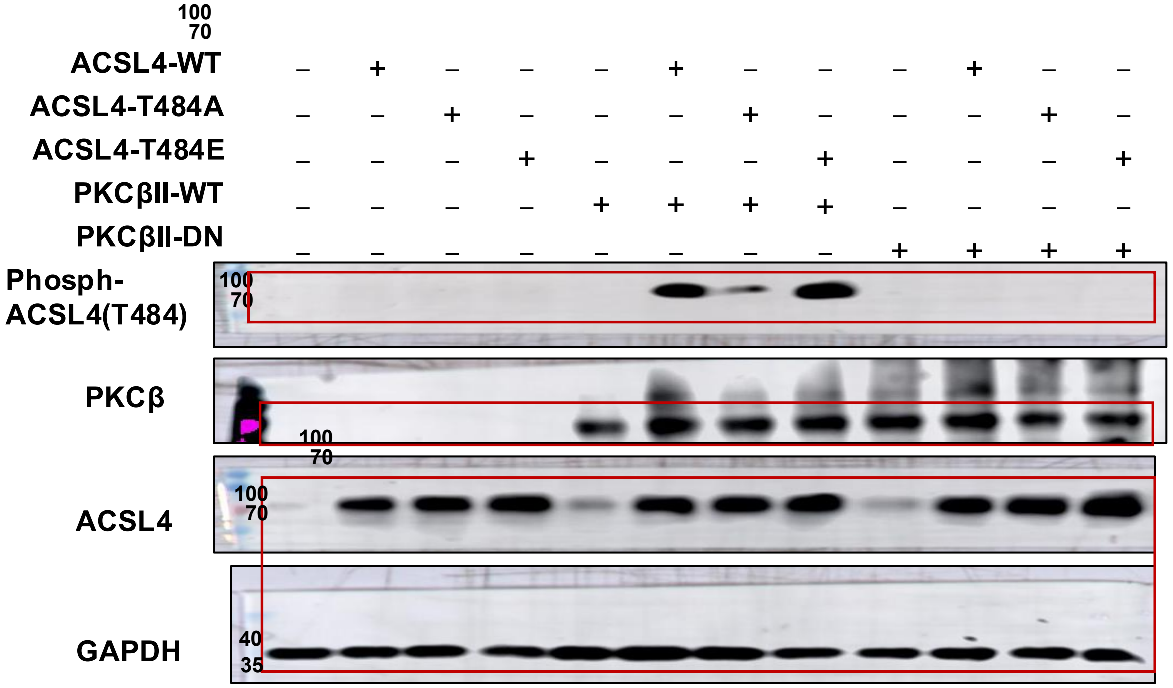

Figure 4H

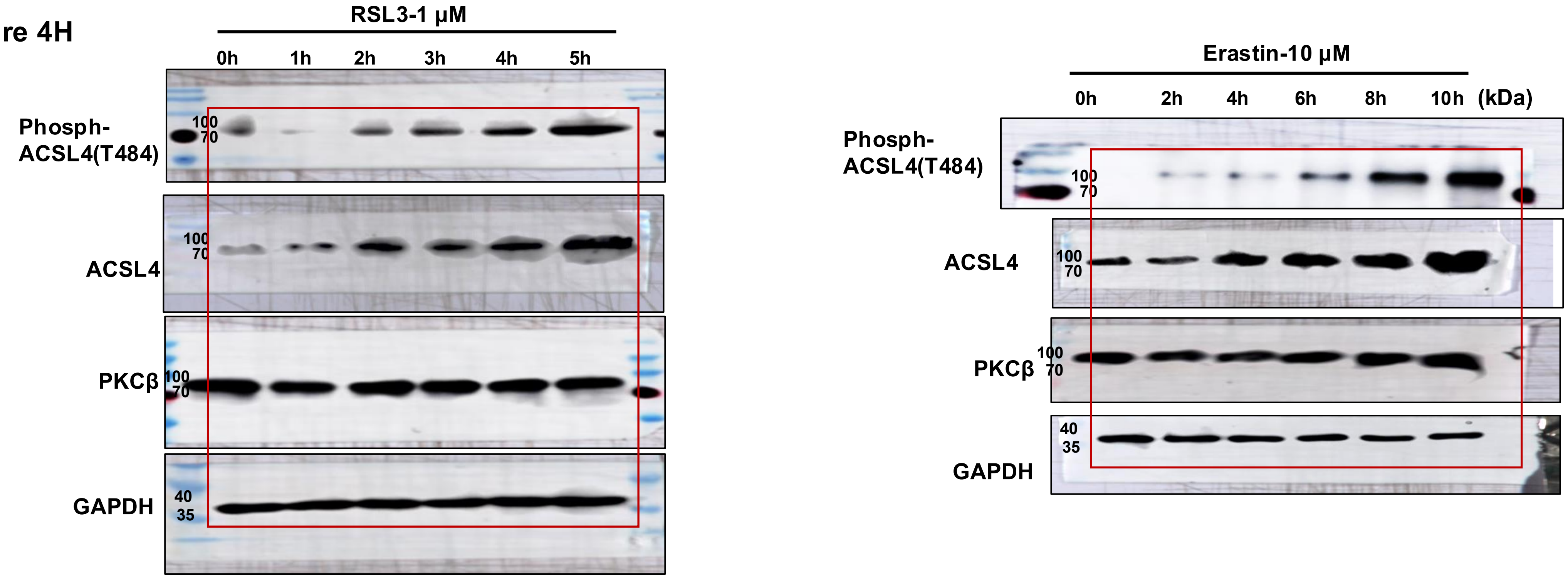

RSL3-1 μM
0h
1h
2h
3h
4h
5h
Phosph-ACSL4(T484)
ACSL4
PKCβ
GAPDH
100
70
40
35
Erastin-10 μM
0h
2h
4h
6h
8h
10h
(kDa)

Figure S4A

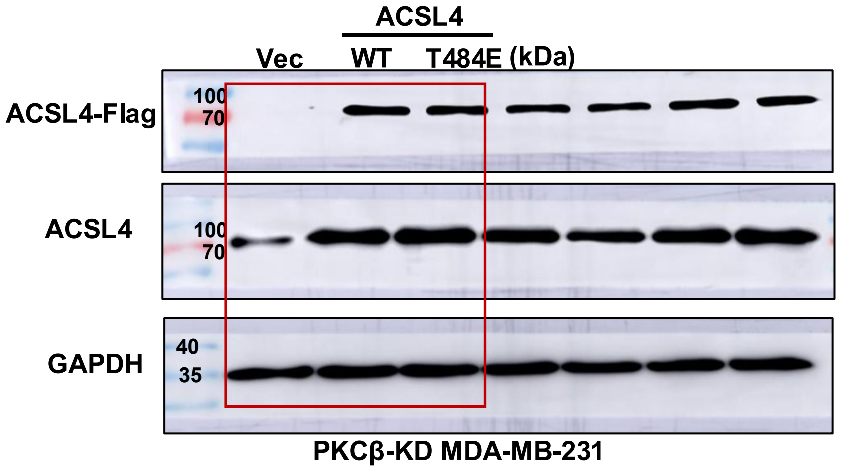

Figure S4E

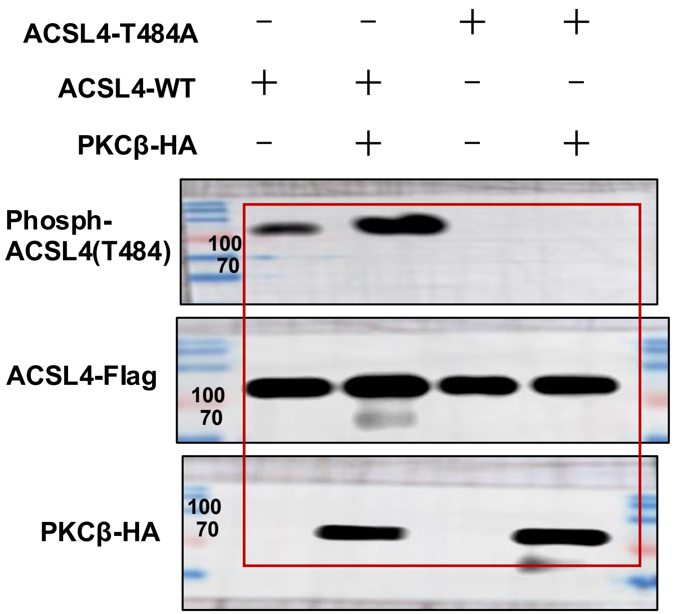

Figure S4D

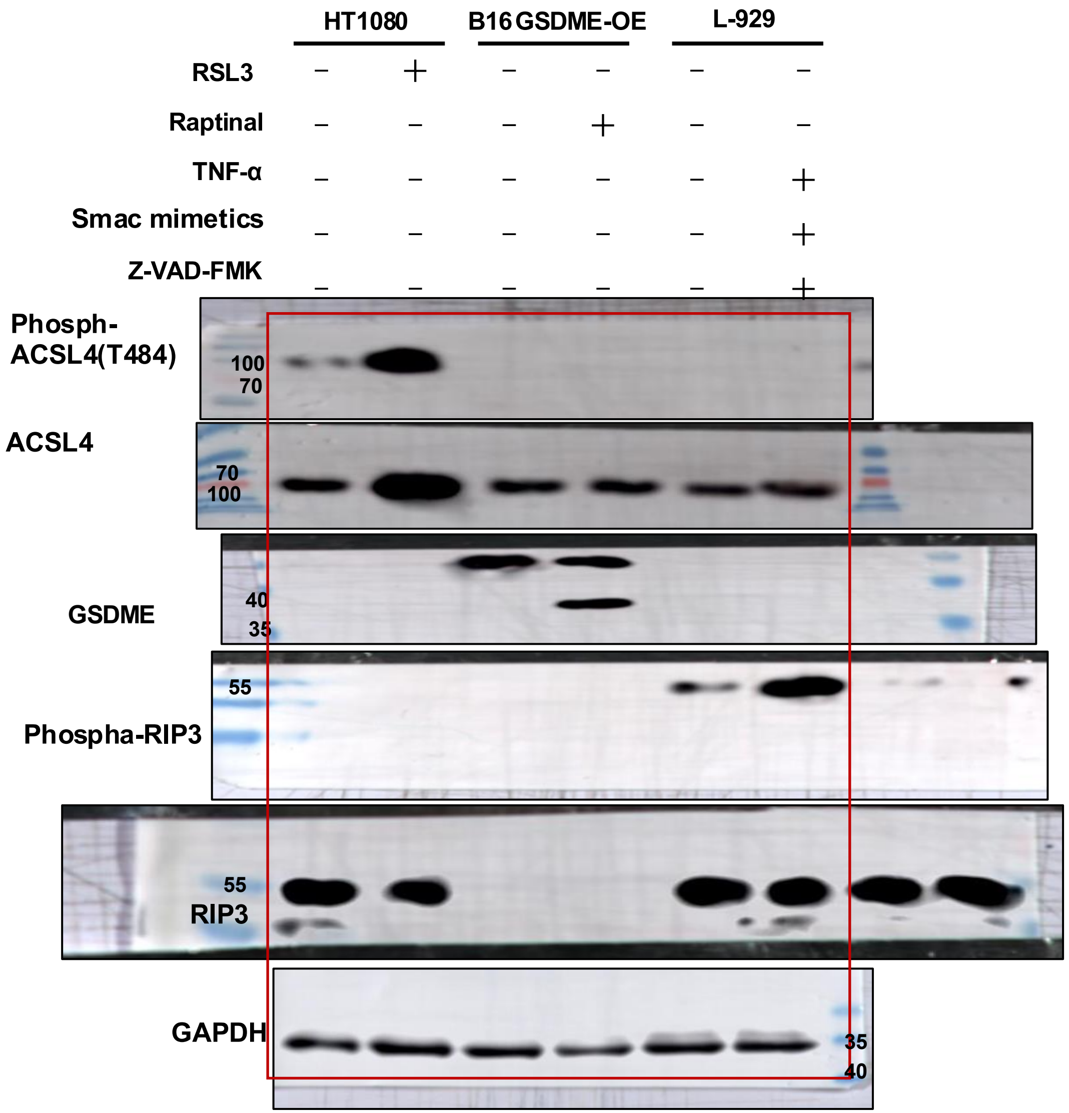

Figure 5A

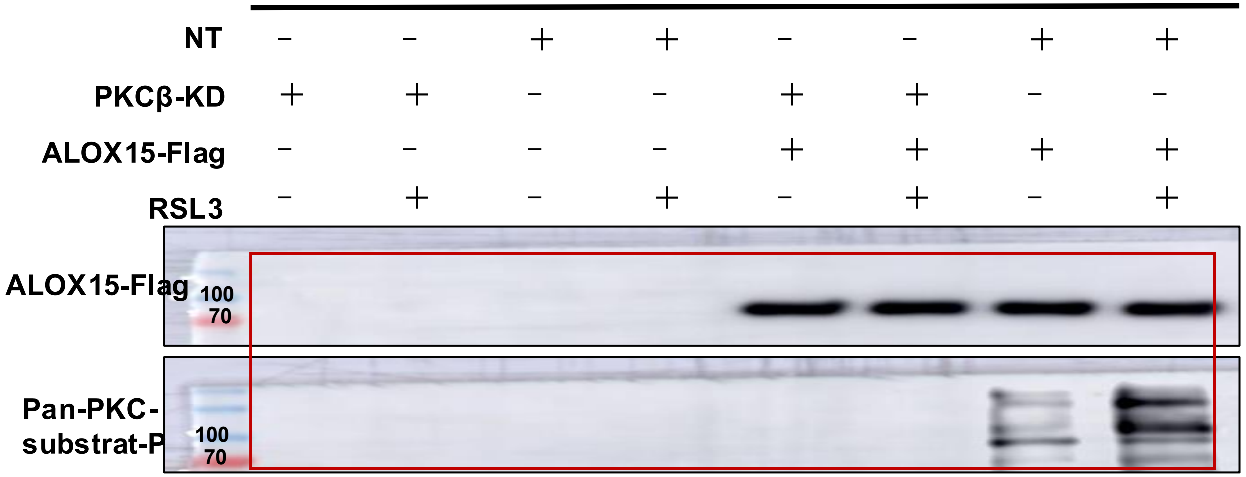

Figure 5D

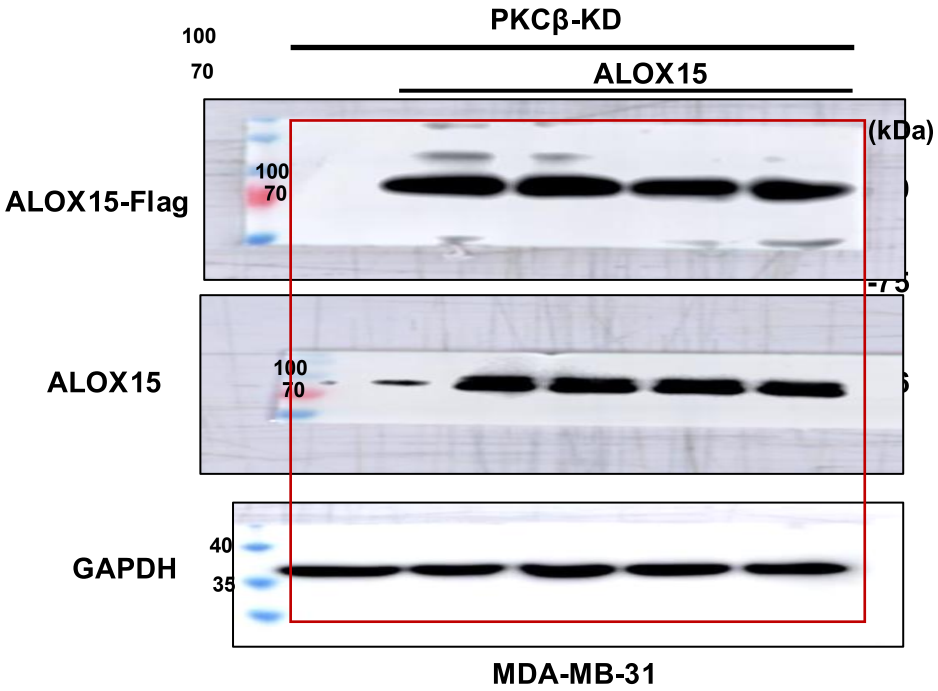

Figure 5B

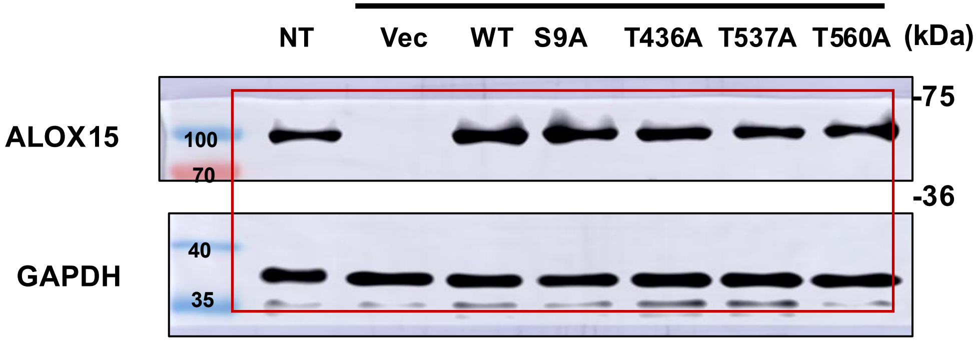

Figure 5C

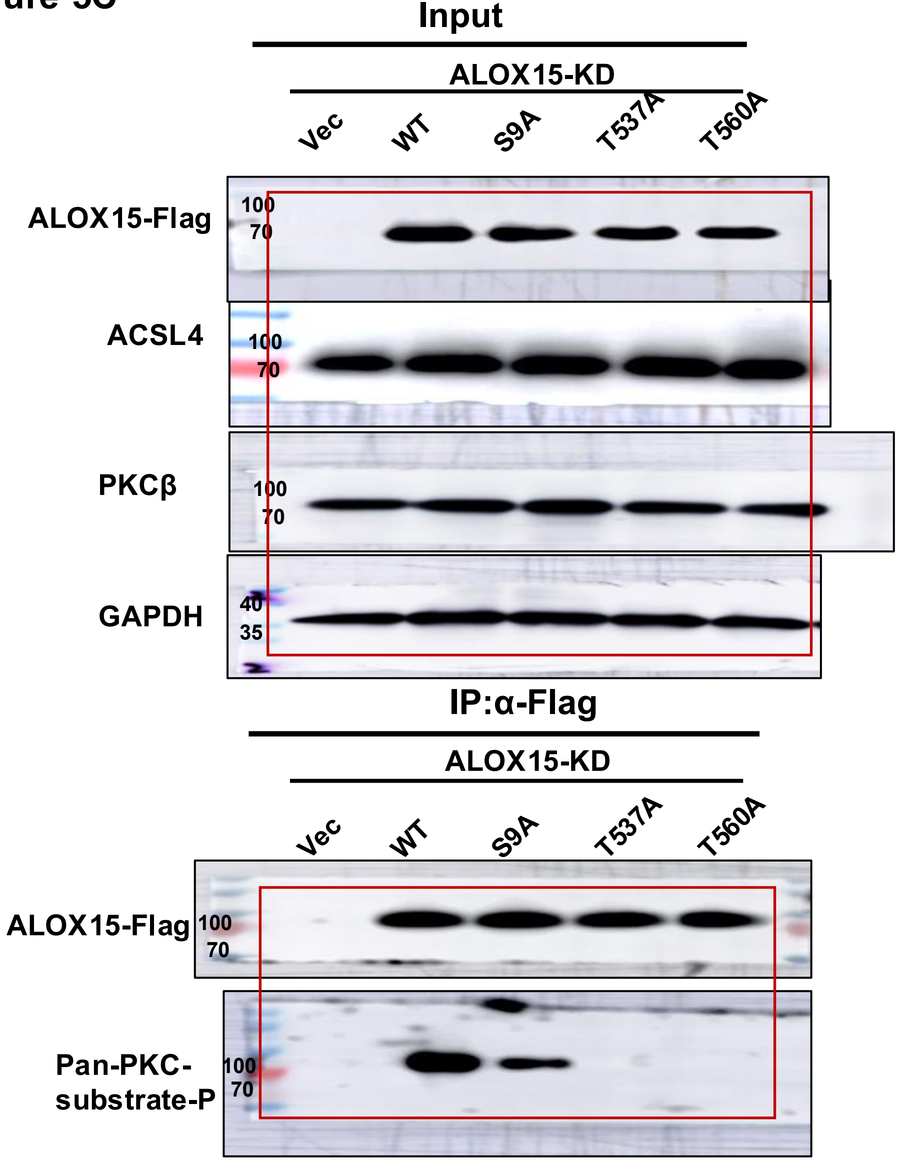
Input
ALOX15-KD
Vec
WT
S9A
T537A
T560A
ALOX15-Flag
100
70
ACSL4
100
70
PKCβ
100
70
GAPDH
40
35
IP:α-Flag
ALOX15-KD
Vec
WT
S9A
T537A
T560A
ALOX15-Flag
100
70
Pan-PKC-substrate-P
100
70

Figure 5E

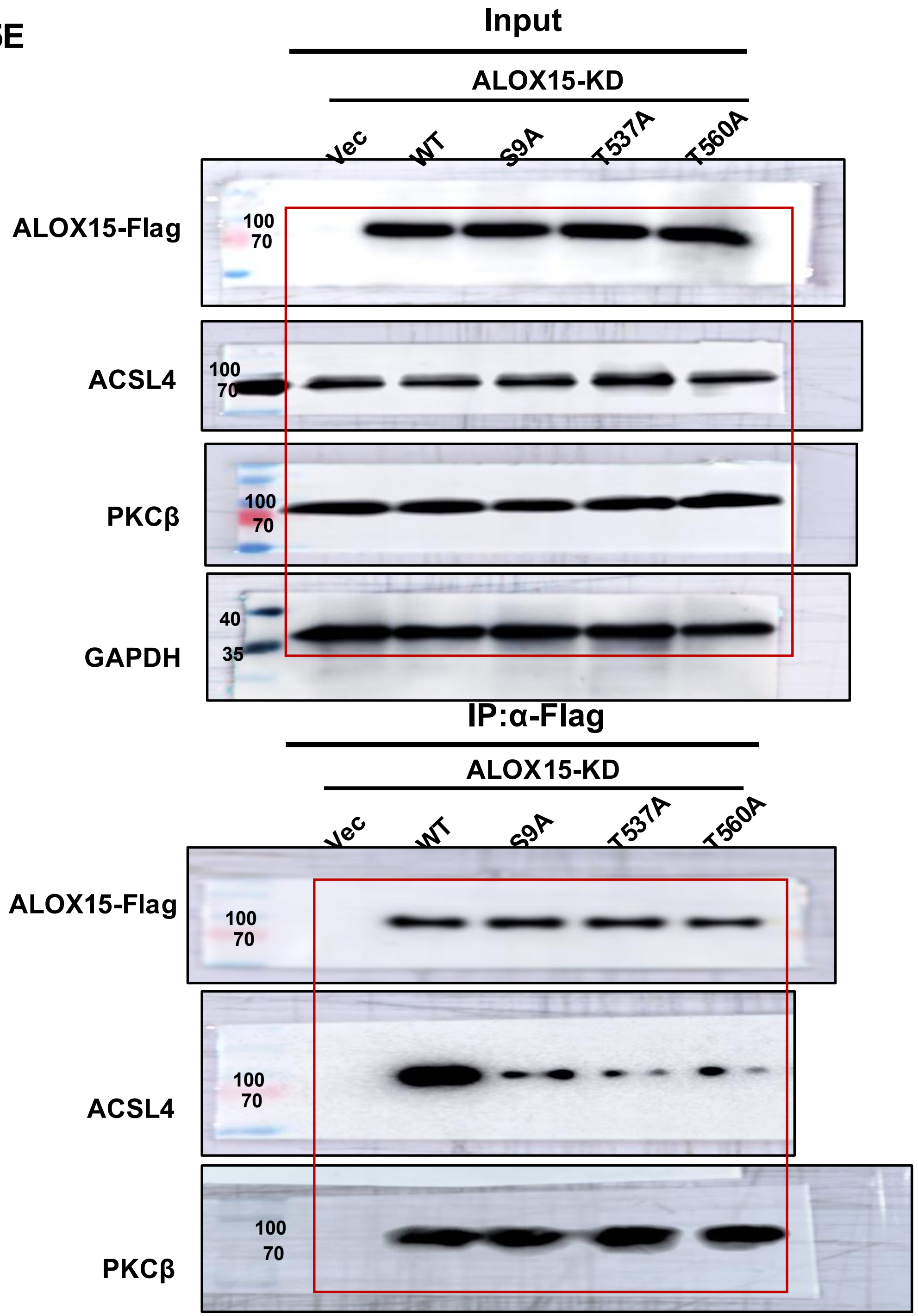

Figure 5F

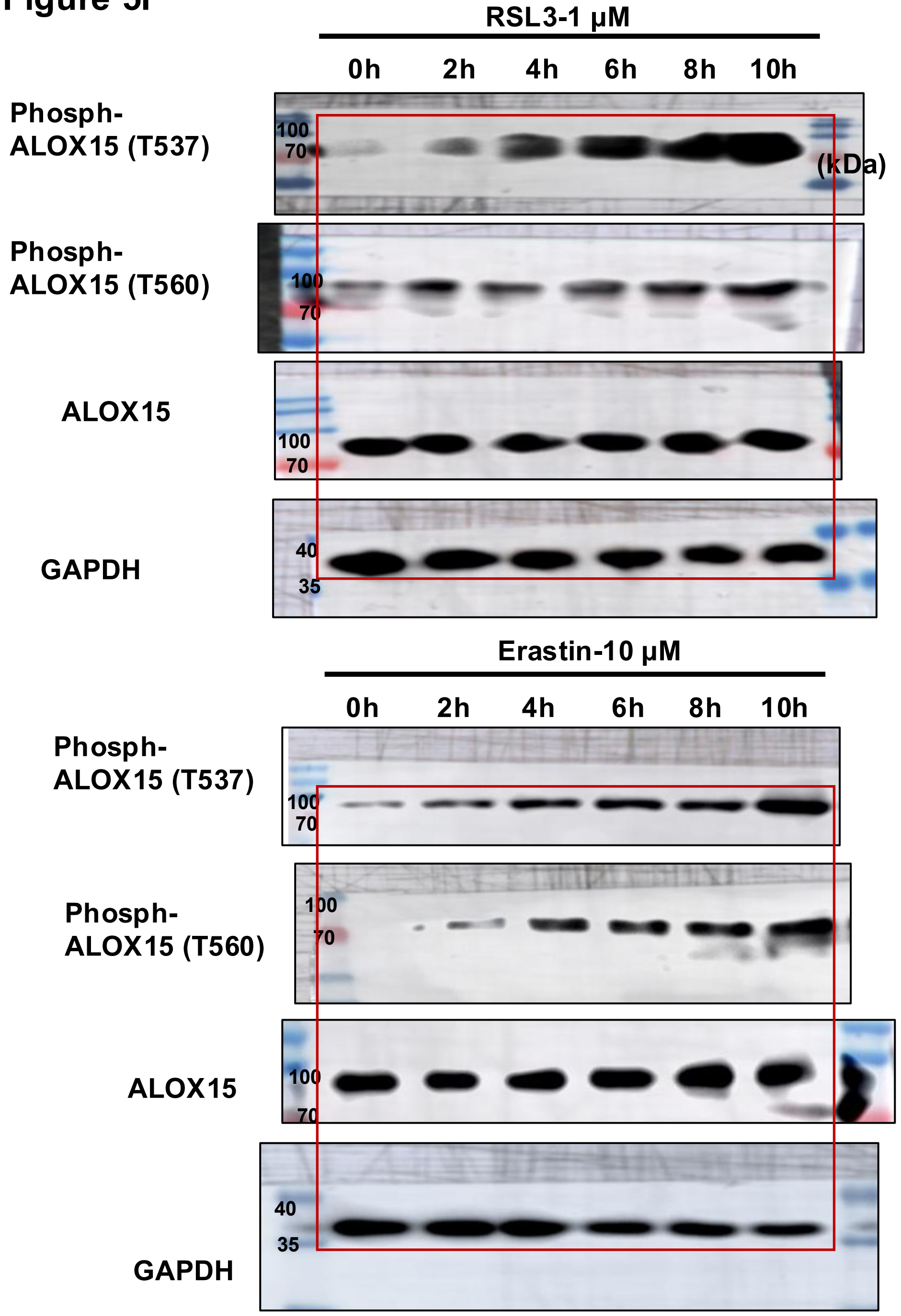

Figure 5G

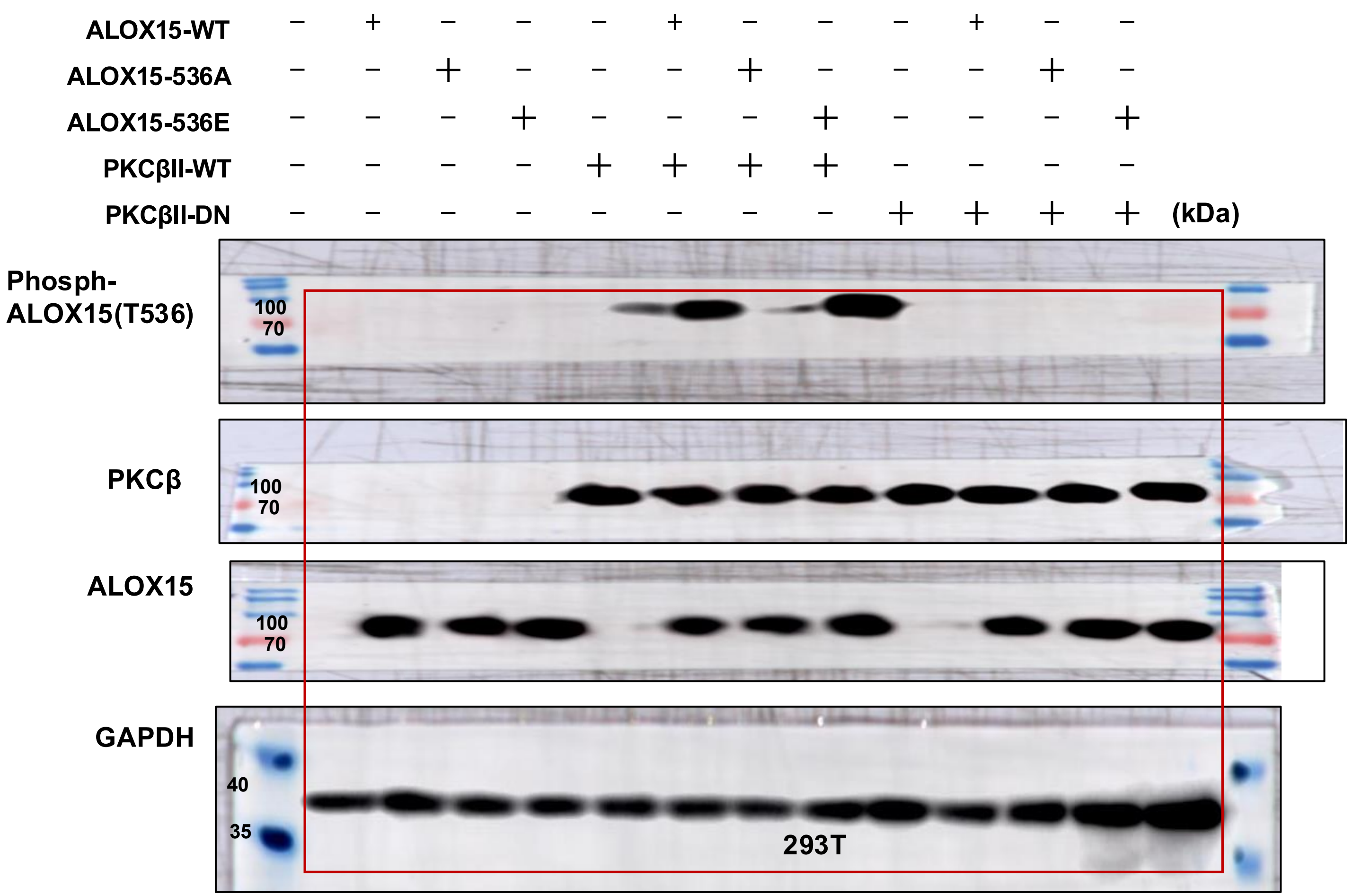

ALOX15-WT
ALOX15-536A
ALOX15-536E
PKCβII-WT
PKCβII-DN
(kDa)
Phosph-ALOX15(T536)
100
70
PKCβ
100
70
ALOX15
100
70
GAPDH
40
35
293T

Figure 5H

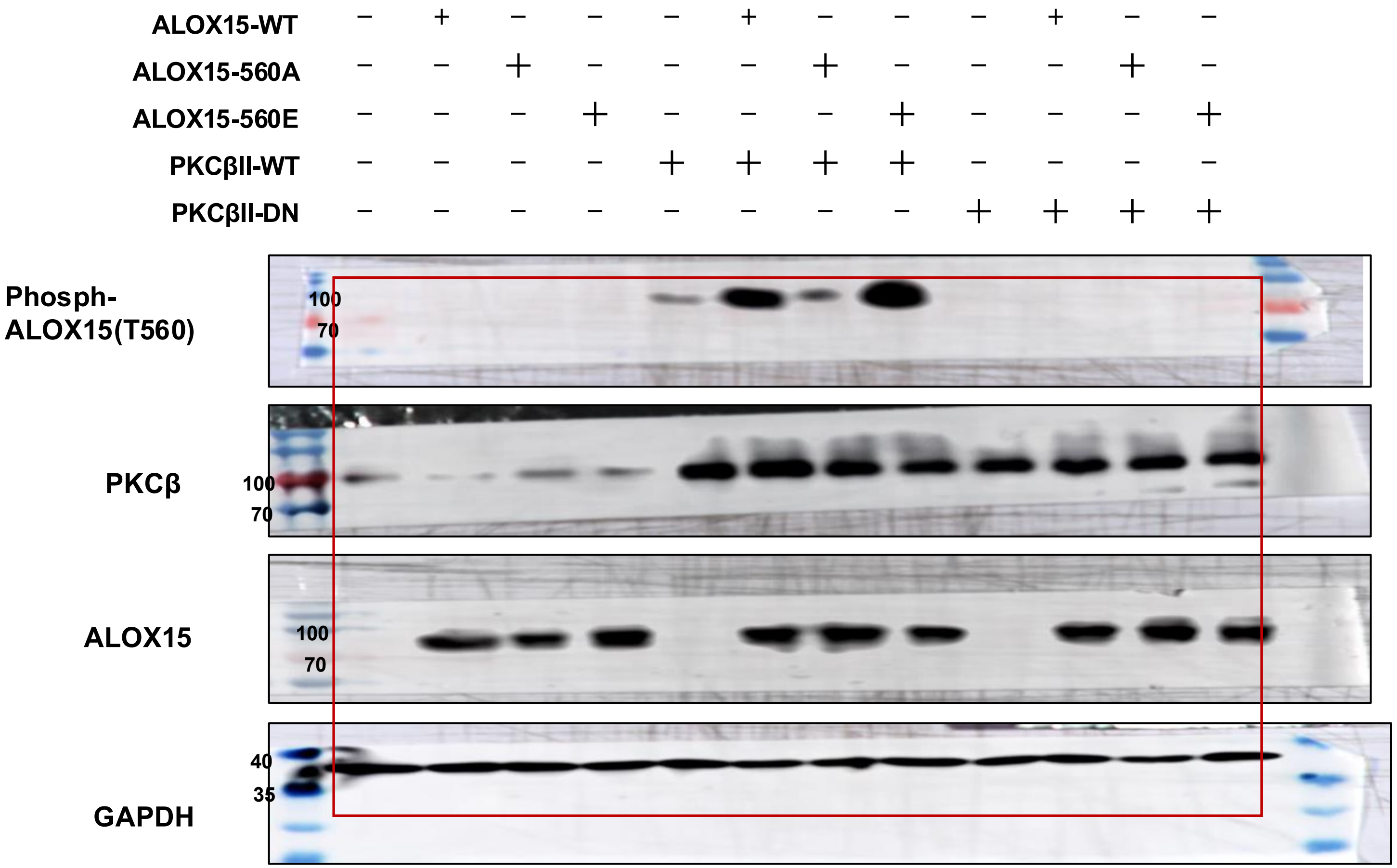
ALOX15-WT
ALOX15-560A
ALOX15-560E
PKCβII-WT
PKCβII-DN
Phosph-ALOX15(T560)
100
70
PKCβ
100
70
ALOX15
100
70
GAPDH
40
35

Figure S5A

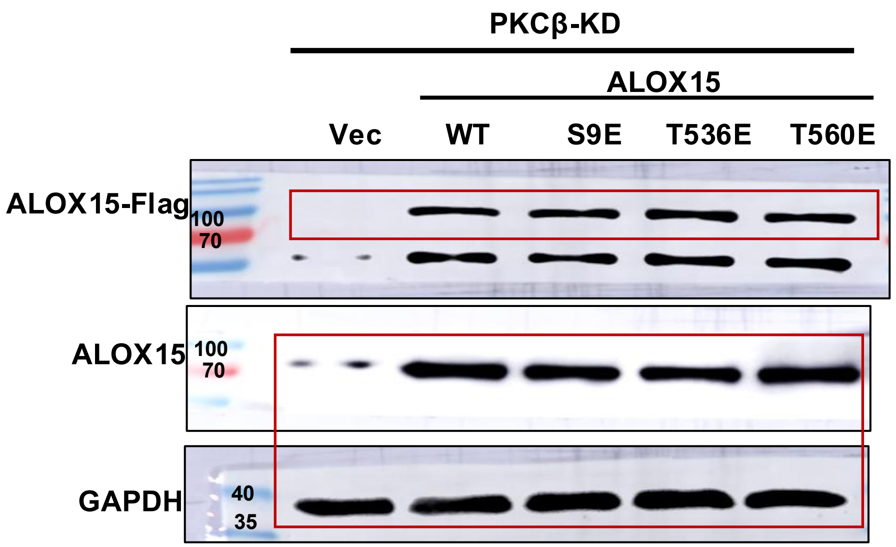

Figure S5E

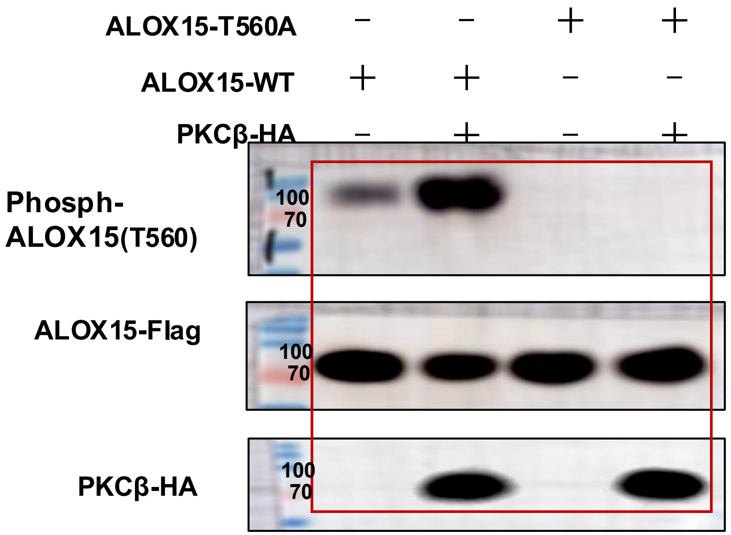

Figure S5F

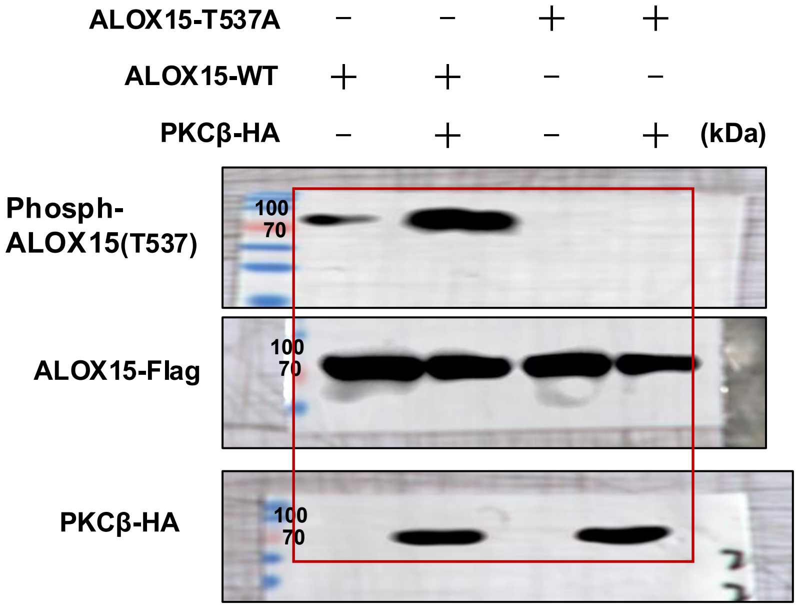

Figure S5G

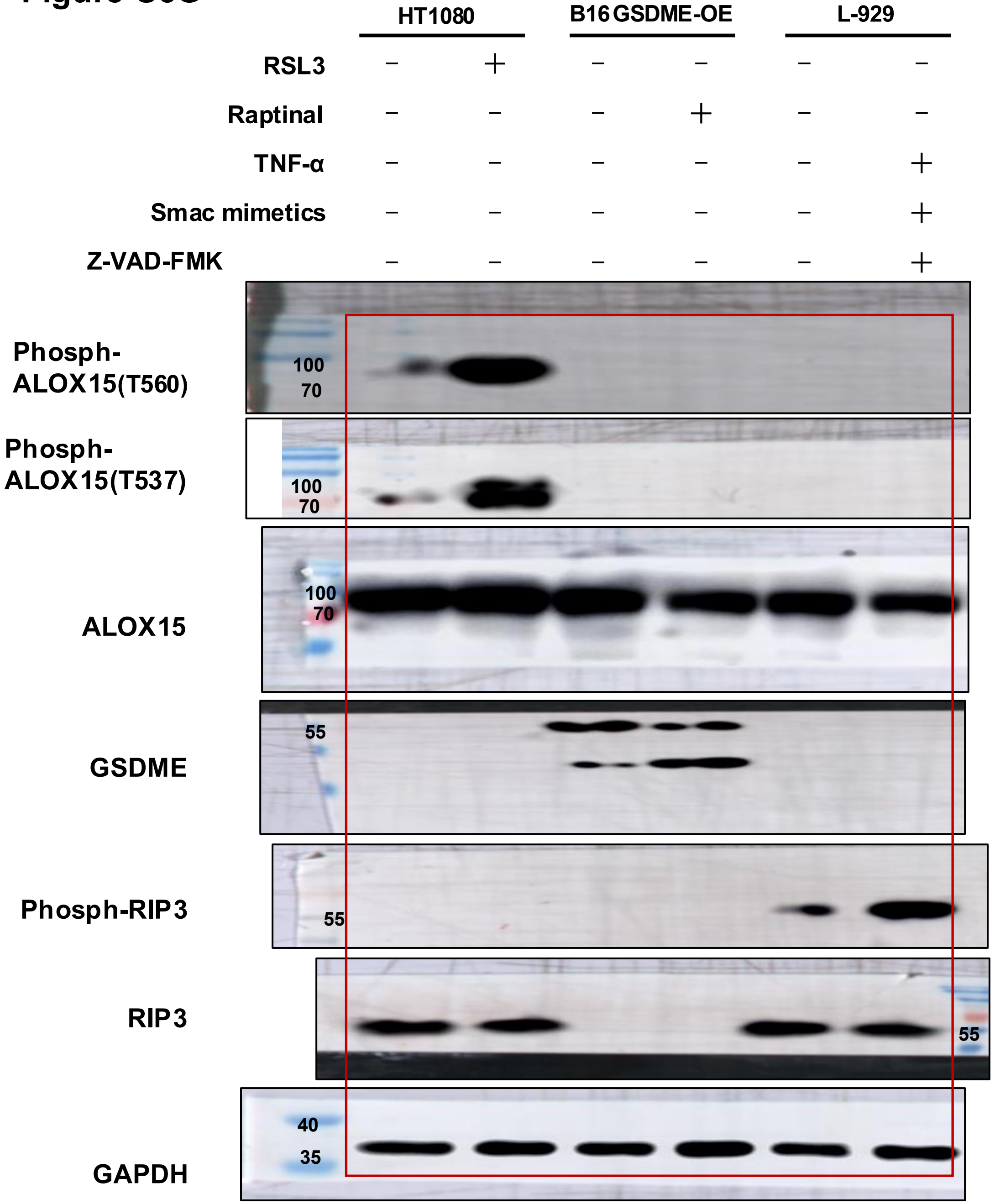

Figure 6D

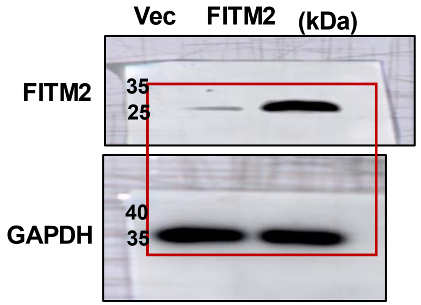

Figure 6E

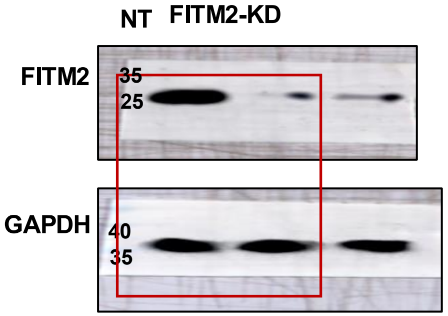

Figure S6H

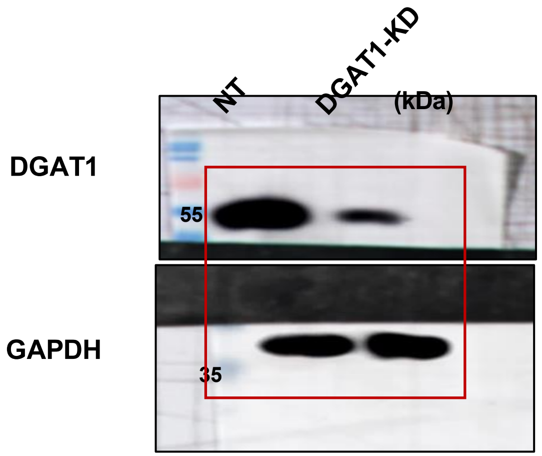

Figure S6I

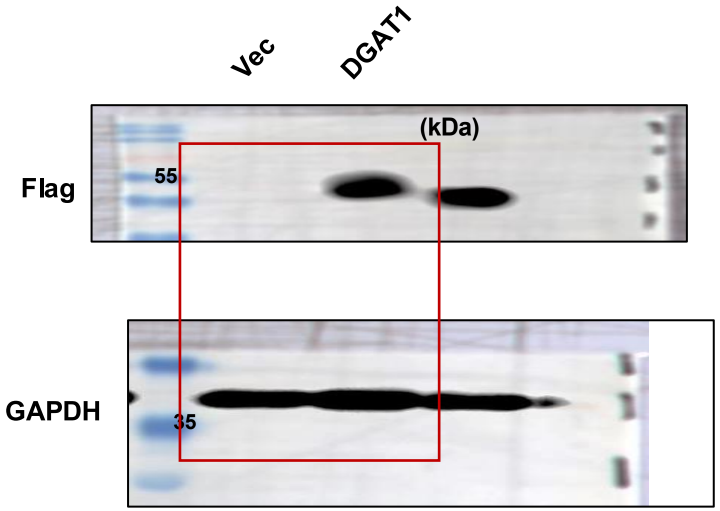

Figure S6J

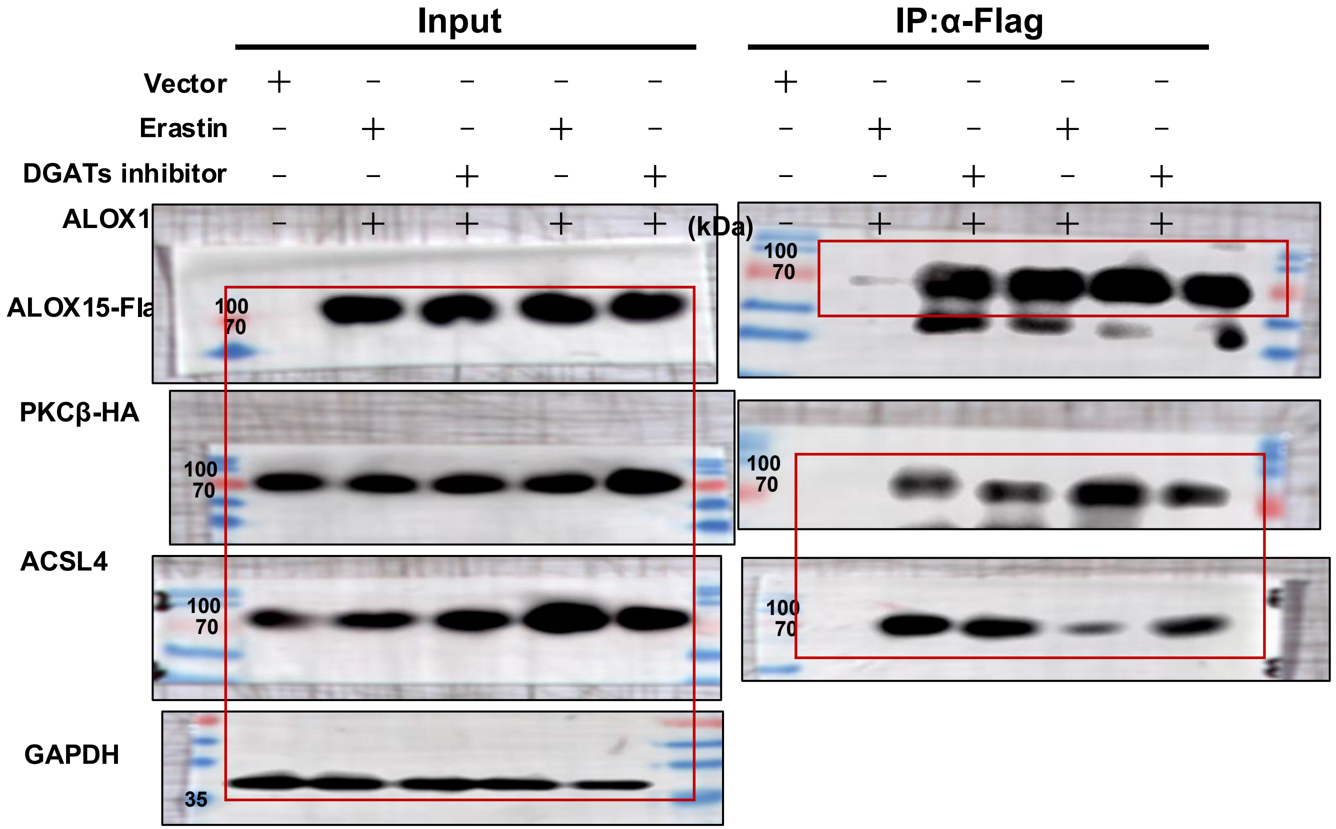

Figure S6K

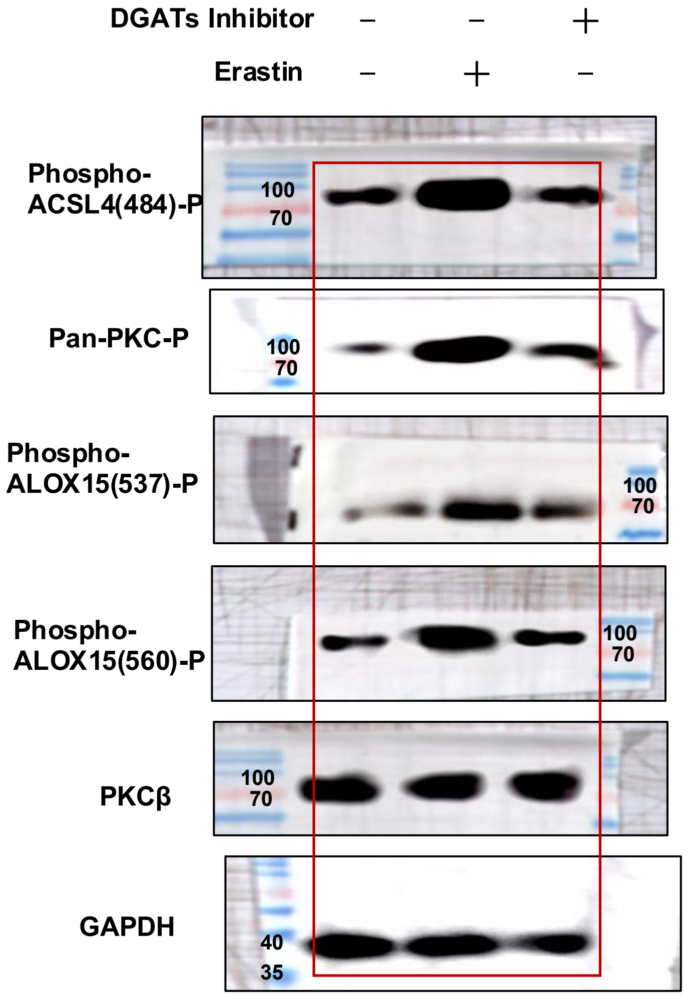

Figure 7B

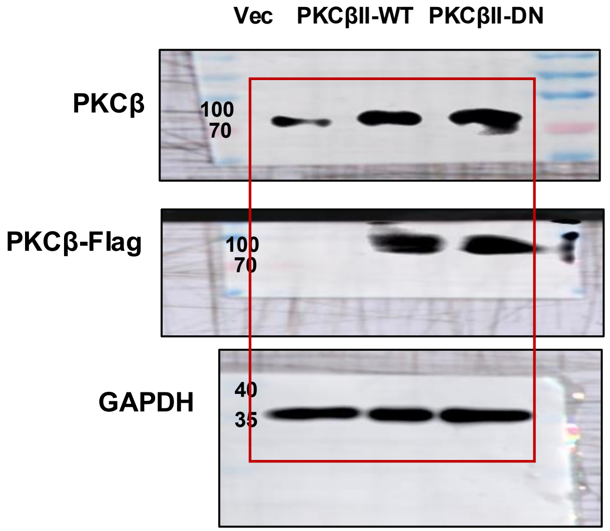

Figure 8F

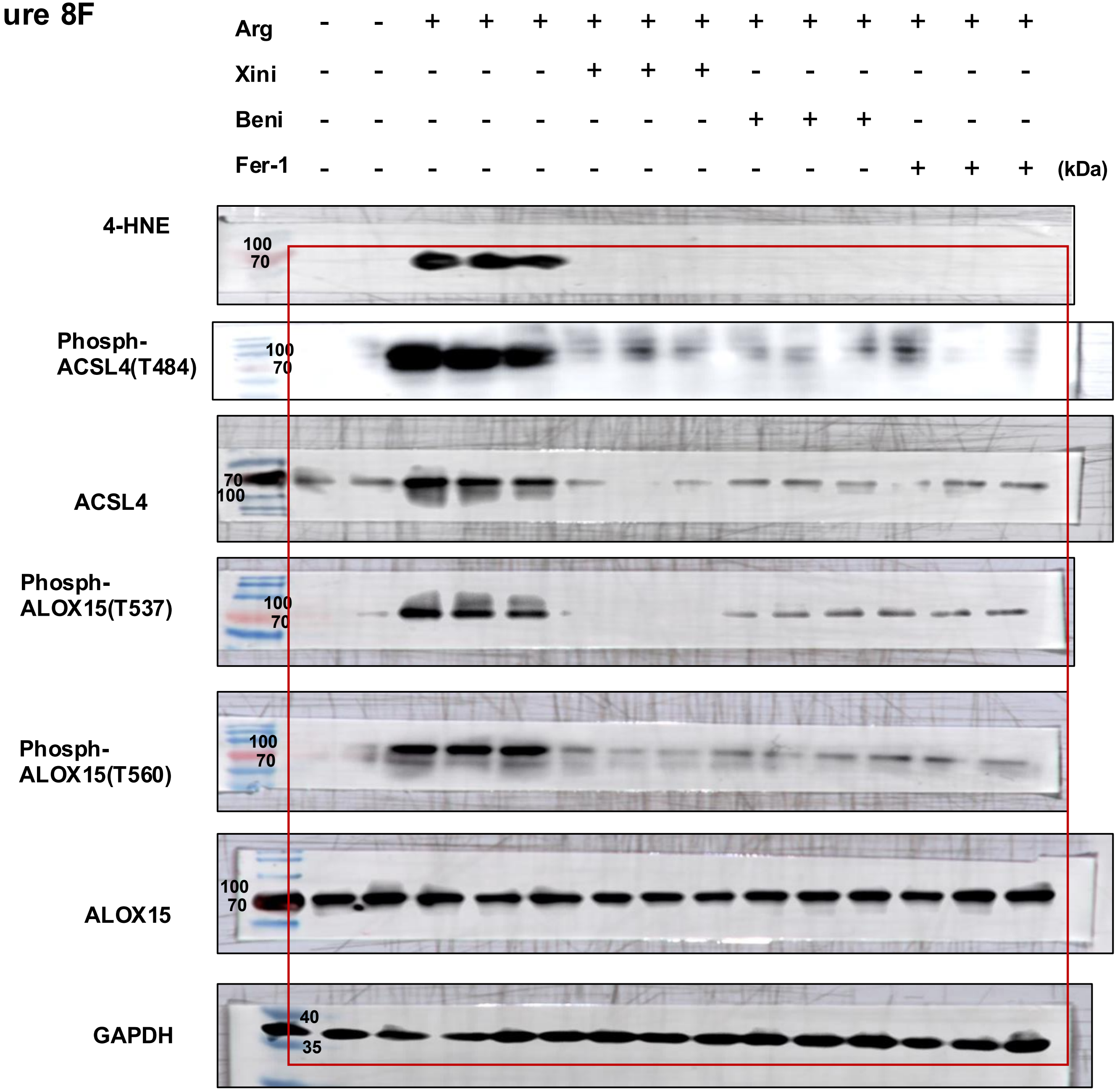

Arg - - + + + + + + + + + + + +
Xini - - - - - + + + - - - - - -
Beni - - - - - - - - + + + - - -
Fer-1 - - - - - - - - - - - + + +
(kDa)
4-HNE
100
70
Phosph-ACSL4(T484)
100
70
ACSL4
70
100
Phosph-ALOX15(T537)
100
70
Phosph-ALOX15(T560)
100
70
ALOX15
100
70
GAPDH
40
35

Figure 8F

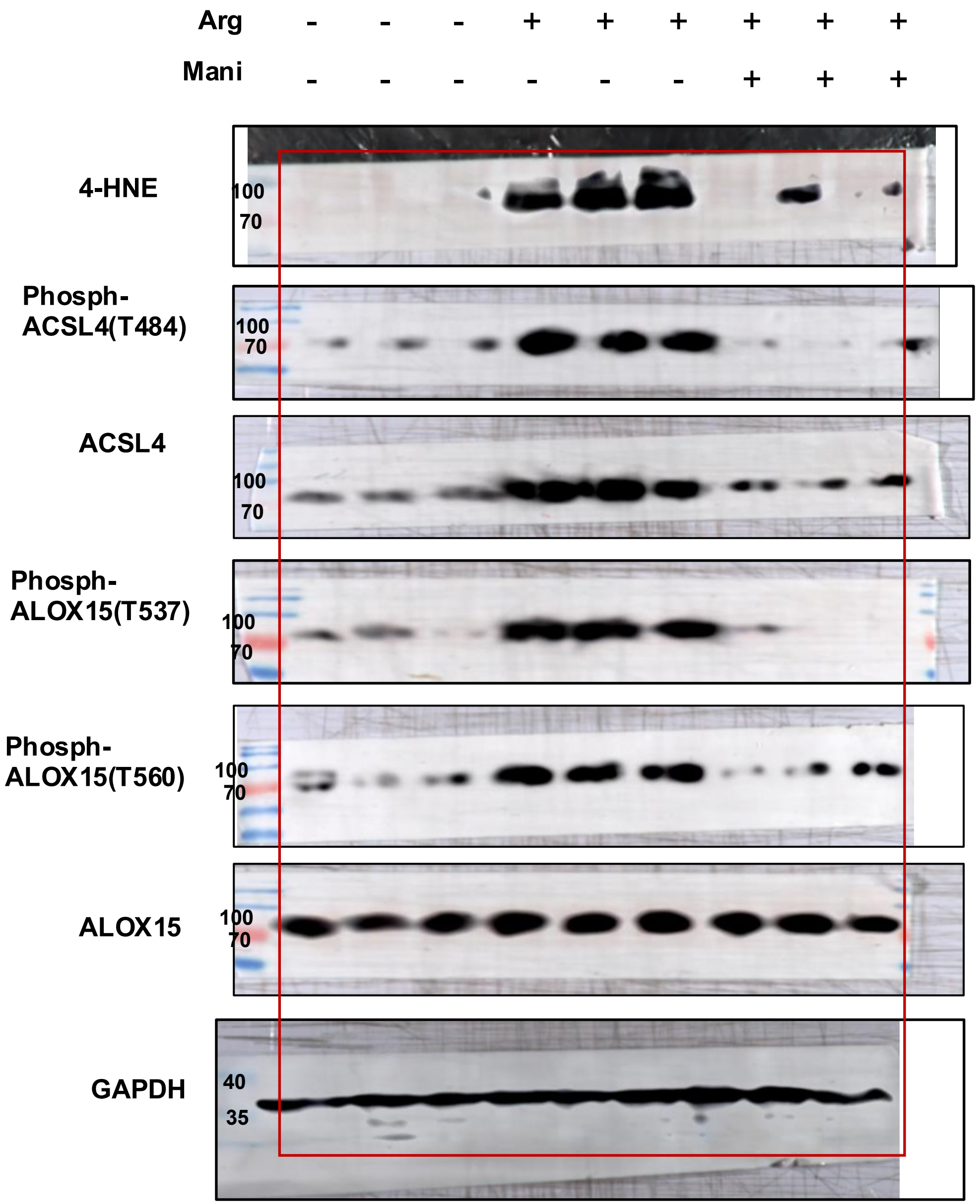

Arg - - - + + + + + +
Mani - - - - - - + + +
4-HNE
100
70
Phosph-ACSL4(T484)
ACSL4
Phosph-ALOX15(T537)
Phosph-ALOX15(T560)
ALOX15
GAPDH
40
35

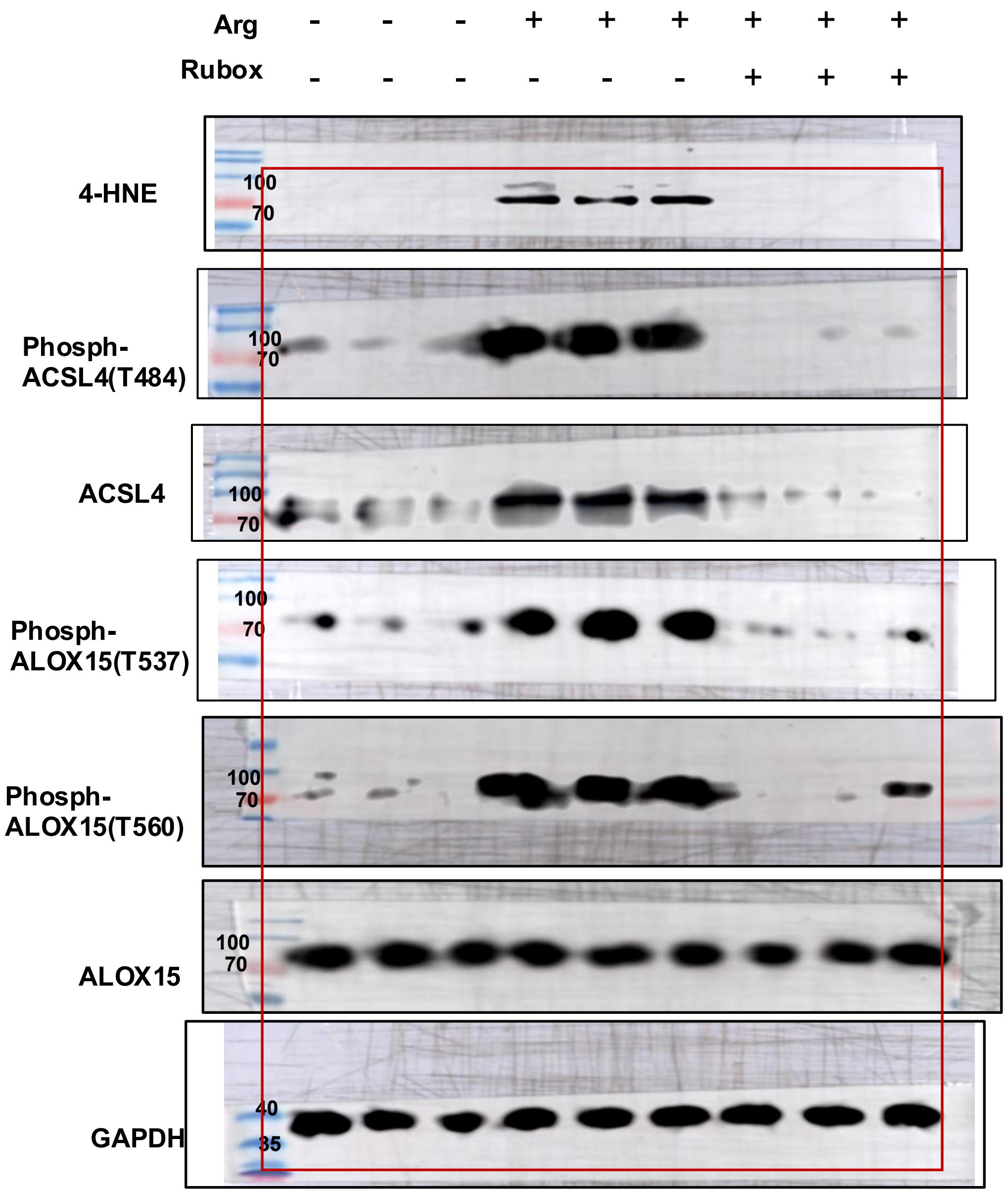

Arg - - - + + + + + +
Rubox - - - - - - + + +
4-HNE
100
70
Phosph-ACSL4(T484)
ACSL4
Phosph-ALOX15(T537)
Phosph-ALOX15(T560)
ALOX15
GAPDH
40
35
